# Temporally distinct 3D multi-omic dynamics in the developing human brain

**DOI:** 10.1038/s41586-024-08030-7

**Published:** 2024-10-09

**Authors:** Matthew G. Heffel, Jingtian Zhou, Yi Zhang, Dong-Sung Lee, Kangcheng Hou, Oier Pastor-Alonso, Kevin D. Abuhanna, Joseph Galasso, Colin Kern, Chu-Yi Tai, Carlos Garcia-Padilla, Mahsa Nafisi, Yi Zhou, Anthony D. Schmitt, Terence Li, Maximilian Haeussler, Brittney Wick, Martin Jinye Zhang, Fangming Xie, Ryan S. Ziffra, Eran A. Mukamel, Eleazar Eskin, Tomasz J. Nowakowski, Jesse R. Dixon, Bogdan Pasaniuc, Joseph R. Ecker, Quan Zhu, Bogdan Bintu, Mercedes F. Paredes, Chongyuan Luo

**Affiliations:** 1 Department of Human Genetics, University of California, Los Angeles, Los Angeles, CA USA; 2 Bioinformatics Interdepartmental Program, University of California, Los Angeles, Los Angeles, CA USA; 3 Genomic Analysis Laboratory, The Salk Institute for Biological Studies, La Jolla, CA USA; 4 Bioinformatics and Systems Biology Program, University of California, San Diego, La Jolla, CA USA; 5 Arc Institute, Palo Alto, CA USA; 6 Department of Biomedical Sciences, Seoul National University Graduate School, Seoul, Republic of Korea; 7 Genomic Medicine Institute, Medical Research Center, Seoul National University, Seoul, Republic of Korea; 8 Department of Pathology and Laboratory Medicine, David Geffen School of Medicine, University of California, Los Angeles, Los Angeles, CA USA; 9 Department of Computational Medicine, David Geffen School of Medicine, University of California, Los Angeles, Los Angeles, CA USA; 10 Department of Neurology, University of California, San Francisco, San Francisco, CA USA; 11 Department of Biological Chemistry, David Geffen School of Medicine, University of California, Los Angeles, Los Angeles, CA USA; 12 Center for Epigenomics, Department of Cellular and Molecular Medicine, University of California, San Diego, La Jolla, CA USA; 13 Department of Bioengineering, University of California, San Diego, La Jolla, CA USA; 14 Arima Genomics, Carlsbad, CA USA; 15 Genomics Institute, University of California, Santa Cruz, Santa Cruz, CA USA; 16 Ray and Stephanie Lane Computational Biology Department, School of Computer Science, Carnegie Mellon University, Pittsburgh, PA USA; 17 Department of Epidemiology, Harvard T.H. Chan School of Public Health, Boston, MA USA; 18 Department of Cognitive Science, University of California, San Diego, La Jolla, CA USA; 19 Department of Neurological Surgery, University of California, San Francisco, San Francisco, CA USA; 20 Department of Anatomy, University of California, San Francisco, San Francisco, CA USA; 21 Department of Psychiatry and Behavioral Sciences, University of California, San Francisco, San Francisco, CA USA; 22 Eli and Edythe Broad Center for Regeneration Medicine and Stem Cell Research, University of California, San Francisco, San Francisco, CA USA; 23 Department of Computational Medicine, University of California, Los Angeles, Los Angeles, CA USA; 24 Gene Expression Laboratory, The Salk Institute for Biological Studies, La Jolla, CA USA; 25 Howard Hughes Medical Institute, The Salk Institute for Biological Studies, La Jolla, CA USA; 26 Weill Institute for Neurosciences, University of California, San Francisco, San Francisco, CA USA; 27 Neuroscience Graduate Program, University of California, San Francisco, San Francisco, CA USA; 28 Developmental Stem Cell Biology, University of California, San Francisco, San Francisco, CA USA

**Keywords:** Chromatin structure, Cell type diversity

## Abstract

The human hippocampus and prefrontal cortex play critical roles in learning and cognition^1,2^, yet the dynamic molecular characteristics of their development remain enigmatic. Here we investigated the epigenomic and three-dimensional chromatin conformational reorganization during the development of the hippocampus and prefrontal cortex, using more than 53,000 joint single-nucleus profiles of chromatin conformation and DNA methylation generated by single-nucleus methyl-3C sequencing (snm3C-seq3)^3^. The remodelling of DNA methylation is temporally separated from chromatin conformation dynamics. Using single-cell profiling and multimodal single-molecule imaging approaches, we have found that short-range chromatin interactions are enriched in neurons, whereas long-range interactions are enriched in glial cells and non-brain tissues. We reconstructed the regulatory programs of cell-type development and differentiation, finding putatively causal common variants for schizophrenia strongly overlapping with chromatin loop-connected, cell-type-specific regulatory regions. Our data provide multimodal resources for studying gene regulatory dynamics in brain development and demonstrate that single-cell three-dimensional multi-omics is a powerful approach for dissecting neuropsychiatric risk loci.

## Abstract (web summary)

Using a single-nucleus multi-omics approach, a study jointly profiles the reorganization of the epigenome and the three-dimensional chromatin conformation during the development of the human hippocampus and prefrontal cortex.

## Main

The adult human brain contains hundreds of cell types that exhibit an extraordinary diversity of molecular, morphological, anatomic and functional characteristics^4–6^. Although most cortical neurons are generated during the first and second trimesters, the highly distinct molecular signatures of cell types emerge between the third trimester and adolescence^7–9^. Single-cell and bulk transcriptome analyses implicated marked gene expression remodelling in late prenatal and early postnatal development^10,11^. The pervasive transcriptome dynamics during human brain development is associated with genome-wide reconfiguration of the DNA methylome and chromatin conformation^12–16^. The brain-specific non-CG methylation emerges in the human dorsal prefrontal cortex (PFC) during prenatal development in a cell-type-specific pattern, with the average level of non-CG methylation increasing through adolescence^12,17^. Recent studies have uncovered the remodelling of chromatin architecture during the early postnatal development of mouse and human brains^15,18^, as well as extensive chromatin conformational diversity across brain regions and cell types in the adult human brain^19^. However, the dynamic trajectory of DNA methylation and chromatin conformation changes have not been characterized with single-cell resolution in prenatal human brain tissues and compared to those of postnatal development using infant and adult samples. This study investigated the developmental dynamics of human PFC and hippocampus (HPC) using the sequencing-based approach single-nucleus methyl-3C sequencing (snm3C-seq3) to jointly profile chromatin conformation and DNA methylation in single nuclei^3,19^, as well as the orthogonal multimodal chromatin tracing procedure, and RNA and protein imaging.

## Three-dimensional multi-omics of developing brains

**Fig. 1:**
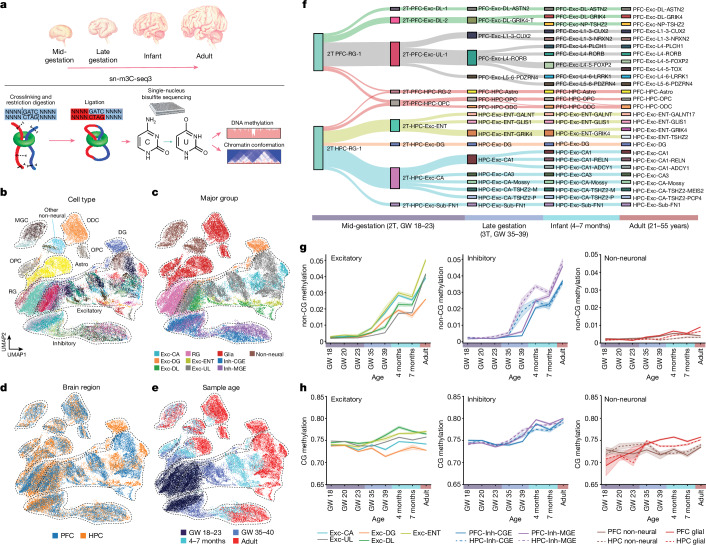
Profiling of epigenomic and chromatin conformation dynamics during human brain development using snm3C-seq3. **a**, Schematics of the study. Illustrations of developing human brain by Byron Ashley. **b**–**e**, Dimensionality reduction using uniform manifold approximation and projection (UMAP) distinguishes cell types (**b**), major cell lineages (**c**), brain regions (**d**) and developmental stages (**e**). Astro, astrocyte; CGE, caudal ganglionic eminence; DG, dentate gyrus; DL, deep layer; ENT; entorhinal cortex; Exc, excitatory neurons; Inh, inhibitory neurons; MGC, microglia; MGE, medial ganglionic eminence; ODC, oligodendrocyte; OPC, oligodendrocyte progenitor cell; UL, upper layer. **f**, Reconstructed developmental hierarchy of excitatory neurons and glial cells. 2T, second trimester or mid-gestation; 3T, third trimester or late gestation; NP, near-projecting; Sub, subiculum. **g**,**h**, Dynamics of genome-wide non-CG methylation (**g**) and CG methylation (**h**) during human brain development.

**Extended Data Fig. 1:**
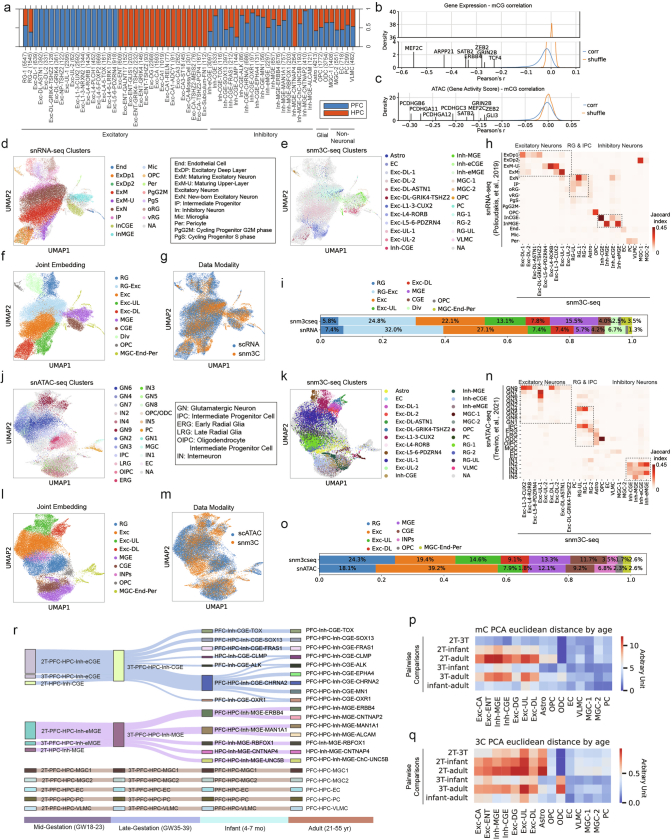
Multi-modal classification of brain cell types in developmental specimens. (a) Brain regional specificity of identified cell types. (b-c) Inversed correlations between gene expression and gene body CG methylation (b) and between chromatin accessibility (gene activity score) and gene body CG methylation (c). (d-g) UMAP dimensionality reduction of the integration of snm3C-seq3 and snRNA-seq datasets with the visualization of snRNA-seq clusters (d), snm3C-seq3 clusters (e), joint clusters (f), and assay type (g). (h) Comparison of cell type classification using snm3C-seq3 and snRNA-seq using a confusion matrix. (i) Comparison of cell type composition in snm3C-seq3 and snRNA-seq datasets. (j-m) UMAP of the Integration of snm3C-seq3 and snATAC-seq datasets with the visualization of snATAC-seq clusters (j), snm3C-seq3 clusters (k), joint clusters (l), and assay type (m). (n) Comparison of cell type classification using snm3C-seq3 and snATAC-seq using a confusion matrix. (o) Comparison of cell type composition in snm3C-seq3 and snATAC-seq datasets. (p-q) Genome-wide Euclidian distance of gene body CG methylation (p) and chromatin interaction (q) between developmental stages for each major cell type group. (r) Reconstructed developmental hierarchy of inhibitory neurons and non-neuronal cells.

**Extended Data Fig. 2:**
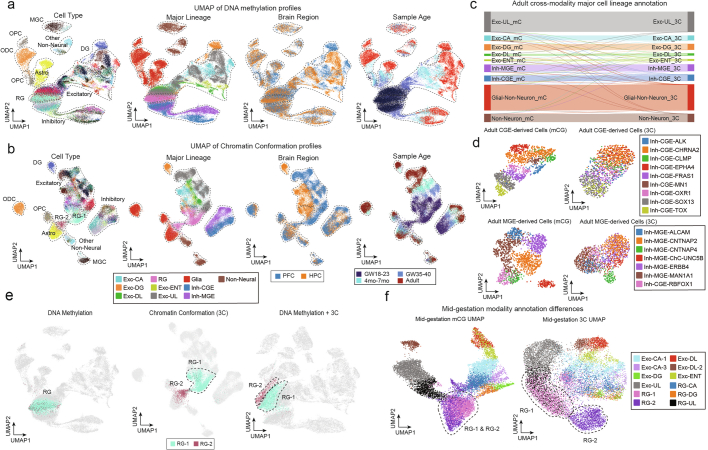
Comparison of brain cell type classification using DNA methylation and chromatin confirmation signatures. (a) Methylation dimensionality reduction using UMAP distinguishes, from left to right, cell types, major cell lineages, brain regions, and sample age groups. (b) Chromatin conformation dimensionality reduction using UMAP distinguishes, from left to right, cell types, major cell lineages, brain regions, and sample age groups. (c) Riverplot to show extensive consistency in adult major cell lineage annotation between DNA methylation and chromatin conformation modalities. (d) Dimensionality reduction using UMAP shows resolution difference in cell-type classification using DNA methylation and chromatin conformation modalities in adult inhibitory neurons; CGE-derived (top), MGE-derived (bottom). (e) Showing distinction of RG-1 and RG-2 populations in methylation space (left), chromatin conformation space (middle), and joint dimensionality reduction space (right). (f) Dimensionality reduction of z-scored CG methylation feature matrix for cells from mid-gestational brains (left). Chromatin conformation dimensionality reduction of cells from mid-gestational brains (right).

We generated 29,691 snm3C-seq3 profiles (including 3,321 previously published profiles^3^) from 13 developing and adult human frontal cortex samples and 23,372 snm3C-seq3 profiles from 9 HPC samples using the newly devised snmC-seq3 method for single-cell methylome library preparation (Fig. 1a and Supplementary Tables 1 and 2). The quality of snm3C-seq3 3C (chromatin conformation capture) profiles is consistent across brain specimens (Supplementary Note 1). The multimodal information profiled by snm3C-seq3 was used at various resolutions. To classify brain cell types, we quantified CG and non-CG methylation and 3C information in individual cells at 100 kilobase (kb) resolution. In downstream analyses, aggregated methylation profiles at a cell-type level were used for differentially methylated region (DMR) analysis at base resolution, whereas aggregated 3C profiles were used to identify domain boundaries at 25 kb resolution and loop calling at 10 kb resolution. We identified 139 cell populations across all developmental stages by fusing three data modalities: CG and non-CG methylation and chromatin conformation (Fig. 1b and Supplementary Table 3). These cell types are organized into 10 major groups (Fig. 1c). Excitatory neurons had distinct epigenomic types in the human PFC and HPC, which is consistent with their spatially separated in situ neurogenesis (Fig. 1d and Extended Data Fig. 1a). By contrast, inhibitory neurons and non-neuronal cell types are broadly shared between the two brain regions (Fig. 1d and Extended Data Fig. 1a). Our previous works found strong agreements between adult brain cell types identified using snm3C-seq and single-nucleus RNA sequencing^20^. Taking advantage of the inverse correlation between CG methylation and gene expression^20^, and between CG methylation and the chromatin accessibility gene activity score (Extended Data Fig. 1b,c), we integrated snm3C-seq3 with single-nucleus RNA sequencing^21^ (Extended Data Fig. 1d–i), or chromatin accessibility profiling^14^ (Extended Data Fig. 1j–o), and found that each data modality identified similar cell types in the prenatal frontal cortex^14,21^ (Extended Data Fig. 1h,i,n,o). Neurons and neural progenitor-derived glial cells were strongly separated by developmental stages on the basis of their methylation and chromatin conformation patterns, whereas non-neural cell types showed similar epigenomic patterns across development (Fig. 1e and Extended Data Fig. 1p–q). The developmental trajectories of cortical and hippocampal cell types were reconstructed using shared CG methylation feature patterns at cell-type marker genes and computational integration of cells derived from different age groups^22^ (Fig. 1f and Extended Data Fig. 1r). Cell-type classifications based on DNA methylation and chromatin conformation were largely concordant (Extended Data Fig. 2a–c), with DNA methylation profiles providing a greater resolution for cell-type classification^3^ (Extended Data Fig. 2d). However, we found a notable exception in mid-gestational brains, in which a single neural progenitor radial glia (RG) population defined by DNA methylation signatures can be further discretely divided using chromatin conformation signatures (Extended Data Fig. 2e). Using chromatin conformation, we grouped RG cells into a neurogenic (RG-1) population and a putative astrocyte progenitor (RG-2) population^23^ (Extended Data Fig. 2e). This result was validated by an iterative classification of cells from mid-gestational brains, which found the gliogenic RG-2 population to be more discretely defined by chromatin conformation than by DNA methylation (Extended Data Fig. 2f).

The marked increase of non-CG methylation in neuronal cells, compared to moderate elevations of CG methylation, is an epigenomic hallmark of neuronal maturation^12^. The accumulation of non-CG methylation begins earlier in HPC excitatory and inhibitory neurons than in PFC neurons (Fig. 1g,h), with HPC cornu ammonis (CA) and inhibitory neurons containing substantial amounts of non-CG methylation (>1% mean non-CG methylation level) at gestational week (GW) 39. By contrast, comparable non-CG methylation levels were not observed in PFC neurons until the infant stage (4 and 7 months; Fig. 1g). The finding that the remodeling of CG and non-CG methylation in HPC precede those in PFC was further supported by genome-wide and gene-specific analyses and using additional GW-39 donors (Supplementary Note 2).

## Temporal order of multi-omic dynamics

**Fig. 2:**
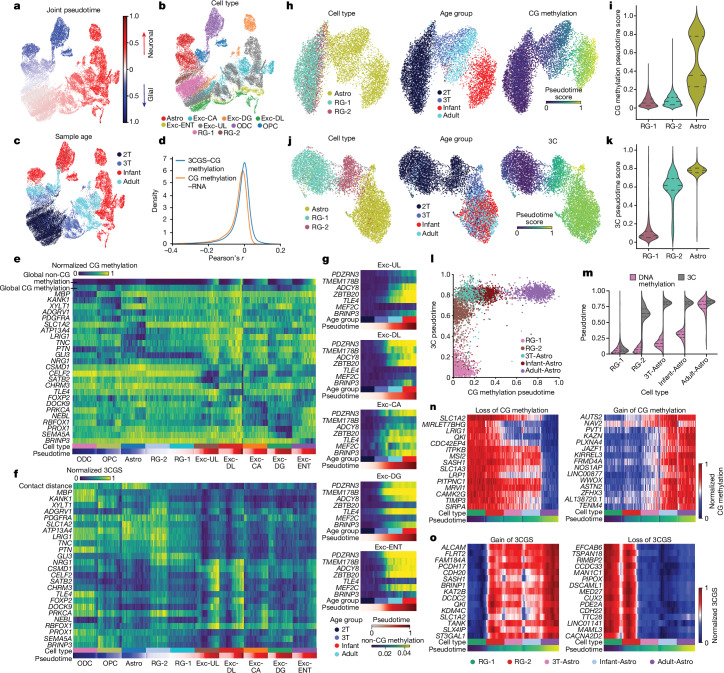
Temporal order of DNA methylation and chromatin conformation reconfiguration during the differentiation of astrocytes. **a**–**c**, UMAP dimensionality reduction of cortical RG-derived neuronal and glial cells. The UMAP is labelled by joint pseudotime scores computed using the fusion of DNA methylation and chromatin confirmation information (**a**), cell types (**b**) and sample ages (**c**). **d**, Distribution of Pearson’s coefficients for CG methylation–RNA and 3CGS–CG methylation correlations. **e**,**f**, Normalized gene body CG methylation (**e**) and 3CGS (**f**) for cell-type-specific marker genes. **g**, Absence of non-CG methylation accumulation at cell-type marker genes. **h**,**i**, Dimensionality reduction and pseudotime scores computed from CG methylation in neural progenitor RG-1, astrocyte progenitor RG-2 and astrocyte populations. **j**,**k**, Dimensionality reduction and pseudotime scores computed from chromatin conformation in RG-1, RG-2 and astrocyte populations. **l**, Distinct distributions of pseudotime scores computed from CG methylation or chromatin conformation during astrocyte differentiation. **m**, Direct comparison of pseudotime scores computed from CG methylation or chromatin conformation in individual cells across astrocyte differentiation, labelled by cell types. **n**,**o**, Examples of genes showing CG methylation (**n**) and 3CGS (**o**) dynamics during the differentiation of astrocytes.

Developing brain tissue consists of diverse cell populations at various stages of differentiation, making it challenging to analyse cell dynamics solely on the basis of the donor ages. We used pseudotime analysis to explore the temporal dynamics of chromatin conformation and DNA methylation by a more continuous time quantification^24^. Pseudotime scores were computed for cortical RG-derived cell populations using the fusion of CG methylation and chromatin conformation modalities (Fig. 2a–c). Pseudotime scores were computed separately for the glial trajectory in which RG differentiates into astrocytes, oligodendrocyte progenitor cells and oligodendrocytes, and the neuronal trajectory in which RG produces excitatory neurons (Fig. 2a–c). To quantify chromatin conformation at individual loci, we devised the 3C gene score (3CGS) representing the sum of intragenic chromatin contact frequency, which predominantly shows a negative correlation with gene body CG methylation (Fig. 2d and Supplementary Note 3). The pseudotime approach allows us to explore marker genes showing CG methylation dynamics throughout the continuous neurogenesis and gliogenesis processes (Fig. 2e,f), with CG methylation depletion or elevated 3CGS indicative of gene activation (Supplementary Note 4). As found in previous studies^12,25^, cell-type marker genes are commonly depleted of gene body non-CG methylation in neurons in the adult brain. This dataset further showed that whereas most genes gain non-CG methylation during neuronal maturation, cell-type marker genes are specifically protected from mCH accumulation (Fig. 2g).

We focused on the RG to astrocyte differentiation trajectory to investigate the observed discrepancy between DNA methylation and chromatin conformation regarding the separation of astrocyte progenitor RG-2 from the neural progenitor RG-1 clusters (Extended Data Fig. 2e). The cross-modality comparison revealed little CG methylation dynamics in RG-1 and RG-2 populations (Fig. 2h,i), whereas the reconfiguration of chromatin interactions was more continuous across the differentiation of RG-1 to RG-2 to early astrocytes (Fig. 2j,k). This resulted in a markedly different distribution of pseudotime scores computed from CG methylation or chromatin interactions in RG-2 and differentiated astrocytes in late-gestational and infant brains (Fig. 2l). The differentiation of RG to astrocytes can be divided into a stage of rapid chromatin conformation remodelling in RG-1 and RG-2 that predominantly occurs during mid-gestation, followed by a notably protracted maturation of the CG methylome that extends into the adult brain (Fig. 2l,m). Consistent with genome-wide pseudotime patterns, the findings of the gene-specific analyses showed that the remodelling of the 3CGS generally occurs in RG-1 and RG-2 populations and precedes CG methylation dynamics in differentiated astrocytes (Fig. 2n–o). By extending the cross-modality pseudotime analysis to other cell types, we found notable temporal separations of CG methylation and chromatin interaction dynamics in most cell-type differentiation trajectories with nuanced cell-type-specific patterns (Supplementary Note 5).

## In situ validation of cell-type markers

**Extended Data Fig. 3:**
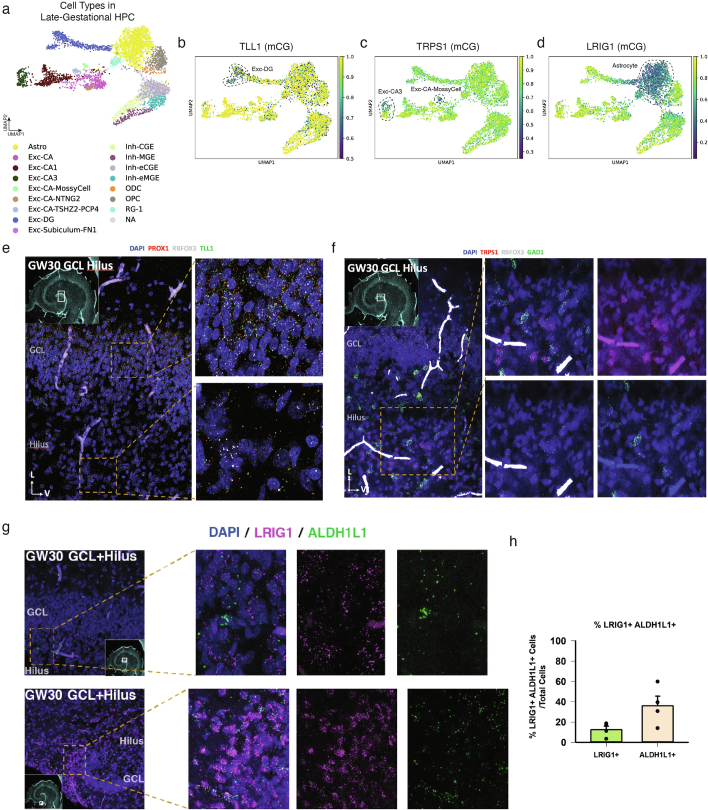
In situ validation of cell-type marker genes predicted by CG methylation patterns. (a) UMAP of brain cells derived from late-gestational HPC samples. (b) UMAP showing TLL1 CG hypomethylation for more matured granule neurons. (c) UMAP showing TRPS1 CG hypomethylation in Mossy Cells and partially in CA3 neurons. (d) UMAP showing LRIG1 hypomethylation in astrocytes. (e) single molecular RNA in situ detection of TLL1, PROX1, and RBFOX3 transcripts in the hippocampus in the third trimester (GW 30 GW). (f) single molecular RNA in situ detection of TPRS1, GAD1, and RBFOX3 transcripts in the hippocampus in the third trimester. (g) single molecular RNA in situ detection of LRIG1 and ALDH1L1 transcripts in the hippocampus in the third trimester. (h) Quantification of LRIG1/ALDH1L1 co-expression (N = 4 imaging fields of view).

In the adult human brain, gene body CG and non-CG methylation are predictive of cell-type-specific gene expression^12,20,25,26^. Here we extended the approach to the developing brain taking advantage of the inverse correlation between gene expression and CG methylation^20^ (Extended Data Fig. 3a–d). We used single-molecule fluorescence in situ hybridization to investigate the RNA expression patterns of cell-type markers identified by the methylation analyses. *TLL1,* a gene that shows reduced gene body CG methylation in granule cell layer neurons, was localized to the granule cell layer in the HPC in the third trimester (GW 30; Extended Data Fig. 3b,e). There were overlaps with RBFOX3, a molecular marker for mature neurons, and PROX1, a transcription factor (TF) found in granule neurons of the HPC (Extended Data Fig. 3e). *TRPS1* mRNA was expressed in excitatory (GAD1^−^) cells in the hilus and CA3 regions, supporting its expression in mossy cells and CA3 pyramidal neurons in the third trimester (Extended Data Fig. 3c,f). Last, we identified a reduced level of CG methylation in astrocytes at the *LRIG1* locus (Fig. 2n and Extended Data Fig. 3d). We found a substantial fraction (40%) of cells expressing a canonical astrocyte marker, ALDH1L1, as well as LRIG1 (Extended Data Fig. 3g,h), supporting the dynamic expression of LRIG1 during astrocyte differentiation.

## Neuronal-specific chromatin conformation

**Fig. 3:**
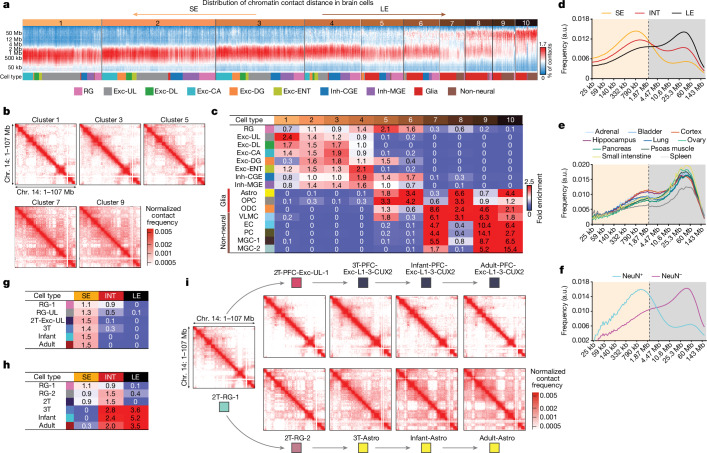
Remodelling of global chromatin conformation during human brain development. **a**, *k-*means clustering analysis groups single-cell 3C profiles by the distance distribution between interacting loci. **b**, Merged chromatin interaction profiles of the odd-numbered clusters identified in **a**. **c**, Cell-type-specific enrichments of clusters identified in **a**. EC, endothelial cell; PC, pericyte; VLMC, vascular leptomeningeal cell. **d**–**f**, Comparison of SE, LE and INT chromatin conformation found in single brain cells (**d**), bulk Hi-C profiles of diverse human tissues (**e**) and isolated neuronal and glial nuclei from primary adult human brain specimens (**f**). The vertical dashed lines indicate the threshold that separates short-range (coloured in orange) from long-range interactions (coloured in grey). a.u., arbitrary units. **g**,**h**, Remodelling of global chromatin conformation during the differentiation of upper-layer excitatory neurons (Exc-L1-3-CUX2) (**g**) and astrocytes (**h**) from the common RG-1 progenitor. **i**, Merged chromatin interaction profiles of developing cell populations across the differentiation of upper-layer excitatory neurons and astrocytes.

**Extended Data Fig. 4:**
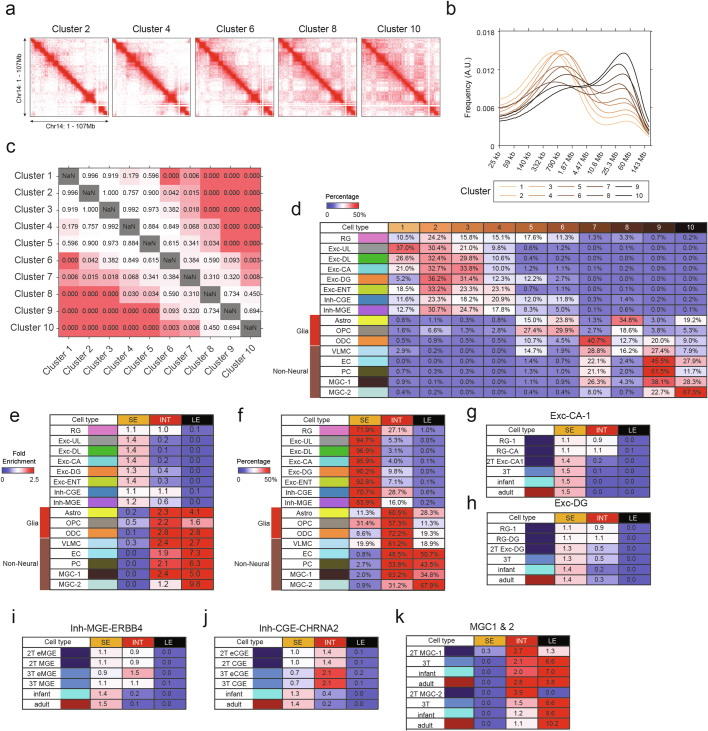
Remodeling of global chromatin conformation during human brain development. (a) Merged chromatin interaction profiles of even clusters identified in Fig. 3a. (b) Distribution of the distance between interaction loci of clusters identified in Fig. 3a. (c) Empirical p-values for the difference in distances of chromatin contact between pairs of clusters in Fig. 3a and Extended Data Fig. 4a. (d) Percentage of each brain cell type assigned to clusters identified in Fig. 3a. (e) Cell-type specific enrichments of SE (Short-range interaction Enriched), LE (Long-range interaction Enriched), and INT (Intermediate) chromatin conformation. (f) Percentage of each brain cell type classified as SE, LE, or INT conformation. (g-k) Remodeling of global chromatin conformation during the differentiation of Exc-CA-1 (g), Exc-DG (h), Inh-MGE-ERBB4 (i), Inh-CGE-CHRNA2 (j), and MGC-1 & MGE-2 (k).

Chromatin conformation capture techniques produce snapshots of three-dimensional (3D) genome architecture at multiple scales, including A and B compartments and more local features such as chromatin domains and loops^27^. Whereas A and B compartments are detected through long-range interactions (for example, >10 Mb distance), chromatin domains and loops are primarily detected by short-range interaction with less than 2 Mb distance. We clustered single-cell 3C profiles by the distribution of the distance between interacting loci using *k*-means clustering and found that single brain cells range from mainly containing short-range interactions (clusters 1–5) to containing a substantial amount of long-range interactions (clusters 6–10; Fig. 3a,b and Extended Data Fig. 4a,b). The distributions of chromatin contact distance are significantly different in the two types of cluster (clusters 1–5 versus 6–10; Extended Data Fig. 4c). Strikingly, neuronal cell types are strongly enriched in clusters 1–6, dominated by short-range interactions, whereas glial and non-neural cell types are enriched in clusters 8–10 and are dominated by long-range interactions (Fig. 3c and Extended Data Fig. 4d). We have developed thresholds to categorize the global chromatin conformation of each single cell into short-range interaction enriched (SE), long-range interaction enriched (LE) and intermediate (INT; Fig. 3d and Extended Data Fig. 4e,f). We analysed a published bulk Hi-C dataset generated from primary human tissues and found that bulk chromatin conformation profiles from all ten tissues show an LE signature^28^, suggesting that SE is specific to neuronal cells (Fig. 3e). Although Hi-C profiles generated from bulk human cortical and hippocampal tissues show a greater fraction of short-range interactions than other somatic tissues, they were nevertheless classified as LE-type samples probably owing to the abundant non-neuronal cells in the analysed tissue (Fig. 3e). The neuronal-specific SE conformation is supported by reanalysing bulk Hi-C profiles generated from neuronal and non-neuronal nuclei isolated from adult human brain PFC^29^ (Fig. 3f). The differentiation of neurons and astrocytes involved distinct global chromatin conformation remodelling events (Fig. 3g–i and Extended Data Fig. 4g–k). The neural progenitor RG-1 population is depleted of the LE conformation but is not enriched in either the SE or INT conformation (Fig. 3g,h). The chromatin conformation was rapidly remodelled in progenitors committed to producing upper-layer excitatory neurons (RG-UL) in the mid-gestational brain (2T-Exc-UL) and showed a comparable enrichment in the SE conformation to adult neurons (Fig. 3g,i). The differentiation of astrocytes involved a transition to the LE conformation, which was completed during late gestation (Fig. 3h,i).

## Multimodal chromatin and RNA imaging

**Fig. 4:**
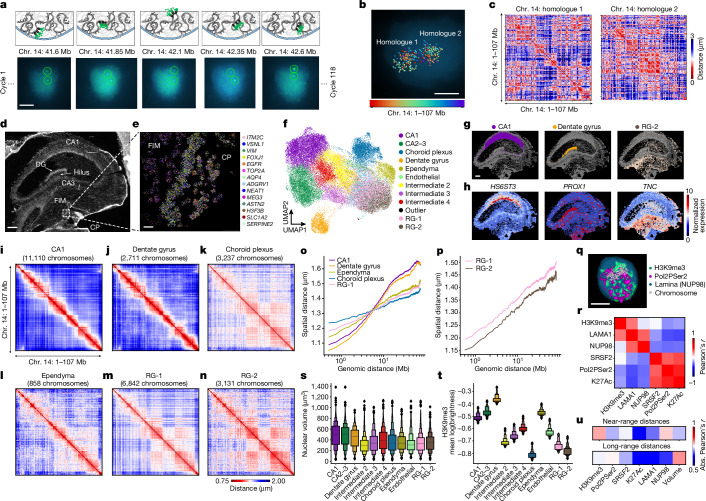
Multimodal imaging reveals SE chromatin conformation in newly differentiated hippocampal neurons. **a**, Sequential imaging of 354 genomic regions on the median-sized chromosome 14 using 119 rounds of hybridization with three-colour imaging. **b**, 3D localization of each genomic region in a single nucleus. **c**, Reconstruction of single-molecule chromatin conformation for two chromosome 14 homologues in a single nucleus. **d**, Overview of the tissue section containing HPC and choroid plexus structures. FIM, fimbria; CP, choroid plexus. **e**, Example of multiplexed RNA imaging using MERFISH. **f**, UMAP dimensionality reduction of the RNA MERFISH profile and cell-type annotation. **g**, Spatial localization of annotated cell types. **h**, Spatial expression patterns of marker genes for cell types shown in **g**. **i**–**n**, Reconstruction of chromatin conformation for CA1 (**i**), dentate gyrus (**j**), excitatory neuron, choroid plexus cell types (**k**), ependymal cells (**l**), RG-1 (**m**) and RG-2 (**n**). **o**,**p**, Quantification of spatial distance in micrometres as a function of genomic distance in megabases for differentiated brain cell types (**o**) and RG progenitor cells (**p**). **q**, Imaging of nuclear architectural proteins and histone modifications. **r**, Correlation of active and repression protein markers across genomic loci on chromosome 14. **s**,**t**, Quantification of nuclear volume (**s**) and mean H3 K9 trimethylation intensity (**t**) on chromosome 14 in distinct cell types. *n* = 24,099 imaged cells. The centre of the box plot marks the median, with each box above or below the median representing 10 percentiles of the data distribution. **u**, Correlation of the spatial distance for loci with near-range and long-range genomic distance with nuclear volume or mean intensities for protein markers on chromosome 14. Abs., absolute. Scale bars, 5 μm (**a**,**b**,**q**), 250 μm (**d**,**g**), 20 μm (**e**).

Quantifying the physical distance between chromatin loci using imaging is an orthogonal method that complements the proximity ligation and sequencing-based Hi-C approach. We sought to validate the neuronal-specific SE chromatin conformation in newly differentiated neurons in mid-gestational (GW 23) human brain tissue by jointly imaging the 3D organization, gene expression and nuclear architectural proteins using the chromatin tracing and RNA multiplexed error-robust fluorescence in situ hybridization (MERFISH) platforms^30–32^. Specifically, the median-sized chromosome 14 was imaged at a resolution of 250 kb by sequentially labelling 354 genomic loci uniformly covering the chromosome (Fig. 4a and Supplementary Table 4), allowing the conformation reconstruction of 46,023 homologues of chromosome 14 in 24,099 cells across HPC, fimbria and choroid plexus structures (Fig. 4b,c). RNA MERFISH was carried out on the same tissue section using a probe panel targeting 298 genes with cell-type-specific expression in the developing HPC^33^ (Fig. 4d,e and Supplementary Table 5). Brain cell types were identified by unbiased clustering of the MERFISH profiles followed by integration with the snm3C-seq3 DNA methylome using the *k*-nearest neighbours approach (Fig. 4f–h and Supplementary Note 6).

**Extended Data Fig. 5:**
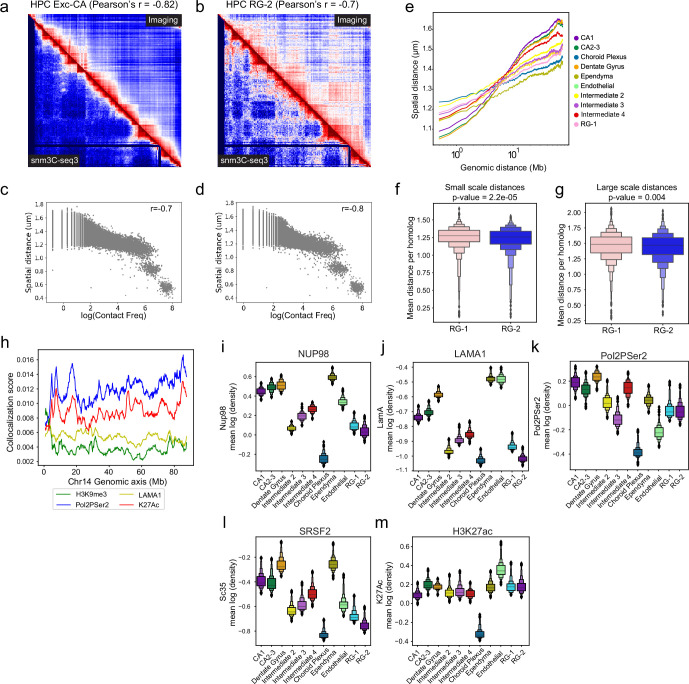
Chromatin- and RNA-MERFISH analysis of mid-gestational human brain development. (a-b) Comparison of contact matrices derived from imaging (lower left) and snm3C-seq3 (upper right) for HPC-Exc-CA (a) and HPC-RG-2 (b). (c-d) Correlation of snm3C-seq3 contact frequency to spatial distances quantified with imaging for HPC-Exc-CA (c) and HPC-RG-2 (d). (e) Quantification of spatial distance in µm as a function of genomic distance in Mb for brain cell types. (f-g) Comparison of the spatial distances of short-range interactions (f) and long-range interactions (g) between RG-1 and RG-2. (h) Quantification of protein marker density across the length of chromosome 14. (i-m) Median log (density) of NUP98 (i), LAMA1 (j), Pol2ser2 (k), SRSF2 (l), and H3K27ac (l) on chromosome 14.

Average cell-type-specific distance matrices reconstructed from imaging chromosome 14 recapitulated key features of the chromosomal organization, including topologically associating domains and compartmental structures observed in Hi-C contact matrices (Extended Data Fig. 5a–d). Imaging quantification of spatial distance between genomic loci revealed striking differences between neurons and non-neuronal cells. In neuronal cell types such as CA1 or dentate gyrus excitatory neurons, genomic regions separated by short genomic distances (up to 5 Mb) showed a compact spatial distance, which is indicative of a high interaction frequency (Fig. 4i,j,o and Extended Data Fig. 5e). By contrast, distal genomic regions showed larger physical distances in neuronal cell types indicating low interaction frequencies (Fig. 4i,j,o). An opposite pattern was observed in progenitors and non-neuronal cell types, for which loci separated by short genomic distances show an increased physical distance, whereas distal genomic loci exhibit reduced spatial distances, compared to that observed in neuronal cell types (Fig. 4k–o and Extended Data Fig. 5e). The imaging results are consistent with the enrichment of SE and LE conformations in neurons and non-neuronal cells, respectively. Furthermore, the imaging results validated the emergence of the SE conformation (increased short-range interaction and decreased long-range interaction) during the differentiation of RG to excitatory neurons. A direct comparison of RG-1 and RG-2 has found a more compact configuration of chromosome 14 across the whole range of genomic distances in RG-2 (*P* value for near-range genomic distances: 2.2 × 10^−5^; *P* value for long-range distance: 4 × 10^−3^; two-sided rank-sum test; Fig. 4m,n,p and Extended Data Fig. 5f,g).

Recent studies have suggested that nuclear volume variation could affect cell-type-specific chromatin conformation^18^. The large nuclear size of neurons could decrease the probability of long-range chromatin interaction and lead to an SE conformation. Through imaging a set of nuclear architectural proteins and post-translational modifications (Fig. 4q–u), we found that the spatial distances of distal regions were indeed most strongly correlated with nuclear volume (Fig. 4u), whereas the spatial distances of genomic loci separated by short genomic distances were best correlated with H3 K9 trimethylation (Supplementary Note 7). Together, the findings of our imaging analysis of the mid-gestational HPC demonstrated spatially distinct chromatin conformation signatures that marked transitions from neural progenitors to mature neurons.

## Chromatin compartmental remodelling

**Extended Data Fig. 6:**
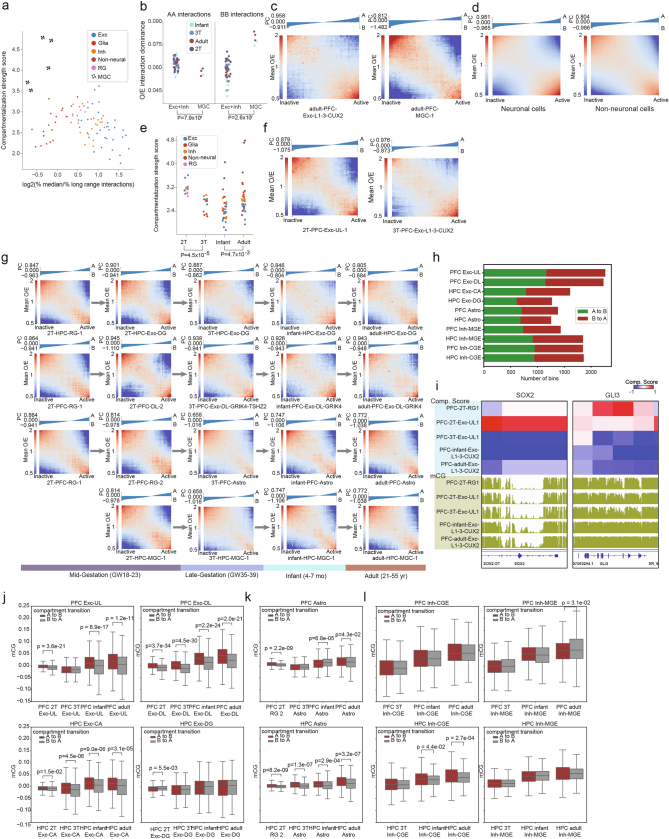
Chromatin compartment dynamics across cell types and developmental stages. (a) Relationship between compartmentalization and the dominance of median vs. long-range interactions across cell types. (b-c) Differing dominance of chromatin interactions in the active A compartment or the inactive B compartment between neuronal (Exc+Inh) and microglia (MGC) populations. (d) Comparison of chromatin compartment strength between neuronal and non-neuronal cells using Hi-C data (Hu et al., 2021) generated from isolated nuclei 30. (e-f) Decrease in compartment strength from 2T to 3T. Saddle plots are shown in (f) to quantify the interaction inside or between the active A compartment and the inactive B compartment. (g) A/B compartment saddle-plots for 4 cell trajectories, showing a general decrease in compartmentalization from mid-gestation to late-gestation, as well as the dominance of interactions in the inactive B compartment in HPC-MGC-1 (bottom row). (h) Number of genomic regions switching from A to B compartment or from B to A compartment in each cell-type development trajectory. (i) Compartmental and CG methylation dynamics at SOX2 and GLI3 loci during the differentiation of cortical upper layer excitatory neurons. (j-l) Correlation between developmental changes of CG methylation and compartmental dynamics. Changes in CG methylation compared to the earliest developmental stage were shown for (j) excitatory neurons, (k) astrocytes, and (l) inhibitory neurons.

In exploring the chromatin compartmental diversity across cell types and developmental stages, we found that cell types showing the LE conformation are associated with a stronger compartment strength: compartmentalization strength scores are inversely correlated with the ratio of short- to long-range interactions (Pearson’s *r* = −0.35, *P* = 8.1 × 10^−4^; Extended Data Fig. 6a). For example, microglia populations show the strongest LE conformation and strongest compartment strengths (Extended Data Fig. 6a). The compartment strength of microglia is primarily contributed by interactions in the inactive B compartment, whereas the compartment strength of neuronal populations is more strongly contributed by the active A compartment (Extended Data Fig. 6b,c). The enrichment of A compartment strength in neuronal cells was validated using published bulk Hi-C profiles of purified neuronal and non-neuronal nuclei^29^ (Extended Data Fig. 6d). In addition, compartment strength is developmentally regulated, which includes a substantial loss of compartmentalization strength score between mid-gestation and late gestation followed by a gradual gain during further development (Extended Data Fig. 6e–g). We further analysed genomic regions associated with developmentally differential compartments and found regions switching from the A to the B compartment accumulated a greater amount of CG methylation, supporting the notion that DNA methylation reinforces inactive chromatin compartments^34,35^ (Supplementary Note 8).

## Dynamics in chromatin loops and domains

**Extended Data Fig. 7:**
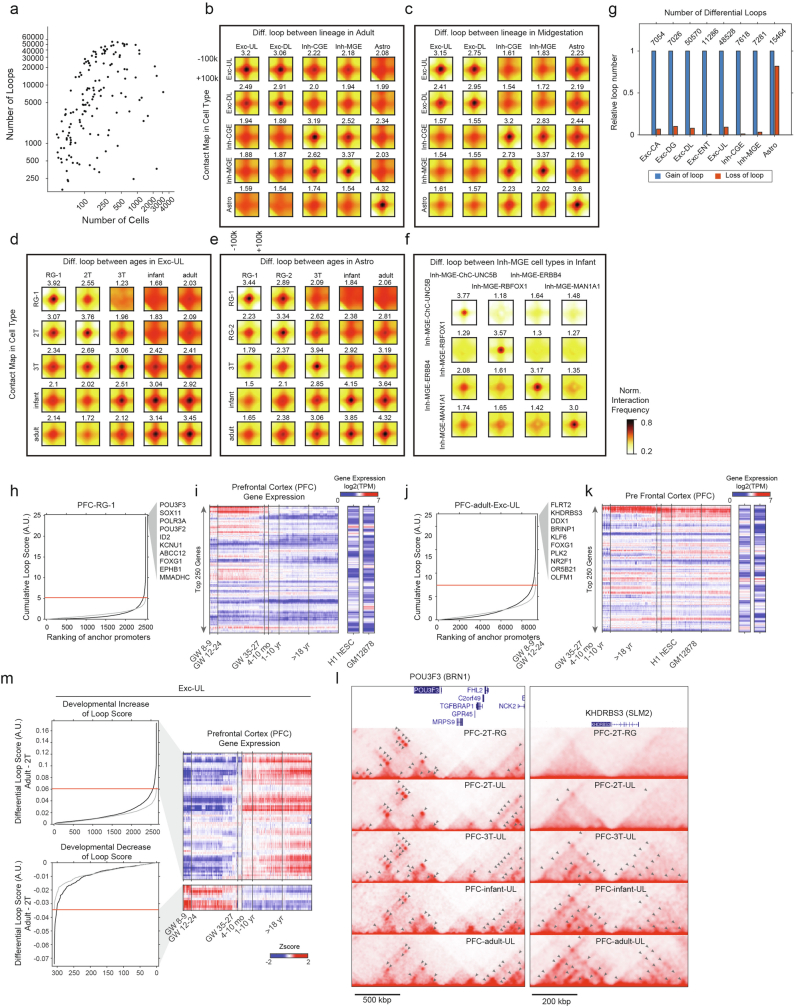
Chromatin loop dynamic across cell types and developmental stages. (a) Correlation between the number of cells in each cell population and the number of chromatin loops identified. (b-f) Aggregated chromatin contact signals at differential loops identified across cell types in the adult brain (b), cell types in the mid-gestational brain (c), developmental stages for the Exc-UL trajectory (d), developmental stages for the astrocyte trajectory (e), and subtypes of MGE derived inhibitory neurons in the infant brain (f). The value above each aggregated profile indicates the APA score to evaluate the enrichment of identified loops with respect to the lower left background. (g) Numbers of gain or lost loops across the trajectories of cell type differentiation. (h) Identification of SIPs in PFC-2T-RG-1. (i) Gene expression patterns of the top 250 genes whose promoter is associated with the highest cumulative loop scores in PFC-2T-RG-1. (j) Identification of SIPs in PFC-adult-Exc-UL. (k) Gene expression pattern of top 250 genes whose promoter associated with the highest cumulative loop scores in PFC-adult-Exc-UL. (l) Chromatin loops dynamics at POU3F3 (BRN1) and KHDRBS3 (SLM2) loci across developmental stages. (m) Identification of dev-SIPs associated with the differentiation of Exc-UL. Gene expression patterns of dev-SIPs showing either developmental increase or decrease of loop scores were shown on the left.

**Extended Data Fig. 8:**
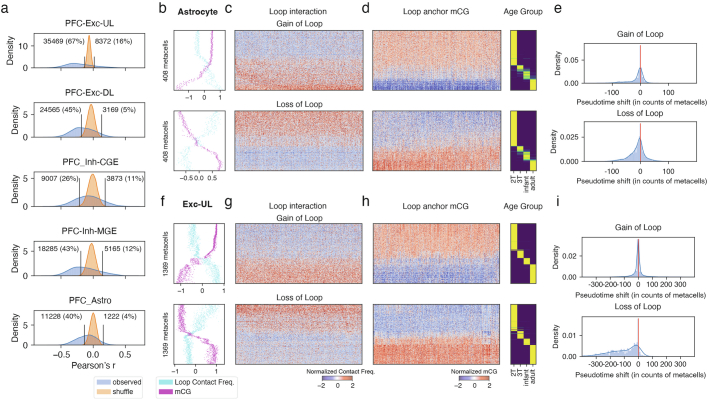
Correlation between chromatin loop dynamics and CG methylation level of the loop anchor regions. (a) Distribution of the Pearson’s correlation coefficients between loop strength and CG methylation of loop anchor regions for developmentally differential loops. (b-e) Correlation between loop strength and CG methylation of loop anchor regions across astrocyte differentiation. (b) Normalized loop contact frequencies (cyan) and CG methylation levels of anchor regions (magenta) across meta-cells ranked by developmental pseudotime scores. (c-d) Normalized values of loop interaction frequency (c) and CG methylation of loop anchor regions (d). (e) Quantification of the lag of CG methylation remodeling by the amount of pseudotime shife (in counts of meta-cells) required to maximize the inverse correlation between loop strength and CG methylation levels of loop anchor regions. (f-i) Correlation between loop strength and CG methylation of loop anchor regions across the differentiation of cortical upper layer excitatory neurons.

**Extended Data Fig. 9:**
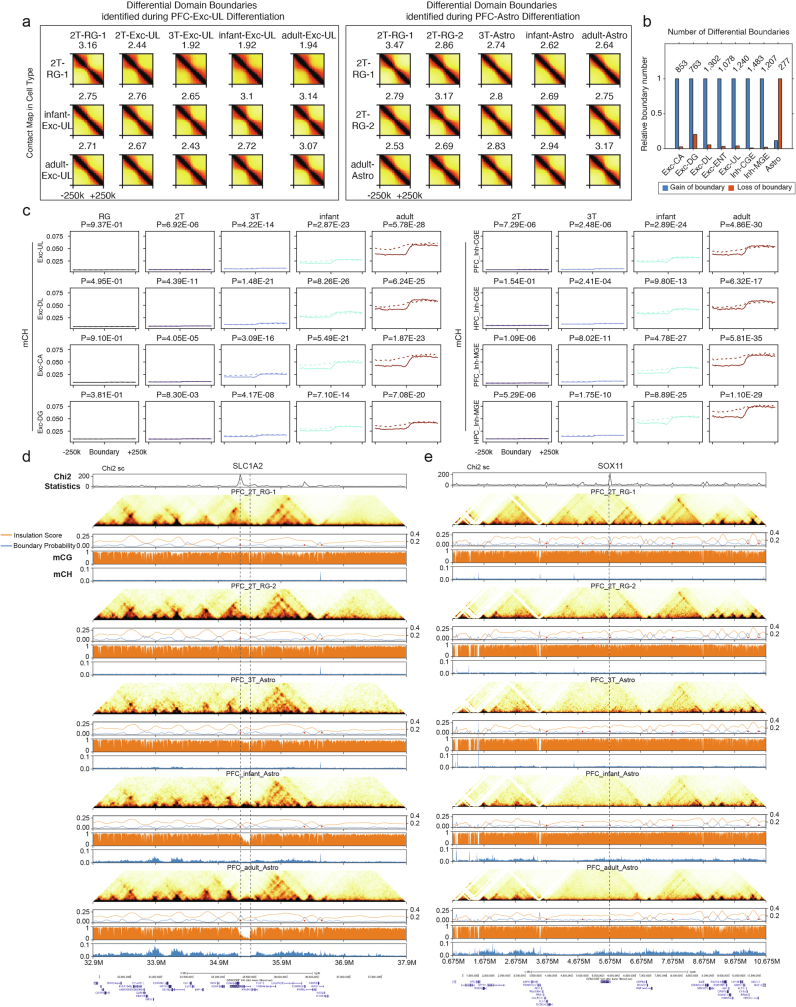
Cell-type and developmental dynamics of chromatin domain boundaries. (a) Aggregated chromatin contact signals at differential chromatin domain boundaries identified in the differentiation of Exc-UL and Astro. The value above each aggregated plot indicates 1/(insulation score), so a greater score indicates stronger insulation. (b) Numbers of gain or lost domain boundaries across the trajectories of cell type differentiation. (c) non-CG methylation level at domain boundaries identified for each cell type trajectory. Analysis of true boundaries (solid lines) shuffled (dashed lines). (d-e) Differential chromatin domain boundaries were identified during the differentiation of astrocytes at SLC1A2 (d) and SOX11 (e) loci.

We identified chromatin loops using scHiCluster, optimized for single-cell Hi-C profiles^19^. The number of identified chromatin loops is positively correlated with the cell-type abundance (Spearman’s *r* = 0.47, *P* < 2 × 10^−22^; Extended Data Fig. 7a and Supplementary Table 6). We further used an approach based on analysis of variance to identify differential chromatin loops across the differentiation of cell-type trajectories (Extended Data Fig. 7b–f and Supplementary Table 6). In all neuronal trajectories, developmentally gained loops outnumber developmentally lost loops by more than tenfold (Extended Data Fig. 7g), which is consistent with the strengthening of the SE conformation in neuronal cell types. By contrast, similar numbers of gained and lost loops were found during the differentiation of astrocytes that show an LE conformation (Extended Data Fig. 7g). Promoters that are highly connected through chromatin interactions or super-interactive promoters (SIPs) have previously been found to be enriched in lineage-specific genes^16^. Here we extended the SIP analysis to multiple development stages and found that prenatal development and postnatal development were associated with different SIPs (Extended Data Fig. 7h–k and Supplementary Table 6), reflecting the distinct biological processes associated with each stage. SIP-associated genes (for example, *POU3F3*) found in RG cells are enriched in cell proliferation and cortical cell migration functions, whereas SIP-associated genes (for example, *KHDRMS3*, also known as *SLM2*) found in upper-layer excitatory neurons in infant brains are enriched in terms including transmitter-gated channel activity and synapse (Extended Data Fig. 7h,j,l). We further identified promoters that are associated with high cumulative scores of differential loops (Extended Data Fig. 7m and Supplementary Table 6). Using the BRAINSPAN developmental transcriptome resource^11^, we found a strong correlation between the developmental decrease of the cumulative loop score and gene repression and between an increase of the cumulative loop score and gene activation (Extended Data Fig. 7m). Expanding on previous studies that correlated loop strength with CG methylation in the adult human brain^3,19^, we found that differential loops across each cell-type trajectory predominantly show an inverse correlation with the CG methylation level of loop anchor regions (Extended Data Fig. 8 and Supplementary Note 9). Last, the analysis of differential chromatin domain boundaries has recapitulated the impact of the SE and LE conformations on local chromatin structures: the strengthening of the SE conformation during neuronal differentiation led to more gained domain boundaries than losses of boundaries in neuronal trajectories, whereas the formation of the LE configuration was associated with more loss of boundaries than gains of boundaries (Extended Data Fig. 9 and Supplementary Note 10).

## Regulatory programs of development

**Fig. 5:**
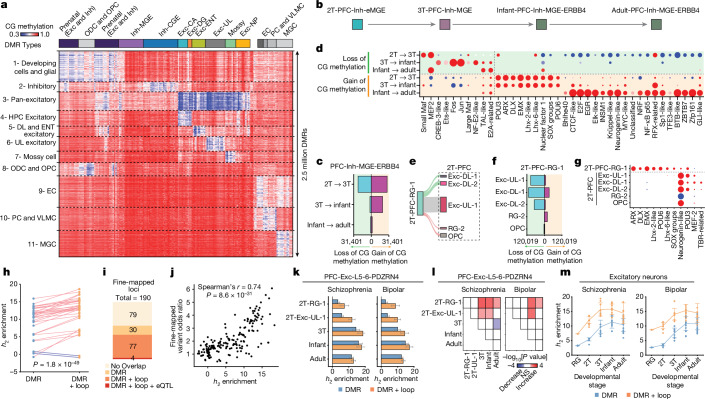
Localizing the heritability signals of neuropsychiatric disorders using DMRs and chromatin loops. **a**, *k*-means clustering of DMRs reveals specificities for cell lineages and developmental stages. **b**, Schematic of the maturation of MGE-derived ERBB4-expressing inhibitory neurons (Inh-MGE-ERBB4). **c**, Numbers of trajectory-DMRs identified for Inh-MGE-ERBB4 maturation between adjacent developmental stages. **d**, Enriched TF-binding motifs in trajectory-DMRs for the maturation of Inh-MGE-ERBB4 neurons. The enrichment of TF-binding motif is determined using hypergeometric tests with a *q* value threshold of 0.01 for display. **e**, Schematic of the specification of RG-1-derived cell types. **f**, Numbers of branch-DMRs found during RG-1 differentiation. **g**, TF-binding motif enrichments in branch-DMRs associated with RG-1 differentiation. **h**, The enrichment of schizophrenia polygenic heritability in DMRs and loop-connected DMRs. The *P* value was computed using a two-sided paired *t*-test. **i**, Numbers of schizophrenia-associated loci containing at least one fine-mapped variant that overlaps with DMRs or loop-connected DMRs. **j**, Spearman’s correlation and two-sided *P* value between the enrichment of polygenic heritability and fine-mapped schizophrenia variants. **k**, Enrichment of polygenic heritability for schizophrenia and bipolar disorder in PDZRN4-expressing layer 5–6 excitatory neurons across developmental stages. Error bars indicate standard errors estimated by the linkage disequilibrium score regression block jackknife method (*n* = 200 blocks). **l**, Statistical significance of differential heritability enrichment between development stages. *P* values were computed using two-sided *t*-tests. Red and blue colours show developmentally increased or decreased heritability enrichment, respectively. NS, not significant. **m**, Meta-analysis of heritability enrichment for schizophrenia and bipolar disorder in excitatory neuron populations. Error bars indicate standard deviations across cell types included in the meta-analysis. *n* = 5 cell types for the second trimester, *n* = 9 for the third trimester, *n* = 16 for infant, *n* = 17 for adult.

DMRs of CG methylation are a reliable marker of dynamic regulatory activity, with a loss of methylation indicating an increase in regulatory activity and a gain of methylation associated with repression^36,37^. We investigated the global regulatory dynamics of human cortical and hippocampal development by identifying more than 2.5 million DMRs across all cell types and developmental stages (Fig. 5a and Supplementary Table 6), followed by the analysis of TF-binding motif enrichment (Supplementary Note 11). The developmental dataset generated in this study allows us to infer the temporal sequence of TF activity. We have identified dynamic DMRs across the stages of cell-type specification (trajectory-DMRs; Fig. 5b–d, Supplementary Note 12, Supplementary Figs. 7 and 8 and Supplementary Table 6) and DMRs that distinguish daughter cell populations derived from a common mother cell type (branch-DMRs; Fig. 5e–g, Supplementary Figs. 9 and 10 and Supplementary Table 6). Using TF-binding motif analysis, we found that the regulatory landscape of both excitatory and inhibitory neurons is shaped by the sequential action of lineage-specific and activity-dependent TFs. Regulatory elements that become activated (loss of CG methylation) in mid-gestation are enriched in the binding motifs of lineage-specific TFs such as Maf and MEF2 for inhibitory cells or neurogenin, MEF2 and POU3 for excitatory neurons (Fig. 5d and Supplementary Fig. 7). Following lineage specification, the binding motif of activity-dependent TFs (FOS, JUN, EGR1 and CREB) is strongly enriched in regulatory elements activated in late-gestation to infant stages in both excitatory and inhibitory populations^38^ (Fig. 5d). This result suggests late-gestational to early-infant development as a key stage during which the epigenome is shaped by neuronal activity. The analysis of branch-DMRs associated with RG-2 differentiation supported the gliogenic characteristic of this progenitor pool as the binding motif of neurogenic TFs is strongly depleted in regions losing CG methylation in RG-2 (Fig. 5e–g).

## Dissection of neuropsychiatric risk loci

**Extended Data Fig. 10:**
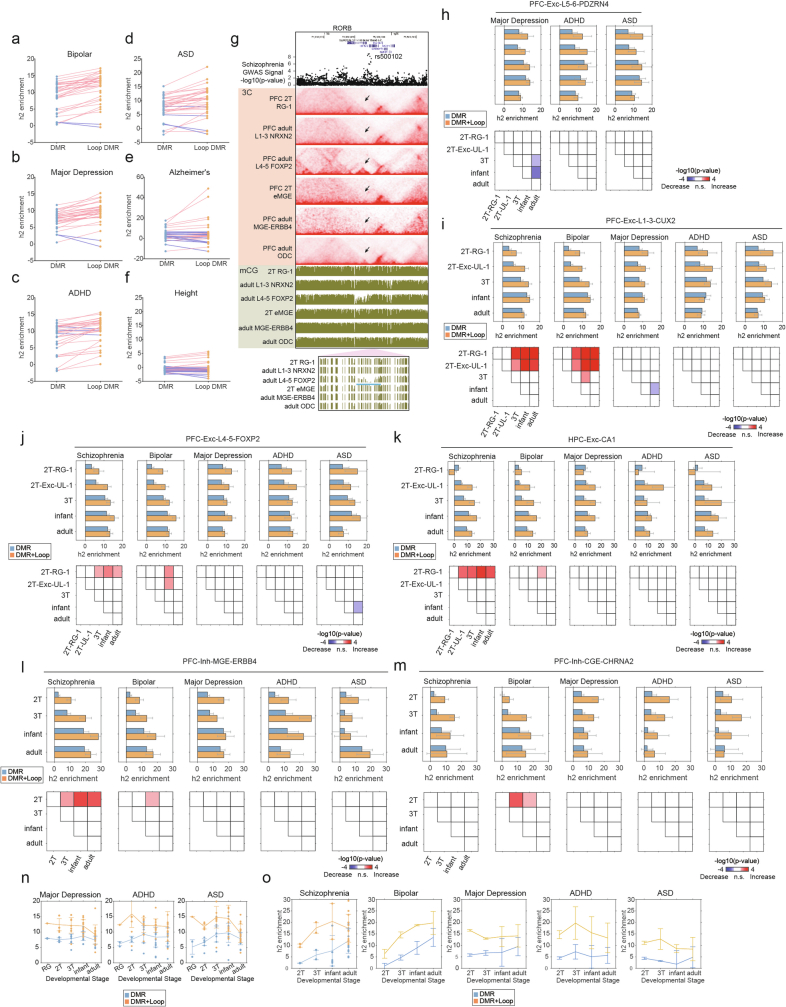
Heritability enrichment analysis of neuropsychiatric disorders. (a-b) The enrichment of polygenic heritability for bipolar disorder (a), major depression (b), ADHD (c), ASD (d), Alzheimer’s disease (e), and height (f) in DMRs and loop-connected DMRs. (g) The genomic region overlapping with a putative causal variant for schizophrenia rs500102 is connected to the RORB promoter through a cell-type-specific loop domain. (h-m) Enrichment of polygenic heritability for neuropsychiatric disorders across developmental stages in PFC-Exc-L5-6-PDZRN4 (h), PFC-Exc-L1-3-CUX2 (i), PFC-Exc-L4-5-FOXP2 (j), HPC-Exc-CA1 (k), PFC-Inh-MGE-ERBB4 (l), PFC-Inh-CGE-CHRNA2 (m). (n-o) Meta-analysis of heritability enrichment for neuropsychiatric disorders in excitatory (n) and inhibitory (o) neuron populations.

Using DMRs and chromatin loops identified in this study, we systematically localized the heritability signals of neuropsychiatric disorders across developmental stages and cell populations. The polygenic heritability enrichment of annotations defined by DMR and/or chromatin loops was quantified for each cell type using stratified linkage disequilibrium score regression^39^ (Supplementary Figs. 11 and 12). We found significantly greater enrichment of heritability in loop-connected DMRs than in all DMRs (Fig. 5h and Extended Data Fig. 10a–f; *P* = 1.8 × 10^−49^ through paired *t*-test), supporting the utility of chromatin loops in locating potential causal variants. We also overlapped fine-mapped putative causal loci of schizophrenia^40^ to DMRs and loop-connected DMRs (190 independent loci containing 569 high-confidence putative causal single nucleotide polymorphisms (SNPs) with posterior inclusion probability > 0.1; Supplementary Table 7). Out of 190 schizophrenia fine-mapped loci, 111 and 81 loci contain at least 1 putative causal SNP that overlaps with a DMR or loop-connected DMR, respectively (Fig. 5i). We found a strong correlation between the odds ratio of overlapping with a putative causal SNP and the enrichment of polygenic heritability across cell types (Fig. 5j; Spearman’s correlation = 0.74, *P* = 8.6 × 10^−31^). As an example, we showcase rs500102 (posterior inclusion probability = 0.27), a putative causal variant for schizophrenia that overlaps with a loop-connected DMR in L4–5 excitatory neurons (Extended Data Fig. 10g). The variant is also a fine-mapped expression quantitative trait locus of RORB detected in the brain tissue by Genotype-Tissue Expression studies^41^ (Supplementary Table 7). The region where rs500102 is localized is connected by a loop domain to the RORB promoter, specifically in L4–5 excitatory neurons (Extended Data Fig. 10g). The loop domain is associated with cell-type-specific reduction of CG methylation in the RORB gene body as well as in the region surrounding rs500102 (Extended Data Fig. 10g). This example demonstrates the utility of single-cell multi-omic profiles to generate mechanistic hypotheses regarding the function of variants associated with genome-wide association studies.

Next we assessed the developmental dynamics of enrichment for neuropsychiatric disorder heritability in various neuronal populations (Fig. 5k,l and Extended Data Fig. 10h–m). We observed similar patterns over developmental trajectories for DMRs and loop-connected DMRs while noting that loop-connected DMRs show higher overall heritability enrichment. For schizophrenia and bipolar disorder, the enrichment of polygenic heritability increases from neuroprogenitors (RG-1) to early post-mitotic neurons (for example, 2T-Exc-UL-1) and further to post-mitotic neurons in late-gestational brains for both excitatory (Fig. 5k,l and Extended Data Fig. 10h–k) and inhibitory (Extended Data Fig. 10l,m) populations. We also found a trend of decreased heritability enrichment in adult neurons for schizophrenia and bipolar disorder, although the decreases are not statistically significant except for in an L5–6 excitatory population (Fig. 5k,l). Using meta-analyses of all excitatory (Fig. 5m and Extended Data Fig. 10n) or inhibitory (Extended Data Fig. 10o) populations, we found a consistent developmental increase of enrichment for schizophrenia and bipolar disorder between neuroprogenitors and neurons in infant brains, followed by a decrease in the adult brain. Taken together, our results indicate that the genetic risk of schizophrenia and bipolar disorder more strongly affects post-mitotic neurons than the neuroprogenitor population in developing human brains.

## Discussion

Genome-wide rearrangements of the DNA methylome and chromatin conformation are crucial for the normal development of mammalian brains. Our study underscores the dynamic shifts from progenitors to neuronal and glial populations in the second and third trimesters to the neonatal period, highlighting the importance of using primary brain specimens in studies of perinatal development. This work provides a data resource to understand the genetic and epigenetic mechanisms of brain diseases. For example, the single-cell multi-omic dataset generated by snm3C-seq3 provides cell-type-specific functional annotations (that is, DMRs and chromatin loops) to more than half of the fine-mapped schizophrenia-associated loci, highlighting the application of snm3C-seq3 profiles in dissecting the developmental context and molecular mechanism of non-coding variants associated with neuropsychiatric disorders. The pervasive remodelling of the neuronal methylome and chromatin conformation during perinatal development suggests that the human brain is particularly vulnerable to genetic and environmental perturbations that affect these developmental stages. Consistent with this conjecture, the localization of the polygenic risk of schizophrenia and bipolar disorder suggests a peak of heritability enrichment during the third trimester and infancy.

Our study found the temporal separation of DNA methylation and chromatin conformation reconfigurations, suggesting the asynchrony across gene regulatory mechanisms (for example, TF binding, DNA methylation or histone modifications) might be common in dynamic biological systems such as human brain development. In addition to the exceptional abundance of non-CG methylation, we found another layer of unique epigenomic regulation in neurons (that is, the unusually strong enrichment of short-range chromatin interactions that is different from the case for glial cells or non-brain tissues). Using multimodal single-molecule imaging of chromatin, RNA and nuclear protein markers, we found that the neuronal-specific SE chromatin configuration is established during early development (for example, mid-gestation) and is correlated with the histone modification H3 K9 trimethylation, transcription and a change in the nuclear volume. This finding raises questions regarding whether cohesin-dependent enhancer–promoter loops are regulated differently in neurons than in non-neuronal cell types^42^, as well as the potential impact of the nuclear volume on chromatin folding and gene regulation.

## Methods

### Ethics statement

Paediatric tissues obtained from the University of California, San Francisco (UCSF) were collected from autopsy sources through the UCSF Pediatric Neuropathology Research Laboratory. Informed consent was obtained from the next of kin for all paediatric samples obtained from the Pediatric Neuropathology Research Laboratory. Paediatric samples collected through autopsy were de-identified before acquisition and thus exempt from Institutional Review Board (IRB) review. For tissues obtained through the gynaecology clinic, patients were asked about their interest in donating tissue to research after making the decision for termination of pregnancy. Patients who agreed signed a written consent after receiving information, both written and oral, given by a physician or midwife. They were informed that agreeing to donate would not affect their medical care and that neither the donor nor the clinical team would benefit from the donation. The use of abortion material was reviewed and approved by the UCSF Committee on Human Research. Protocols were approved by the Human Gamete, Embryo, and Stem Cell Research Committee (IRB GESCR number 10-02693; IRB number 20-31968) at UCSF.

Adult human brain samples and a post-mortem GW-35 sample (based on adjusted age) were banked by the National Institutes of Health (NIH) NeuroBioBank at the University of Maryland Brain and Tissue Bank (Supplementary Table 1). Informed consent was obtained from the patient or next of kin for all samples obtained from NIH NeuroBioBank. The tissue collection and repository is overseen by The University of Maryland IRB with IRB protocol number HM-HP-00042077, as well as The Maryland Department of Health and Mental Hygiene IRB with IRB protocol number 5-58. When an individual of any age dies, the medical examiner or coroner contacts the next of kin and asks whether they would be willing to talk to a staff member at the University of Maryland Brain and Tissue Bank about an NIH-funded tissue procurement project. If the family agrees, the medical examiner or coroner contacts the bank, and a staff member obtains a recorded telephone consent from the next of kin for donation. A written verification of the consent is then faxed to the office of the referring medical examiner or coroner. Alternatively, the recording can also be played over the telephone for confirmation. The University of California, Los Angeles IRB has determined that our study using post-mortem human tissue obtained from the NIH NeuroBioBank involves no human participant and requires no IRB review.

### Brain specimens

Collections were carried out at post-mortem intervals of less than 24 h. Tissue was collected through the UCSF, and the NIH NeuroBioBank. Patient consent was obtained before collection according to institutional ethical regulations. The UCSF Committee on Human Research reviewed protocols that were approved by the Human Gamete, Embryo and Stem Cell Research Committee (IRB GESCR number 10-02693) and associated IRBs (20-31968) at UCSF. For prenatal samples, mothers gave consent for the related medical procedure before any consent for donation of tissue. In the tissue consent process, they were informed that agreeing to donate would not affect their medical care and that neither the donor nor the clinical team would benefit from the donation. Specimens collected from UCSF were evaluated by a neuropathologist as control samples collected through the UCSF Pediatric Neuropathology Research Laboratory. Additional samples were identified from the NIH NeuroBioBank, according to their IRB approval. Tissues were cut coronally, and areas of interest were sampled. Tissue blocks of 1 mm used for the snm3C-seq3 assay were flash-frozen in liquid nitrogen and stored at −80 °C. Blocks used for histological analyses were fixed with 4% paraformaldehyde (PFA) for 2 days and cryoprotected in a 30% sucrose gradient. The tissue was then frozen in OCT, and blocks were cut at 30 µm with a cryostat and mounted onto glass slides. For each sample used, we cresyl-stained three sections spanning the block to ensure our position using anatomical landmarks, such as the lateral ventricle, presence of the caudate, thalamus and HPC.

### snm3C-seq3

For prenatal brain samples, snm3C-seq3 was carried out without the labelling of neuronal nuclei using anti-NeuN antibody, whereas postnatal samples were labelled by anti-NeuN antibody during the procedure to isolate nuclei. For snm3C-seq3 carried out without labelling, frozen powder of brain tissue was resuspended in 10 ml of DPBS with 2% formaldehyde and incubated at room temperature for 10 min with slow rotation. The crosslinking reaction was quenched with 1.17 ml of 2 M glycine for 5 min at room temperature. The crosslinked tissue sample was pelleted by centrifugation at 2,000*g* for 10 min at 4 °C. The same centrifugation condition was used to pellet nuclei throughout the snm3C-seq3 procedure. The pellet was resuspended in 3 ml NIBT (10 mM Tris-HCl pH 8.0, 0.25 M sucrose, 5 mM MgCl_2_, 25 mM KCl, 1 mM dithiothreitol, 0.1% Triton X-100 and 1:100 protease inhibitor cocktail (Sigma number P8340)). The resuspended tissue sample was dounced with a dounce homogenizer (Sigma number D9063) 40 times with a loose pestle and 40 times with a tight pestle. For snm3C-seq3 carried out with anti-NeuN labelling, anti-NeuN antibody (PE-conjugated, clone A60, Millipore-Sigma number FCMAB317PE) was added to NIBT at a 1:250 dilution and was incubated with the tissue lysate during the homogenization steps for a total of 15 min. The lysate was mixed with 2 ml of 50% iodixanol (prepared by mixing OptiPrep density gradient medium (Sigma number D1556) with diluent (120 mM Tris-Cl pH 8.0, 150 mM KCl and 30 mM MgCl_2_) with a volume ratio of 5:1). The lysate was gently layered on top of a 25% iodixanol cushion and centrifuged at 10,000*g* for 20 min at 4 °C using a swing rotor. The pellet of nuclei was resuspended in 1 ml of cold DPBS followed by quantification of nuclei using a Biorad TC20 Automated Cell Counter (Biorad number 1450102).

The in situ 3C reaction was carried out using an Arima Genomics Arima-HiC+ kit. Each in situ 3C reaction used 300,000 to 450,000 nuclei. Nuclei aliquots were pelleted and resuspended in 20 µl H_2_O mixed with 24 µl conditioning solution and incubated at 62 °C for 10 min. After the incubation, 20 µl of stop solution 2 was added to the reaction and incubated at 37 °C for 15 min. A restriction digestion mix containing 7 µl of 10× NEB CutSmart buffer (NEB number B7204), 4.5 µl of NlaIII (NEB number R0125), 4.5 µl of MboI (NEB number R0147) and 12 µl of 1× NEB CutSmart buffer was added to the reaction followed by incubation at 37 °C for 1 h. The restriction digestion reaction was stopped by incubation at 65 °C for 20 min. A ligation mix containing 70 µl of buffer C and 12 µl of enzyme C was added and then incubated at room temperature for 15 min. The reaction was then kept at 4 °C overnight.

Before fluorescence-activated nucleus sorting, 900 µl cold DPBS supplemented with 100 µl ultrapure BSA (50 mg ml^−1^, Invitrogen number AM2618) was added to the in situ 3C reaction. To fluorescently stain nuclei, 1 µl of 1 mg ml^−1^ Hoechst 33342 was added before sorting. Fluorescence-activated nucleus sorting was carried out at the Broad Stem Cell Research Center Flow Cytometry core of the University of California, Los Angeles using BD FACSAria sorters. Single nuclei were sorted into 384-well plates containing 1 µl M-Digestion buffer containing proteinase K and about 0.05 pg lambda DNA isolated from dcm^+^ *Escherichia* *coli* (Promega number D1501).

### Single-nucleus DNA methylome library preparation with snmC-seq3

snmC-seq3 is a modification of snmC-seq2^43^ that provides improved throughput and reduced cost. Key differences between snmC-seq3 and snmC-seq2 include the usage of 384 instead of 8 barcoded degenerated (RP-H) primers (Supplementary Table 8) for the initiation of random-primed DNA synthesis using bisulfite-converted DNA as a template. The expanded multiplexing allows the combination of 64 single nuclei into the downstream enzymatic reactions, which provides an eightfold reduction of the usage of Adaptase and PCR reagents. In addition, the amounts of Klenow exo^−^, exonuclease 1 and rSAP are reduced by tenfold compared to snmC-seq2, further reducing reagent cost. A detailed bench protocol for snm3C-seq3 is provided through protocol.io (10.17504/protocols.io.kqdg3x6ezg25/v1).

### Probe library design for RNA MERFISH and chromatin tracing

We selected 40-bp target sequences for DNA or RNA hybridization by considering each contiguous 40-bp subsequence of each target of interest (the mRNA of a targeted gene or the genomic locus of interest) and then filtering out off-targets to the rest of the transcriptome or genome including repetitive regions, or too high or low GC content or melting temperature. More specifically, our probe design algorithm was implemented with three steps: build a 17-base index based on reference genome hs1 assembly (DNA) or the hg38 transcriptome (RNA); quantify 17-base off-target counts for each candidate 40-bp target sequence; filter and rank target sequences on the basis of predefined selection criteria as previously described^30,44^.

### MERFISH gene selection

The MERFISH gene selection was carried out by first using a BICCN dataset from GW-18–19 brain and using NSForest v2^45^ with default parameters to identify marker genes for the cell-type clusters in this data^46^. This list of genes was supplemented with additional marker genes from the literature (that is, *DCX*, *GFAP* and so on) as well as genes with differential methylation in the snm3C-seq3 data in HPC of mid-gestational human brains. The target sequences for each gene were concatenated with one or two unique readout sequences to facilitate MERFISH or single-molecule fluorescence in situ hybridization imaging. The final list of encoding probes for RNA imaging used is shown in Supplementary Table 5.

### Design of chromatin probes

We designed probes for DNA hybridization similarly to those for RNA MERFISH as described in refs. ^30,47^. Briefly, we first partitioned chromosome 14 into 50-kb segments and selected for imaging a fifth of these segments uniformly spaced every 250 kb (amounting to 354 target genomic loci using genome reference hs1). After screening against off-target binding, GC content and melting temperature, about 150 unique 40-bp target sequences were selected for each 50-kb segment. We concatenated a unique readout sequence to the target probes of each segment to facilitate sequential hybridization and imaging of each locus. The final list of encoding probes for DNA imaging used is shown in Supplementary Table 4.

### Primary probe synthesis

The encoding probes were synthesized from template oligonucleotide pools, following the previously described method^48^. First, we amplified the oligonucleotide pools (Twist Biosciences) using a limited cycle quantitative PCR (approximately 15–20 cycles) with a concentration of 0.6 µM of each primer to create templates. These templates were converted into the corresponding RNAs using the in vitro T7 transcription reaction (New England Biolabs, E2040S) and the PCR product as the templates. The resulting RNAs were then converted to complementary single-stranded DNA using reverse transcription. During the reverse transcription step, a primer with a 5′ acrydite-modified end was used to facilitate the incorporation of the encoding probes into a protective acrylamide gel cast on the sample allowing for more than 100 rounds of hybridization without substantial probe loss. Subsequently, the single-stranded DNA oligonucleotides were purified using alkaline hydrolysis to remove RNA templates and cleaned with columns (Zymo, D4060). The resulting probes were stored at −20 °C.

### Sample preparation for multiplexed imaging experiments

The samples were prepared similarly to previously described for cell culture samples with notable modifications^30^. Briefly, fresh frozen brain tissues were sectioned into coronal sections of 18 μm thickness at −20 °C using a Leica CM3050S cryostat. Sections were collected on salinized and poly-l-lysine (Millipore, 2913997)-treated 40-mm, round number 1.5 coverslips (Bioptechs, 0420-0323-2). The tissue sections were fixed with 4% PFA (Electron Microscopy Sciences, 15710) in 1× PBS with RNase inhibitors (New England Biolabs, M0314L) for 10 min at room temperature before being permeabilized with 0.5% Triton X-100 (Sigma-Aldrich, T8787). Then coverslips were treated with 0.1 M hydrochloric acid (Thermo Scientific, 24308) for 5 min at room temperature. Tissue sections were next incubated with pre-hybridization buffer (40% (vol/vol) formamide (Ambion, AM9342) in 2× SSC (Corning, 46-020-CM)) for 10 min. Then 50 μl of encoding probe hybridization buffer (50% (vol/vol) formamide (Ambion, AM9342), 2× SSC, 10% dextran sulfate (Millipore, S4030)) containing 15 μg of RNA-encoding probes for the targeted genes and 150 μg of DNA-encoding probes for the targeted chromosome 14 loci was incubated with the sample first at 90 °C for 3 min, followed by 47 °C for 18 h. The sample was then washed with 40% (vol/vol) formamide in 2× SSC, 0.5% Tween 20 for 30 min before being embedded in thin, 4% polyacrylamide gels as described previously^49^.

### Imaging and adaptor hybridization protocol

MERFISH measurements were conducted on a custom microfluidics–microscope system with the configuration described previously described^30,44^. Briefly, the system was built around a Nikon Ti-U microscope body with a Nikon CFI Plan Apo Lambda 60× oil immersion objective with 1.4 NA and used a Lumencor CELESTA light engine and a scientific CMOS camera (Hamamatsu FLASH4.0). The different components were synchronized and controlled using a National Instruments data acquisition card (NI PCIe-6353) and custom software^30^.

To enable multimodal imaging, we first sequentially hybridized fluorescent readout probes and then imaged the targeted genomic loci and then the targeted mRNAs. Then we carried out a series of antibody stains and imaging. Specifically, the following protocol was used in order: 118 rounds of hybridization and chromatin tracing imaging, sequentially targeting the 354 chromosome 14 loci using three-colour imaging; 16 rounds of hybridization and MERFISH imaging, combinatorially targeting 298 genes using three-colour imaging; 3 rounds of sequential staining for 6 different antibodies using two-colour imaging.

The protocol for each hybridization included the following steps: incubate the sample with adaptor probes for 30 min for DNA imaging or 75 min for RNA imaging at room temperature; flow wash buffer and incubate for 7 min; incubate fluorescent readout probes (one for each colour) for 30 min at room temperature; flow wash buffer and incubate for 7 min; flow imaging buffer. The imaging buffer was prepared as described previously^30^ and additionally included 2.5 μg ml^−1^ 4′,6-diamidino-2-phenylindole (DAPI).

Following each hybridization, the sample was imaged, and then the signal was removed by flowing 100% formamide for 20 min and then re-equilibrating to 2× SSC for 10 min.

RNA MERFISH measurement of the 298-gene panel was carried out with an encoding scheme of 48-bit binary barcode and a Hamming weight of 4. Therefore, in each hybridization round, about 75 adaptor probes were pooled together to target a unique subset of the 298 genes. The genes targeted in each round of hybridization are highlighted in Supplementary Table 9.

### Immunofluorescence staining

Antibody imaging was carried out immediately after completing the DNA and RNA imaging. The sample was first stained for 4 h at room temperature using two primary antibodies of two different species (mouse and rabbit), washed in 2× SSC for 15 min and then stained for 2 h using two secondary antibodies for each target species conjugated with fluorescent dyes. Supplementary Table 10 lists all of the antibodies used in this study.

### MERFISH image acquisition

We imaged approximately 650 fields of view covering the HPC. After each round of hybridization, we acquired *z*-stack images of each field of view in four colours: 750 nm, 647 nm, 560 nm and 405 nm. Consecutive *z*-sections were separated by 300 nm and covered 15 μm of the sample. Images were acquired at a rate of 20 Hz.

### Processing of snm3C-seq3 data

Sequencing reads were first demultiplexed by matching the first 8 bp of R1 reads to the predefined well barcodes (https://github.com/luogenomics/demultiplexing). Demultiplexed reads were trimmed to remove sequencing adaptors using Cutadapt 1.18 with the following parameters in paired-end mode: -f fastq -q 20 -m 50 -a AGATCGGAAGAGCACACGTCTGAAC -A AGATCGGAAGAGCGTCGTGTAGGGA. Then 18 bp and 10 bp were further trimmed from the 5′- and 3′-end of the R1 reads, respectively; and 10 bp were trimmed from both the 5′- and 3′- ends of the R2 reads. snm3C-seq3 reads were mapped to the hg38 reference genome using a modified Taurus-MH package (https://github.com/luogenomics/Taurus-MH)^3^. Briefly, each read end (R1 or R2) was mapped separately using Bismark with Bowtie1 with read1 as complementary (always G to A converted) and read2 (always C to T converted) as the original strand. After the first alignment, unmapped reads were retained and split into three pieces by 40 bp, 42 bp and 40 bp resulting in six subreads (read1 and read2). The subreads derived from unmapped reads were mapped separately using Bismark Bowtie1. All aligned reads were merged into BAM using the Picard SortSam tool with query names sorted. For each fragment, the outermost aligned reads were chosen for the chromatin conformation map generation. The chromatin contacts with both ends mapped to the same positions were considered duplicates and removed for further analysis. Duplicated reads were removed from BAM files using the Picard MarkDuplicates tool before the generation of allc files using the Allcools bam-to-allc tool (https://lhqing.github.io/ALLCools/)^26^.

### Single-molecule fluorescence in situ hybridization

Single-molecule fluorescence in situ hybridization was carried out according to the RNAscope manual (multiplex details). Sequences of target probes, preamplifiers, amplifiers and label probes are proprietary and commercially available (Advanced Cell Diagnostics (ACD)). Typically, the probes contain 20 ZZ probe pairs (approximately 50 bp per pair) covering 1,000 bp. Here we used probes against human genes as single-plex probes, outlined below: Hs-MEF2C (452881), Hs-GAD1-C2 (404031-C3), Hs-RBFOX3-C2 (415591-C2), Hs-TLL1-C3 (439211), Hs-TRPS1 (831611-C3), Hs-PROX1 (530241), Hs-ALDH1L1-C3 (438881-C3), Hs-LRIG1-C2 (407421-C2).

Slides were dried at 60 °C for 1 h and fixed in 4% PFA for 2 h. After several washes in PBS, slides were treated with ACD hydrogen peroxide for 10 min and then washed in water twice before treatment in 1× target retrieval buffer (ACD) for 5 min (at 95–100 °C). After being washed in water and then 100% alcohol, the slides were baked at 60 °C for 30 min. After moistening samples with water, protease treatment was carried out for 15 min at 40 °C in a HybEZ oven. Hybridization of probes and amplification was carried out according to the manufacturer’s instructions. In short, tissue sections were incubated in the desired probe (2–3 drops per section) for 2 h at 40 °C in the HybEZ oven. The slides were washed twice in 1× wash buffer (ACD) for 2 min each and incubated in 5× SSC at room temperature overnight. Amplification and detection steps were carried out using the Multiplex kit (ACD, 320293) for single-plex probes. The following was carried out in repeated cycles for each probe. About four drops of AMP x-FL were added to entirely cover each section and the slide was placed in the HybEZ oven. The slide was incubated for 30 min at 40 °C. Slides were removed from the HybEZ slide rack, and excess liquid was removed before being submerging them in a Tissue-Tek staining dish filled with 1× wash buffer. Slides were washed in 1× wash buffer for 2 min at room temperature. The next AMP x-FL treatment was added, and the cycle was repeated. Slides were washed in PBST, incubated with DAPI for 30 s at room temperature, and mounted in aqua mount (Lerner). Images were taken using a 100× objective on a Leica Stellaris confocal microscope.

### Single-cell bimodal data quality control and preprocessing

Cells were filtered on the basis of several metadata metrics: mCCC level < 0.03; global CG methylation level > 0.5; global non-CG methylation level < 0.2; and total 3C interactions > 100,000. Methylation features were calculated as fractions of methylcytosine over total cytosine across gene bodies ±2 kb flanking regions and 100-kb bins spanning the entire genome. Methylation features were further split into CG and CH methylation types. These features were then filtered on mean coverage of ≥10 and values with coverage of <5 were imputed as the mean feature value by sample. Principal component analysis (PCA) was then run using Scanpy^50^ default parameters followed by *k*-nearest neighbours using only the top 20 principal components by the amount of variance explained and *k* = 15. Iterative clustering was then carried out with a combination of Leiden unsupervised clustering and UMAP dimensionality reduction, identifying clusters as cell types by marker gene body CH and CG hypomethylation. We observed certain batch effects in our dataset that are associated with the time the data were generated. Harmony^22^ was used on metadata features to mitigate batch effects occurring between samples in the principal-component feature space. The developmental trajectories of cortical and hippocampal cell types were reconstructed using shared CG methylation feature patterns at cell-type marker genes and integration of cells derived from different age groups using Harmony^22^. Cells were first separated by their L2 (major cell-type groups) annotation using the shared marker gene approach, and then Harmony integration by pairwise ages for all L2 groups was used to link L3 cell types across ages.

### Integration of snm3C-seq3 data with datasets for single-nucleus RNA-sequencing or single-nucleus assay for transposase-accessible chromatin with sequencing

The single-nucleus RNA (snRNA) and prenatal snm3C-seq CG methylation data were co-embedded by inverting the sign of the methylation matrix (owing to an inverse correlation of gene expression to CG methylation). PCA was then applied on the combined data, and Harmony^22^ was used to correct for the systematic differences between the two modalities. The co-embedded UMAP was then generated from the *k*-nearest neighbours graph with *k* = 20 using the top 20 principal components. Annotation transfer was carried out for each cell in the CG methylation data by taking the cell’s top snRNA neighbour and assigning the RNA label to the CG methylation cell. The Jaccard index was then computed on the CG methylation annotation versus the snRNA liftover annotation. The correlation of features between the two modalities was calculated by taking the *k* = 1 nearest neighbour for all methylation cells and computing the Pearson correlation of raw CG methylation fraction to log-scaled gene expression counts for all genes across all paired cells. Generalized annotations were then made for the co-embedding by running Leiden unsupervised clustering and naming the clusters by their most representative cell type to assess the relative quantity of similar cells in each dataset. The co-embedding of the single-nucleus assay for transposase-accessible chromatin (ATAC) and snm3C-seq CG methylation data followed the same procedure as the snRNA co-embedding, but the Pearson correlation was computed on the raw ATAC gene activity scores versus raw CG methylation fractions.

### Pseudotime analysis

Pseudotime analysis was run following the methods outlined in ref. ^24^. Each pseudotime analysis had clustering preprocessing steps, PCA, *k*-nearest neighbours with *k* = 15 using 20 principal components, and Leiden, recomputed for its respective subset of the data. The computed Leiden clusters were then used to initialize a partition-based graph abstraction. This partition-based graph abstraction is used as the precomputed initialization coordinates for the visualization with force-directed graph drawing by the ForceAtlas2 package^51^. A root node is then set in the Leiden cluster furthest from the adult cell types, and Scanpy’s implementation of diffusion-based pseudotime was used. In multimodal pseudotimes the same cell is set as the root node in each modality. Genes are selected for display compared to the pseudotime scores by sorting by correlation and anticorrelation to the pseudotime score as well as requiring the 3CGS to have variance of >0.1 and gene length of >90 kb. For Fig. 2n and Supplementary Fig. 4j,u, gene examples were selected by highest gene body CG methylation correlation to the pseudotime and 3CGS anticorrelation. For Fig. 2o and Supplementary Fig. 4k,v, gene examples were selected by highest gene body CG methylation anticorrelation to the pseudotime and 3CGS correlation to the pseudotime. Distribution comparisons are computed by the Wilcoxon rank-sum test.

### DMR and TF-binding motif analysis

All CG methylation DMRs were identified from pseudobulk allc files using Methylpy (https://github.com/yupenghe/methylpy)^52^. DMRs identified from a multi-sample comparison of all cell types were used for analyses in Fig. 5, as well as disease heritability enrichment analyses. Trajectory-DMRs were identified using pairwise comparisons of adjacent development stages of a cell-type trajectory. Branch-DMRs were identified using multi-sample comparisons, including the mother cell population from an earlier developmental stage and daughter populations from a later developmental stage. TF-binding motif enrichment analysis was carried out similarly to previously described^17,25,53^. DMR regions were lifted over to the hg19 reference genome for the TF-binding motif enrichment analysis. TF-binding motif position weight matrices were obtained from the MEME motif database and scanned across the human hg19 reference genome to identify hits using FIMO (--output-pthresh 1E-5, -- max-stored-scores 500,000 and --max-strand)^54,55^. DMRs were extended 250 bp both upstream and downstream for overlapping with TF-binding motif hits. The overlap between TF-binding motif hits and DMRs (extended ±250 bp) was determined by requiring a minimum of 1-bp overlap. The enrichment of TF-binding motifs in DMRs was assessed using DMRs (extended 250 bp from centre) identified across adult human tissues (tissue DMRs) as the background^52^. The overlaps between TF-binding motif hits and the foreground DMR list were compared to the overlaps between TF-binding motif hits and tissue DMRs (background) using the hypergeometric test (MATLAB hygecdf).

### Single-cell embedding based on chromatin contact

Single-cell contact matrices at 100 kb resolution were imputed by scHiCluster^56^ with pad = 1. The imputed contacts with distance of >100 kb and <1 Mb were used as features for singular value decomposition dimension reduction. Principal components were normalized by singular values and L2 norms per cell and then used for *k*-nearest neighbour graph construction (*k* = 25) and UMAP. A total of 25 dimensions were used for the full dataset (Fig. 1 and Extended Data Fig. 2b,e), 20 dimensions were used for the RG subtypes (Extended Data Fig. 2f) and 10 dimensions were used for the MGE or CGE lineage (Extended Data Fig. 2d).

### Chromatin loop, differential loop and SIP analysis

Chromatin loops were identified with scHiCluster^56^ for each cell type identified in this study. To identify loops from a group of cells, single-cell contact matrices at 10 kb resolution were imputed by scHiCluster with pad = 2 for the contacts with a distance less than 5.05 Mb (result denoted as *Q*_cell_). We carried out loop calling only between 50 kb and 5 Mb, given that increasing the distance leads to only a limited increase in the number of statistically significant loops. For each single cell, the imputed matrix of each chromosome (denoted as *Q*_cell_) was log-transformed, *Z*-score-normalized at each diagonal (result denoted as *E*_cell_) and a local background between >30 kb and <50 kb was subtracted (result denoted as *T*_cell_), as in SnapHiC^57^. We then generated pseudobulk matrices for each sample by taking the average across single cells. To compute the variance of each matrix across single cells in loop and differential loop analysis, for each pseudobulk sample, we saved both the mean and mean of squares. Specifically, six pseudobulk matrices were generated as ${Q}_{{\rm{bulk}}}=\sum {Q}_{{\rm{cell}}}/{n}_{{\rm{cell}}}$, ${Q2}_{{\rm{bulk}}}=\sum {Q}_{{\rm{cell}}}^{2}/{n}_{{\rm{cell}}}$, ${E}_{{\rm{b}}{\rm{u}}{\rm{l}}{\rm{k}}}=\sum {E}_{{\rm{c}}{\rm{e}}{\rm{l}}{\rm{l}}}/{n}_{{\rm{c}}{\rm{e}}{\rm{l}}{\rm{l}}}$, ${E2}_{{\rm{b}}{\rm{u}}{\rm{l}}{\rm{k}}}=\sum {E}_{{\rm{c}}{\rm{e}}{\rm{l}}{\rm{l}}}^{2}/{n}_{{\rm{c}}{\rm{e}}{\rm{l}}{\rm{l}}}$, ${T}_{{\rm{bulk}}}=\sum {T}_{{\rm{cell}}}/{n}_{{\rm{cell}}}$, ${T2}_{{\rm{bulk}}}=\sum {T}_{{\rm{cell}}}^{2}/{n}_{{\rm{cell}}}$. A pseudobulk-level *t*-statistic was computed to quantify the deviation of *E* and *T* from 0 across single cells from the cell group, with larger deviations representing higher enrichment against the global (*E*) or local (*T*) background. *E*_cell_ is also shuffled across each diagonal to generate *E*_shufflecell_, then minus local background for *T*_shufflecell_, and further merged into pseudobulks *E*_shufflebulk_, *E*2_shufflebulk_, *T*_shufflebulk_ and *T*2_shufflebulk_, to estimate a background of the *t*-statistics. *E*2_shufflebulk_ is defined as ${E2}_{{\rm{shufflebulk}}}=\sum {E}_{{\rm{shufflecell}}}^{2}/{n}_{{\rm{shufflecell}}}$. *T*2_shufflebulk_ is defined as ${T2}_{{\rm{shufflebulk}}}=\sum {T}_{{\rm{shufflecell}}}^{2}/{n}_{{\rm{shufflecell}}}$. An empirical false discovery rate (FDR) can be derived by comparing the *t*-statistics of observed cells versus shuffled cells. We required the pixels to have average *E* of >0, fold change of >1.33 against doughnut and bottom left backgrounds, fold change of >1.2 against horizontal and vertical backgrounds^57^, and FDR of <0.01 compared to global and local backgrounds.

Differential loops were identified between age groups in the same major lineage. The detailed analysis framework is shown at https://zhoujt1994.github.io/scHiCluster/hba/loop_majortype/intro.html. To compare the interaction strength of loops between different groups of cells, analysis of variance or a Kruskal–Wallis test can be used. This test is more generalizable, as it does not require the data to be normally distributed. However, in practice, it is very expensive computationally to enumerate through all cells and all loops to run the tests. Therefore, we adopt an analysis of variance framework to compute the *F* statistics for each loop identified in at least one cell group using either *Q*_cell_ (result denoted as *F*_Q_) or *T*_cell_ (result denoted as *F*_T_). This analysis requires only *Q*_bulk_, *Q*2_bulk_, *T*_bulk_ and *T*2_bulk_ for each pseudobulk sample to capture the variability across cells rather than the matrices of each single cell, which makes it feasible across thousands of cells and millions of pixels. We log-transformed and then *Z*-scored *F*_Q_ and *F*_T_ across all of the loops being tested and selected the ones with both *F*_Q_ and *F*_T_ > 1.036 (85th percentile of standard normal distribution) as differential loops. The threshold was decided by visually inspecting the contact maps as well as the correlation of interaction and loop anchor CG methylation. These thresholds selected the top ≈5% loops as differential for downstream analyses.

To identify SIPs, annotated transcription start sites from GENCODE v33 annotation were intersected with chromatin loops, allowing a maximum distance of 5 kb. To determine a threshold of cumulative loop score for SIPs, the cumulative loop score of promoters was modelled by a half-Gaussian distribution with the mean equal to 0 and standard deviation equal to the standard deviation of cumulative loop scores. The threshold for SIP was selected with a *P* value of 0.001.

### Identification of domains and differential domain boundaries

Single-cell contact matrices at 25 kb resolution were imputed by scHiCluster^56^ with pad = 2 for the contacts with distance less than 10.05 Mb. Domains were identified for each single cell. Insulation scores were computed in each cell group (major type or major type in a brain region) for each bin with the pseudobulk imputed matrices (average over single cells) and a window size of 10 bins. The boundary probability of a bin is defined as the proportion of cells having the bin called as a domain boundary among the total number of cells from the group.

To identify differential domain boundaries between *n* cell groups, we derived an *n* × 2 contingency table for each 25-kb bin, in which the values in each row represent the number of cells from the group that has the bin called as a boundary or not as a boundary. We computed the chi-square statistic and *P* value of each bin and used the peaks of the statistics across the genome as differential boundaries. The peaks are defined as the local maximum of chi-square statistics with an FDR of <1 × 10^−3^ (Benjamini and Hochberg procedure). If two peaks are within less than five bins of each other, we kept only the peak with a higher chi-square statistic. We also require the peaks to have a *Z*-score-transformed chi-square statistic of >1.960 (97.5th percentile of standard normal distribution), fold-changes between maximum and minimum insulation score of >1.2, and differences between maximum and minimum boundary probability of >0.05.

### 3CGS

3CGS is defined as the sum of off-diagonal values from the row and column of the transcription start site bin to the row and column of the transcription end site bin in the imputed 10-kb contact matrices.

### Correlation between loop strength and CG methylation

Differential loops for cell-type trajectories were used in these analyses (Supplementary Table 6), with each trajectory analysed separately. Single cells were grouped into meta-cells ordered by the developmental pseudotime scores to boost the power of correlation analyses. Each meta-cell is composed of 20 single cells. Specifically, the joint embedding of CG methylation and chromosome conformation for each trajectory was used to generate a *k*-nearest neighbour (*k* = 20, self-included) graph of single cells. Each cell was merged with its other 19 nearest neighbours to generate a meta-cell. To avoid meta-cells that are highly similar to each other, we first computed the number of shared cells between each pair of meta-cells and removed highly similar meta-cells. We repeated this process until no pairs of meta-cells shared more than five cells. To further alleviate the bias towards large homogeneous cell populations, we downsampled the number of meta-cells that originated from RG-1 to half of the number.

The methylation level of a meta-cell at each 10-kb bin was computed by the sum of methylated basecalls divided by the sum of total basecalls over its 20 composed single cells. The average methylation levels of the two anchors were used to correlate with the loop interaction strength. The loop interaction strength of a meta-cell was the average of imputed interactions over the 20 composing single cells. The diffusion pseudotime of a meta-cell was also the mean across its composing single cells. The meta-cells were ordered according to the pseudotime, and the cross-correlation was used to measure the temporary discrepancy between chromatin conformation and DNA methylation changes during development. Intuitively, the cross-correlation measures the correlation between loop strength and CG methylation after shifting the DNA methylation values along the developmental axis for a certain distance. We used the argument of the minima of the cross-correlation for the loops having a negative correlation between interaction and CG methylation to evaluate their timing differences. If the loop interaction is changing before DNA methylation, the DNA methylation values need to be moved backward to maximize its (absolute) correlation with genome structure, therefore corresponding to a negative shift in our measurement. A left-skewed distribution indicates that interaction changes earlier than methylation and vice versa.

### Distribution of the distance between interacting loci analysis

To count the number of *cis* (intra-chromosomal) contacts in each cell and bulk Hi-C data^28^, we divided the contacts into 143 logarithmic bins, the first of which was for contacts that were separated by less than 1 kb. Each subsequent bin covered an exponent step of 0.125, using base 2. Contacts in bins 1–37 were determined to be noisy and were eliminated, leaving bins 38–141 as valid bins.

The following metrics were used for the following analysis: percentage median, the percentage of contacts in bins 38–89 out of all valid bins; percentage long, the percentage of contacts in bins 90–141 out of all valid bins.

Cells were clustered by the distribution of their distance between interacting loci (*k*-means, *k* = 10) and reordered by the average value of log_2_[percentage median/percentage long] of each cluster (Fig. 3a–d and Extended Data Fig. 4a–c).

Each cell was assigned to a group by the following criteria (Fig. 3e,f and Extended Data Fig. 4d–k): SE, log_2_[percentage median/percentage long) > 0.4; INT, −0.4 > log_2_[percentage median/percentage long) > 0.4; LE, log_2_[percentage median/percentage long) < −0.4.

To find the clusters enriched in each cell type, we first calculated the percentage belonging to each cluster by cell type (Extended Data Fig. 4d). The enrichment score was obtained by normalizing the fraction of each cell type by the relative cluster sizes. For each cell type, an average of log_2_[percentage near/percentage long] scores (representing the ratio of median to long-range interactions) for all individual cells of that type was computed to compare with compartmentalization metrics.

We have computed empirical *P* values to determine the significance of *k*-means clusters showing distinct distributions of chromatin contact distances (Fig. 3a and Extended Data Fig. 4c). For each pair of clusters, we randomly selected one cell from each cluster. This process was repeated 1,000 times for each cluster pair. Cell pairs whose chromatin contract distribution showed a Pearson’s correlation coefficient greater than 0.8 were considered similar. For each pair of clusters, we counted the number of times (out of 1,000) that the correlation coefficient exceeded the threshold. This gave an estimate of the similarity in cell patterns between the two clusters.

### Chromatin compartment detection and analysis

Pseudobulk .cool files for all cell types were generated and balanced with cooler v0.8.3 at 1 Mb resolution^58^. For each cell type, compartments were assigned with cooltools v0.5.1, through eigenvector decomposition of each chromosome’s *cis*-interaction matrix^59^. Each 1-mb genomic bin (excluding chromosomes X, Y and M) was assigned to the A or B compartment by the sign of its chromosome’s eigenvector that has the highest Pearson correlation with GC content. A positive sign indicates bin membership in the A compartment; a negative sign indicates bin membership in the B compartment. We use the magnitude of the eigenvector value as the strength of compartment assignment. Using cooltools, we generated saddle plots, which visualize the distribution of observed/expected (O/E) contact frequency between genomic bins stratified by their eigenvector value. For pseudobulk files with more than 30 million contacts, we subset each matrix’s bins whose assignment strengths are in the top 20th percentile for their compartment. Then, we find the sum of O/E values of AA, BB, AB and BA interactions between these bins. For computing AA or BB O/E interaction dominance, we find the fraction of O/E signal explained by these AA or BB interactions, respectively, out of the total O/E signal for the pseudobulk matrix (Extended Data Fig. 6b). Similarly, the formula for compartmentalization strength score is: (sum(AA O/E) + sum(BB O/E))/(sum(BA O/E) + sum(AB O/E)) (Extended Data Fig. 6a,e). When computed on pseudobulk files with more than 30 million contacts, compartmentalization strength scores had no significant correlation with total pseudobulk contacts (Pearson correlation = 0.18, *P* = 0.10). Two-sided Mann–Whitney *U*-tests were used to compare distributions of these metrics between groups of cell types (Extended Data Fig. 6b,e).

Differential compartments were identified across all age groups for each major (L2) lineage using dcHiC v2.1 at 100 kb resolution (adjusted *P* < 0.01)^60^. These results were used to identify 100-kb genome bins that transitioned from the A compartment to the B compartment (AB transition) or vice versa (BA transition) between the earliest and latest ages in each lineage (Extended Data Fig. 6h). For each lineage, the transitioning bins’ CG methylation levels were computed at each age and normalized by subtracting the CG methylation level at the earliest age. The distribution of CG methylation levels for AB versus BA transitions at each age and lineage was compared with two-sided Mann–Whitney *U*-tests (Extended Data Fig. 6j–l).

### Polygenic heritability enrichment analysis

Polygenic heritability enrichment of DMRs and/or chromatin loops was analysed using a stratified linkage disequilibrium score regression (S-LDSC)-based partitioned heritability approach^61^. The genome-wide association study (GWAS) summary statistics included in this study were as follows: schizophrenia^40^, bipolar disorder^62^, major depressive disorder^63^, attention deficit hyperactivity disorder^64^, autism spectrum disorder^65^, Alzheimer’s disease^66^ and height from the UK Biobank^67^ (downloaded from https://alkesgroup.broadinstitute.org/sumstats_formatted/). For each cell type, binary annotations were created using DMR and/or chromatin loop. We considered two types of genomic region—DMR: including all DMRs for a given cell type; loop-connected DMR: including the subset of DMRs that overlap with any of the chromatin loop-called in the matching cell types. To create binary annotations, SNPs in these genomics regions were assigned as 1 and otherwise 0. Then we assessed the heritability enrichment of each of these annotations conditional on the ‘baseline model’^39^. We reported heritability enrichment and proportion of heritability using Enrichment, Enrichment_std_error, Prop._h2, Prop._h2_std_error columns in S-LDSC results. To assess statistical significance for heritability enrichment differences across annotations (for example, differences between cell types in a developmental trajectory), we used a *t*-test to test the differences of heritability enrichment of two cell types with d.f. = 200 + 200 − 2, in which 200 corresponds to the number of jackknife samples in the S-LDSC block jackknife procedure.

### Overlap between fine-mapped variants and DMR and/or chromatin loop for schizophrenia

We used statistical fine-mapping results that were previously performed in the latest PGC schizophrenia study^40^. We filtered for autosomal high-confidence putative causal SNPs with posterior inclusion probability of >10%, and retained 190 independent association loci (containing 569 SNPs in total), with each loci containing a credible set with 3.0 SNPs on average. We used Fisher’s exact test to assess the overlap between these 569 fine-mapped SNPs and DMR and/or chromatin loop annotations using all SNPs in the GWAS summary statistics as the background (see above for constructing DMR and/or chromatin loop annotations). We reported odds ratios of the overlap. We also assessed the overlap between 190 schizophrenia fine-mapped loci (as aggregates of 569 putative causal SNPs) and DMR and/or chromatin loop annotations (Fig. 5i). We define the overlap between fine-mapped loci and DMR and/or chromatin loop annotations on the basis of whether any high-confidence putative causal SNP in the fine-mapped loci is located in the annotation. Furthermore, we overlapped putative causal SNP and DMR and/or chromatin loop annotations to Genotype-Tissue Expression high-confidence fine-mapped *cis*-expression quantitative trait locus (eQTL) data (downloaded from https://www.gtexportal.org/home/downloads/adult-gtex/qtl): we first identified SNP–gene pairs such that the putative causal SNP is located in DMRs and connected to the transcription start site of any gene through chromatin loops, and then we overlapped these SNP–gene pairs with *cis*-eQTL–eGene pairs.

### Chromatin tracing analysis

#### Localization of fluorescent spots

To calculate fluorescent spot localizations for chromatin tracing data, we carried out the following computational steps: we computed a point spread function for our microscope and a median image across all fields of view for each colour channel based on the first round of imaging to be used for homogenizing the illumination across the field of view (called flat-field correction); to identify fluorescent spots, the images were flat-field-corrected, deconvoluted with the custom point spread function, and then local maxima were computed on the resulting images. A flat-field correction was carried out for each colour channel separately.

#### Image registration and selection of chromatin traces

Imaging registration was carried out by aligning the DAPI channel of each image from the same field of view across imaging rounds. First, the local maxima and local minima of the flat-field-corrected and deconvolved DAPI signal were calculated. Next, a rigid translation was calculated using a fast Fourier transform to best align the local maxima or minima between imaging rounds.

Nuclear segmentation was carried out on the DAPI signal of the first round of imaging using the Cellpose algorithm^68^ with the ‘nuclei’ neural network model. Following image registration, chromatin traces were computed from the drift-corrected local maxima of each imaged locus as previously described^30^.

### RNA MERFISH analysis

The MERFISH decoding followed a similar strategy to that of the MERlin algorithm but operated on spots identified in the images rather than individual pixels. Briefly, the drift- and chromatic-aberration-corrected local maxima (spots) were grouped into clusters, with each cluster containing all spots from all imaging rounds in a 2-pixel radius of an anchor spot. Clusters were generated for every possible anchor spot. Any cluster containing spots from at least four images was then assigned a gene identity by best matching the MERFISH codebook. Each cluster was ranked by the average brightness and the interdistance between the contained spots. These measures were used to filter the decoded cluster and best separate the more confident spots from the less confident.

### Protein density quantification

The antibody images were flat-field-corrected, deconvolved and then registered to the chromatin traces using the DAPI signal as described previously. For each chromatin trace, the fluorescent signal of each antibody was sampled at the 3D location corresponding to each genomic locus.

### Refinement of the nuclear segmentation

Refined 3D nuclear segmentation was carried out using the 3D Cellpose ‘nuclei’ model based on the NUP98 fluorescent stain.

### Integration of snmC-seq3 and RNA MERFISH

RNA profiles generated by MERFISH were unbiasedly clustered using the Leiden method and annotated on the basis of known marker genes shown in Supplementary Fig. 5a. The MERFISH transcriptomic data were then integrated with the data from mid-gestation HPC snmC-seq3 methylation samples by subsampling a set of 220 shared genes, inverting the methylation matrix and using Harmony^22^ to correct the systematic differences between the two modalities. RG-1 and RG-2 annotations were generated by label transfer from the methylation data using *k* = 9 nearest-neighbour majority voting in the batch-corrected UMAP space. Label transfer was blocked in mature cell types (dentate gyrus, CA1 and CA2–3) and those not derived from hippocampal RG cells (ependyma and choroid plexus).

### MERFISH data availability

Raw imaging data will be provided on request owing to the extraordinary file sizes. Processed data are available at the Gene Expression Omnibus under the accession number GSE213950 as a scanpy.h5ad file. The main ‘.X’ matrix of the object contains log-normalized counts. The full contents of the scanpy object are described below. For brevity, standard contents added by scanpy (for example, connectivities and distances added by sc.pp.neighbors) are not listed.

- obs
  - volm: total pixel volume of the cell based on DAPI segmentation;
  - x_um_abs, y_um_abs: global *x* and *y* coordinates of the cell in micrometres;
  - zc, xc, yc: pixel coordinates of the cell centre relative to the field of view;
  - Leiden: unsupervised Leiden clustering;
  - L1: excitatory versus inhibitory;
  - dpt_pseudotime: pseudotime calculated from RG-1;
  - Final_anno_v3: annotation used in figures;
  - Hpc_regional: spatial subset of cells restricted to the HPC;
  - hpcRG: RG-1 and RG-2 annotation in this zone;
  - Fimbria_regional: spatial subset of cells restricted to the HPC;
  - fimbriaRG: RG-1 and RG-2 annotation in this zone;
  - Ventricular_regional: spatial subset of cells restricted to the ventricular zone;
  - ventricularRG: RG-1 and RG-2 annotation in this zone;
  - Refined_volume: Recalculated cell volume based on Nup98 antibodies.

- var
  - mean: average expression of the gene across cells;
  - std: standard deviation of the gene expression across cells.

- uns
  - X_h_score_shape: original shape of X_h_score in obsm;
  - antibody_shape: original shape of each antibody matrix in obsm.

- obsm
  - X_fov: the field-of-view identifier each cell was imaged in;
  - X_raw: raw count matrix;
  - X_spatial: the spatial coordinates of the cells;
  - blank: the count of each blank barcode per cell;
  - X_h_score: a csr sparse matrix containing chromatin trace results. The matrix should be reshaped to 50,374 ×4 × 354 × 5, representing the number of cells, maximum number of homologues, number of chromatin regions, and the *z*, *x*, *y* coordinates followed by the brightness and score of the fluorescent spot. Missing data (that is, containing fewer than four homologues or missing regions) are filled with 0 s;
  - H3 K9 trimethylation, Pol2PSer2, SRSF2, K27Ac, LAMA1, NUP98: antibody signals localized at each chromatin region. Stored as a csr sparse matrix and can be reshaped to 50,374 × 4 × 354, similar to X_h_score.

### Reporting summary

Further information on research design is available in the Nature Portfolio Reporting Summary linked to this article.

## Online content

Any methods, additional references, Nature Portfolio reporting summaries, source data, extended data, supplementary information, acknowledgements, peer review information; details of author contributions and competing interests; and statements of data and code availability are available at 10.1038/s41586-024-08030-7.

## Supplementary information

Supplementary Information Supplementary Notes 1–12 and Figs 1–12.

Reporting Summary

Supplementary Table 1 Human brain specimen used in this study.

Supplementary Table 2 snm3C-seq3 metadata.

Supplementary Table 3 Cell-type annotation.

Supplementary Table 4 Probes for chromatin tracing.

Supplementary Table 5 Probes for RNA MERFISH.

Supplementary Table 6 DNA methylation and chromatin conformation features identified in the study.

Supplementary Table 7 Results for overlapping schizophrenia fine-mapped high-confidence putative causal loci (posterior inclusion probability of >10%) with DMR, chromatin loop or eQTL.

Supplementary Table 8 Sequences of 384-plex RP-H primers.

Supplementary Table 9 RNA MERFISH codebook.

Supplementary Table 10 Antibodies used in multimodal DNA, RNA and protein imaging.

## Data Availability

Datasets generated by this study can be accessed interactively through https://brain-epigenome.cells.ucsc.edu/ and https://genome.ucsc.edu/s/luogenomics/hs-brain-epigenome. Processed chromatin conformation data for all samples, processed single-cell DNA methylation data for samples with unrestricted access and processed multimodal MERFISH data are available at the National Center for Biotechnology Information Gene Expression Omnibus under the accession number GSE213950. Raw sequencing reads for all samples and processed DNA methylation data for controlled access samples can be downloaded from the NeMO Archive (https://assets.nemoarchive.org/dat-obec38w). Access to raw data for prenatal specimens analysed in this study is controlled as specified in the consent for tissue donation. Requests for controlled data hosted by the NeMO Archive can be made through the NIMH Data Archive (https://nda.nih.gov/). Access to controlled data associated with this study is permitted for general research use. Instructions for requesting access to controlled data hosted by NeMO are provided at https://nemoarchive.org/resources/accessing-controlled-access-data. Single-cell RNA-sequencing data for prenatal human cortical specimens were published in ref. ^21^. Data from the single-nucleus ATAC approach for prenatal human cortical specimens were published in ref. ^14^. Bulk Hi-C data for multiple human tissues were published in ref. ^28^. Bulk Hi-C data for neuronal and non-neuronal nuclei isolated from adult human brains were published in ref. ^29^. The BRAINSPAN developmental transcriptome dataset was published in ref. ^11^. The GWAS summary statistics included in this study were as follows: schizophrenia^40^, bipolar disorder^62^, major depressive disorder^63^, attention deficit hyperactivity disorder^64^, autism spectrum disorder^65^, Alzheimer’s disease^66^ and height from the UK Biobank^67^ (downloaded from https://alkesgroup.broadinstitute.org/sumstats_formatted/).

## Code availability

Codes for the demultiplexing of snm3C-seq3 fastq files are available at https://github.com/luogenomics/demultiplexing. A modified version of TAURUS-MH (version 0.1) for mapping of snm3C-seq3 data is available at https://github.com/luogenomics/Taurus-MH. Codes for the generation and imputation of methylation features are available at https://github.com/luogenomics/snm3Cseq_feature_processing. Custom code used for analysing chromatin tracing and MERFISH datasets in this study are available at https://github.com/cfg00/MERFISH_Chromatin_Tracing_2024.
