# Supplementary material for: Temporally distinct 3D multi-omic dynamics in the developing human brain
Source: Nature. 2024 Oct 9;635(8038):481–9. doi: 10.1038/s41586-024-08030-7 (PMC11560841; doi:10.1038/s41586-024-08030-7)
Supplement: Supplementary file 1 — Supplementary Notes 1–12 and Figs 1–12. [file 41586_2024_8030_MOESM1_ESM.pdf]

nature portfolio
https://doi.org/10.1038/s41586-024-08030-7

Supplementary information

Temporally distinct 3D multi-omic dynamics in the developing human brain

In the format provided by the
authors and unedited

Supplementary Note 1: Quantity assessment of snm3C-seq3 profiles.

Compared to snmC-seq2 [1], snmC-seq3 increased the throughput and reduced the cost of library preparation by expanding the multiplexing of single nuclei (from 8-plex to 384-plex) for 3'- adaptor tagging. snm3C-seq3 libraries generated with snmC-seq3 showed comparable mapping rate, enrichment of CpG islands, and genome-wide coverage uniformity as snm3C-seq libraries generated using snmC-seq2 (Supplementary Fig. 1a-c). On average, 21.0±5.3% and 13.3±3.0% of unique sequencing reads for each snm3C-seq3 profile represent intrachromosomal and interchromosomal chromatin interactions, respectively (Supplementary Fig. 1d). The rest, 65.7±5.6% reads are methylome reads containing no ligation junction. Each snm3C-seq3 methylome profile contains an average of 1.9 million reads, covering 4.1% of the human genome. Each snm3C-seq3 3C (Chromatin Conformation Capture) profile contains an average of 230,688 unique chromatin contacts, and the average ratio between intra- and inter-chromosomal interaction equals 1.58. Using cell-type classification information, we further assessed the metrics of 3C profiles in the context of cell type and developmental stages. The quality of snm3C-seq3 3C profiles was similar across brain specimens, as shown by the consistent distance distribution of intrachromosomal interactions across donors for each cell type (Supplementary Fig. 1e-h). Similarly, snm3C-seq3 methylation profiles generated from donors with similar ages and across experimental batches are strongly correlated (Supplementary Fig. 1i-j). Unexpectedly, we found that the ratio between intra- and interchromosomal interactions (intra/inter ratio) is correlated with cell types and donor age (Supplementary Fig. 1k-n), with excitatory neurons showing greater intra/inter ratio than inhibitory neurons and non-neuronal cells (Supplementary Fig. 1k), while adult samples showing significantly greater intra/inter ratio than mid-gestational samples (Supplementary Fig. 1l). The greater intra/inter ratios in adult brain samples are contributed by both increased intrachromosomal interactions and decreased interchromosomal interactions (Supplementary Fig. 1m-n).

Supplementary Note 2: DNA methylome remodeling in the hippocampus precedes that in the frontal cortex.

Using two independent GW39 samples, we have validated the observation that the accumulation of mCH in the developing hippocampus precedes that in the frontal cortex (Supplementary Fig. 2a-c). We further compared the dynamics of mCG in the two brain regions with genome-wide and gene-specific analyses. The clustering of gene body mCG readily separates PFC neuronal populations into prenatal and postnatal groups (Supplementary Fig. 2d), suggesting major reconfiguration of intragenic mCG between late-gestation and infant stages in PFC. A similar clustering analysis of HPC neuronal populations found that the remodeling of gene body mCG occurs in HPC between mid-gestation and late-gestation (Supplementary Fig. 2e). The majority of neuronal populations in the late-gestational HPC, including CA1, CA3, dentate gyrus (DG), Mossy cell, and medial ganglionic eminence derived inhibitory neurons (Inh-MGE), were grouped with infant and adult cells rather than with mid-gestational cell types (Supplementary Fig. 2e). To ask whether methylation remodeling in HPC also precedes that in PFC at individual loci, we selected genes whose mCG changes showed the strongest correlations with genome-wide mCG dynamics. All selected genes that show a gain of mCG during development underwent mCG remodeling in HPC ahead of PFC, as well as the vast majority (20/25) of genes showing developmental loss of mCG (Supplementary Fig. 2f,g). Together, we found the remodeling of CH and CG methylomes predominantly occurs between late gestation and infant stages, starting in the HPC and proceeding shortly thereafter in the PFC.

Supplementary Note 3: Inverse correlation between 3CGS (3C gene score) and gene body mCG.

Similar to the inverse correlation between gene body mCG and gene expression across individual cells [2], the distribution of 3CGS-mCG Pearson's correlation coefficient has a long tail of negative values with 40% of genes (3,593/8,908) that can be reliably quantified for both data types showing a significant negative correlation between 3CGS and gene body mCG ($p<0.01$, permutation of cell id, Fig. 2d). We found that genes with an inverse mCG-RNA correlation are also likely to show a negative 3CGS-mCG correlation (Pearson's $r = 0.47$, p-value=2.2×10^{-308}, Supplementary Fig. 3a), which likely explains the observation that cell-type and developmental-specific expression of marker genes commonly showed increased 3CGS and decreased mCG (Fig. 2e,f).

Supplementary Note 4: Canonical and new cell type markers were identified using the snm3C-seq3 dataset.

The analysis found canonical cell type markers for the adult brain, such as SATB2 for upper layer excitatory neurons (Exc-UL), TLE4 for deeper layer excitatory neurons (Exc-DL), SLC1A2 for astrocytes, MBP for ODC, PDGFRA for OPC (Fig. 2e,f). In addition, astrocytic markers ARP13A4, LRIG1, and TNC are more demethylated in RG-2 than in RG-1, which is consistent with the gliogenic characteristic of RG-2 (Fig. 2e,f).

Supplementary Note 5: **Temporal orders of epigenomic remodelings across diverse developmental trajectories.**

The excitatory neuronal lineages exhibit continuous gain of both mC and 3C pseudotime values across development (Supplementary Fig. 3b-f), whereas inhibitory neurons show a strong separation of mC and 3C dynamics, particularly during the late-gestation (Supplementary Fig. 3g,h). Unlike the protracted methylation remodeling observed in astrocyte differentiation (Fig. 2l,m), the mCG dynamics during the maturation of MGE-derived inhibitory neurons (Inh-MGE) exhibited a rapid transition between the methylation states of developing and mature cells (Supplementary Fig. 4a-b), with the vast majority of mCG pseudotime range traversed by a single cell type, Inh-MGE and predominantly during late gestation (Supplementary Fig. 4b). In contrast, the pseudotime for the 3C gene score was more uniformly spaced across the maturation of Inh-MGE cells (Supplementary Fig. 4c). Chromatin conformation pseudotime traverses the least amount of pseudotime distance in Inh-MGE with the majority of distance traveled before, in Inh-eMGE during mid-gestation (stage 1 in Supplementary Fig. 4d,e), or after, in Inh-MGE-ERBB4 during infant to adult stages (stage 2 in Supplementary Fig. 4d,e). Further, in agreement with our earlier analyses, we saw in the pseudotime scores that MGE-derived inhibitory neurons located in HPC matured more quickly than those located in PFC during late gestation, with the difference being more pronounced for mCG than for chromatin conformation (Supplementary Fig. 4b,c,f,g). The more rapid maturation of DNA methylation in hippocampal Inh-MGE cells during late gestation was validated in two GW39 cases (Supplementary Fig. 4h,i). Consistent with the two stages of chromatin conformation dynamics (Supplementary Fig. 4d,e), we found loci primarily lost 3C gene score during mid-gestation (Stage 1, Supplementary Fig. 4j) and predominantly gained 3C gene score during infant and adult stages (Stage 2, Supplementary Fig. 4k). A parallel analysis on a caudal ganglionic eminence (CGE) derived inhibitory neuron trajectory found similar results (Supplementary Fig. 4l-v). Lastly, distinct temporal patterns were also found during the differentiation of OPC and ODC. The differentiation of OPC predominantly involves mCG dynamics, whereas the transition from OPC to ODC is primarily composed of chromatin conformation changes (Supplementary Fig. 3i). In summary, we found diverse temporal orders of DNA methylation and chromatin conformation dynamics across cell type trajectories.

Supplementary Note 6: Identification of brain cell types using RNA-MERFISH profiles.

Unbiased clustering of the single-cell transcriptional profiles measured by RNA-MERFISH identified 10 cell types within the HPC (Fig. 4f). Using established marker genes, we annotated the cell types including radial glial cells, differentiated excitatory and inhibitory neurons, choroid

plexus cell population, ependymal cells, and multiple clusters of intermediate neuronal cell types undergoing specification (Fig. 4f). We further integrated the RNA-MERFISH profile with snm3C-seq3 DNA methylome using the K-Nearest Neighbors approach to identify cells in tissue that are most similar to the neural progenitor RG-1 and glial progenitor RG-2 populations. The accuracy of cell-type classification was validated by marker gene expressions and their highly distinct spatial localization patterns (Fig. 4g,h and Supplementary Fig.5a,b). In particular, RG-1 and RG-2 populations are enriched in the ventricular zone, the interface between DG and hilus, as well as in the fimbria (Fig. 4g and Supplementary Fig.5b). To best match the snm3C-seq3 analysis, we focused on HPC (excluding fimbria and choroid plexus structures) for the comparison of RG-1 and RG-2 (Supplementary Fig.5c-h). While neural progenitor markers TNC, VIM, and PTPRZ1 are expressed in both RG-1 and RG-2 (Supplementary Fig.5a), neuronal markers SYT1, GAP43, and NGRN are more abundant in RG-1 cells, whereas astrocyte markers AQP4, LRIG1, and SOX9 are more actively expressed in RG-2 cells (Supplementary Fig.5e-h), which is consistent with the neurogenic and gliogenic characteristics of RG-1 and RG-2, respectively.

Supplementary Note 7: Multi-modal protein imaging of mid-gestational of human brain tissue.

We imaged a set of nuclear architectural proteins or post-translational modifications and quantified their fluorescent density across the length of chromosome 14 in each cell type (Fig. 4q and Extended Data Fig. 5h). A correlation analysis of the protein markers separates repressive markers, including H3K9me3 and nuclear lamin components (LamA, Nup98), from active markers, including the epigenetic mark H3K27ac, the splicing factor (Sc-35), and the elongating RNA polymerase II (Pol2PSer2) (Fig. 4r, and Extended Data Fig. 5i-m). Quantifying nuclear volume using Nup98 antibody staining revealed that indeed, the differentiation of RG-1 to neurons was associated with a significant increase in nuclear volume ($p=5.4 \times 10^{-278}$, two-sided rank-sum test, Fig. 4s), which was hypothesized as a drive of the establishment of SE conformation in neurons. However, neuronal differentiation is also associated with a significant increase in H3K9me3, Nup98, LamA, Pol2PSer2, and Sc35 density on chromosome 14 (all p-value $< 1 \times 10^{-100}$, two-sided rank-sum test, Fig. 4t and Extended Data Fig. 5i-l). An unbiased correlation analysis between chr 14 conformation and total density of protein markers colocalized with the chromosome found the spatial distances of genomic loci separated by moderate genomic distances were best correlated with H3K9me3, whereas the spatial distances of distal regions were most strongly correlated with nuclear volume (Fig. 4u). In summary, multiple processes, including epigenomic remodeling, transcription, and change of nuclear volume, could modulate the level of chromatin compaction and interaction at different length scales within distinct cellular populations, resulting in unique developmental states.

Supplementary Note 8: Analysis of differential chromatin compartments.

In each cell type trajectory, on average, 1,726 regions were found to be associated with differential compartments, with a similar number of regions switching from A to B or from B to A compartment during development (Extended Data Fig. 6h). The compartmental dynamics are consistent with the process of neurodevelopment, as regions switching from A to B compartment during the differentiation of upper layer excitatory neurons were enriched in neuronal migration functions and overlapped with genes such as SOX2 and GLI3 that are crucial for prenatal neural differentiation (Extended Data Fig. 6i). Across the genome, regions switching from A to B compartment accumulated a greater amount of mCG in astrocytes and all excitatory neuron types except for Exc-DG, whereas the difference is more subtle for inhibitory neurons (Extended Data Fig. 6j-l). This result is consistent with the notion that DNA methylation reinforces inactive chromatin compartments [3,4].

Supplementary Note 9: Correlation between chromatin loop and DNA methylation dynamics.

To correlate loop dynamics with the DNA methylation, we found that for 26% to 67% of differential loops across cell type trajectories, the mCG level of loop anchor regions shows a significantly inverse correlation (empirical FDR < 0.01, permutation test) with the loop strength, whereas a positive correlation was observed for much fewer (4% to 16%) loops (Extended Data Fig. 8a). Consistent with genome-wide pseudotime patterns, the protracted mCG dynamics in astrocyte differentiation were supported by a chromatin loop analysis that found the majority of chromatin loops were reconfigured in mid-gestation when RG-1 differentiates to RG-2 (Extended Data Fig. 8b,c), the gain or loss of gene body mCG did not occur until late-gestation or later (Extended Data Fig. 8d). The lag in mCG remodeling was confirmed by a cross-correlation analysis that determined the amount of pseudotime shift required to maximize the inverse correlation between loop strength and mCG of the anchor regions (Extended Data Fig. 8e). The temporal order of chromatin loop and mCG dynamics also shows variation across cell types as the lag of mCG remodeling is only observed by developmentally lost loops during the differentiation of excitatory neurons (Extended Data Fig. 8f-i).

Supplementary Note 10: **Analysis of differential chromatin domain boundaries.**

We further identified chromatin domain boundaries in single cells at a 25kb resolution using scHiCluster and TopDom [5,6], and quantified the strength of a domain boundary (boundary probability) in a cell population as the proportion of cells having the position called a boundary (Supplementary Table 6). We further identified differential domain boundaries across major cell-type trajectories using a Chi-Square test-based approach (Extended Data Fig. 9a and Supplementary Table 6). Similar to what was observed in the differential chromatin loop analysis, the strengthening of SE conformation during neuronal differentiation led to much more gained domain boundaries than losses of boundaries in neuronal trajectories (Extended Data Fig. 9b). An inversed pattern was found in astrocyte differentiation, where the formation of LE configuration was associated with more loss of boundaries than gains of boundaries (Extended Data Fig. 9b). We recapitulated previous findings that chromatin domain boundaries often separate regions with reduced mCH levels from neighboring regions with mCH levels similar to the genome-wide average (Extended Data Fig. 9c) [3]. Extended Data Fig. 9d shows an example of the developmental strengthening of domains overlapping with astrocyte marker gene SLC1A2, which is associated with considerable loss of gene body mCG in the infant stage (Extended Data Fig. 9d). In contrast, the early developmental gene SOX11 is located at the boundary of two domains in RG-1. The boundary is diminished during development and becomes undetectable in late gestation (Extended Data Fig. 9e).

Supplementary Note 11: TF binding motif analysis of DMRs.

Excitatory and inhibitory cells from the prenatal brain and glial cells from all stages share a large number of DMRs that are enriched in binding motifs for EMX, LHX2, SOX, and DLX TFs (cluster 1 in Fig. 5a and Supplementary Fig. 6a,b). This is consistent with earlier analyses in Supplementary Fig. 2d,e showing that the epigenomic difference between excitatory and inhibitory neurons is moderate in the pre-natal brain and becomes much more pronounced in infant and adult brains (Supplementary Fig. 2d,e). While all excitatory populations share a group of pan-excitatory DMRs that are enriched in the Neurogenin and RFX binding motifs (cluster 3 in Fig. 5a and Supplementary Fig. 6a,b), most excitatory subtypes such as HPC-CA, PFC-DL (Deep-Layer), PFC-UL (Upper-Layer), and Mossy cells are associated with their sub-population specific DMRs (clusters 4-7 in Fig. 5a and Supplementary Fig. 6a,b). Consistent with a higher expression of LHX2 in the developing hippocampus [7], Exc-CA and Exc-DG show a stronger enrichment of LHX2 motifs than in cortical excitatory cells (Fig. 5b and Supplementary Fig. 6a,b).

Supplementary Note 12: Temporal patterns of mCG remodeling in trajectories of brain cell differentiation.

A distinct wave of repression of regulatory elements (gain of mCG DMRs) during the late-gestation was found during the differentiation of cortical inhibitory neurons (Fig. 5b-d and Supplementary Fig. 8). Consistent with the early maturation of hippocampal neurons, the wave of repression for regulatory elements was found during mid-gestation for hippocampal inhibitory neurons (Supplementary Fig. 8c,e,f,h). The differentiation of RG to diverse excitatory neurons during mid-gestation is associated with pervasive activation of regulatory elements, as shown by the numerous DMRs that lose mCG (Fig. 5e-g and Supplementary Fig. 9b,g,h). Consistent with the protracted maturation of astrocyte methylome (Fig. 2l,m), the maturation of astrocytes between infant and adult brains is associated with a loss of mCG at 57,783 regions, far exceeding the scale of mCG remodeling in earlier stages of astrocyte differentiation (Supplementary Fig. 7e).

Supplementary Tables

Supplementary Table 1. Human Brain Specimen used in this study.
Supplementary Table 2. snm3C-seq3 metadata
Supplementary Table 3. Cell Type Annotation
Supplementary Table 4. Probes for Chromatin Tracing
Supplementary Table 5. Probes for RNA-MERFISH
Supplementary Table 6. DNA methylation and chromatin conformation features identified in the study.
Supplementary Table 7. Results for overlapping schizophrenia fine-mapped high confidence putative causal loci (PIP > 10%) with DMR / loop / eQTL.
Supplementary Table 8. Sequences of 384-plex RP-H primers.
Supplementary Table 9. RNA MERFISH Codebook
Supplementary Table 10. Antibodies used in Multi-modal DNA/RNA/protein imaging.

Reference for Supplementary Information

1. Luo, C. *et al.* Robust single-cell DNA methylome profiling with snmC-seq2. *Nat. Commun.* **9**, 3824 (2018).
2. Luo, C. *et al.* Single nucleus multi-omics identifies human cortical cell regulatory genome diversity. *Cell Genom* **2**, (2022).
3. Clemens, A. W. *et al.* MeCP2 Represses Enhancers through Chromosome Topology-Associated DNA Methylation. *Mol. Cell* **77**, 279–293.e8 (2020).
4. McLaughlin, K. *et al.* DNA Methylation Directs Polycomb-Dependent 3D Genome Re-organization in Naive Pluripotency. *Cell Rep.* **29**, 1974–1985.e6 (2019).
5. Zhou, J. *et al.* Robust single-cell Hi-C clustering by convolution- and random-walk–based imputation. *Proc. Natl. Acad. Sci. U. S. A.* **116**, 14011–14018 (2019).
6. Shin, H. *et al.* TopDom: an efficient and deterministic method for identifying topological domains in genomes. *Nucleic Acids Res.* **44**, e70 (2016).
7. Godbole, G. *et al.* Hierarchical genetic interactions between FOXG1 and LHX2 regulate the formation of the cortical hem in the developing telencephalon. *Development* **145**, (2018).

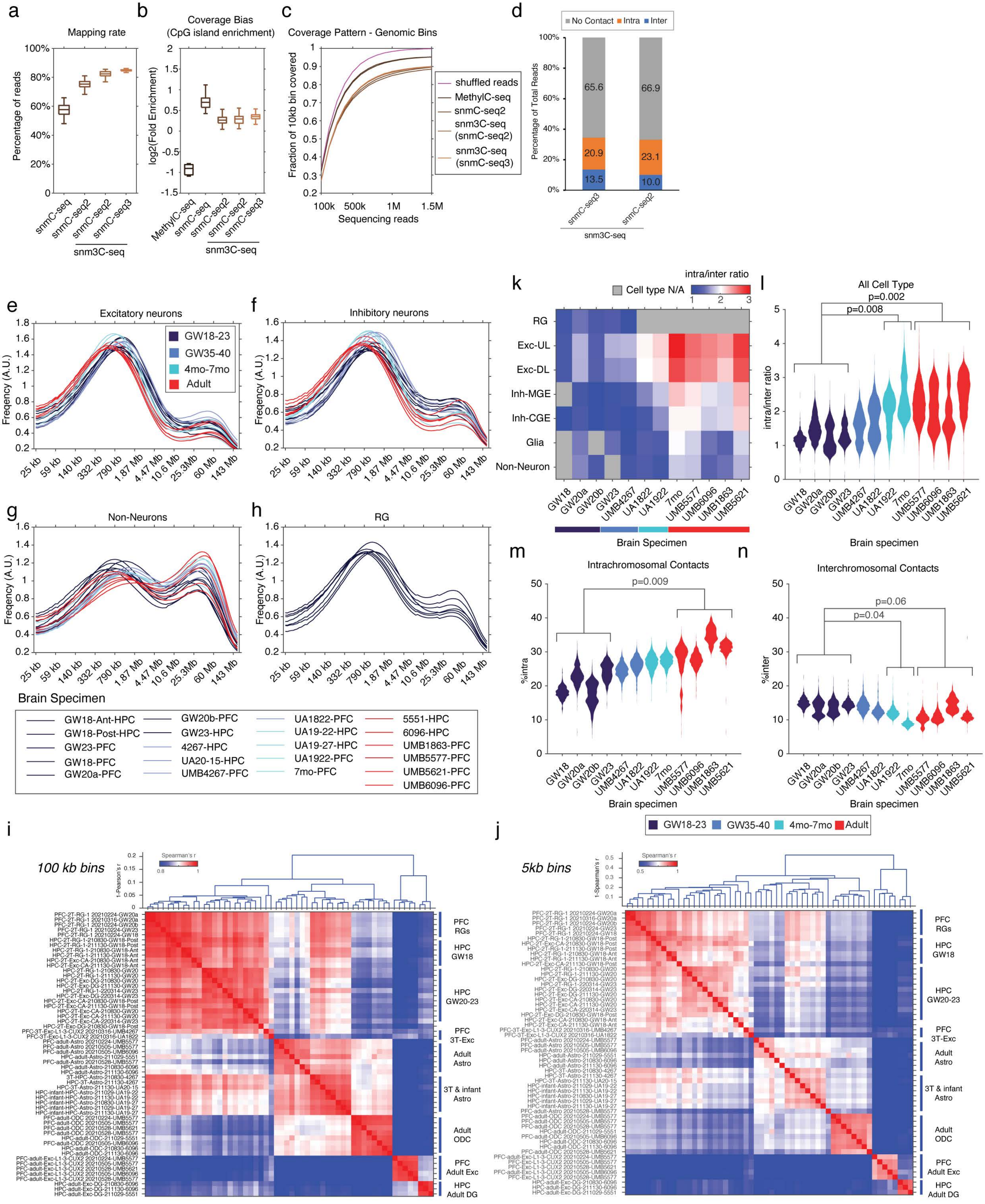

a
Mapping rate
Percentage of reads
100%
80%
60%
40%
20%
0
snmC-seq
snmC-seq2
snmC-seq2
snmC-seq3
snm3C-seq
b
Coverage Bias
(CpG island enrichment)
log2(Fold Enrichment)
MethylC-seq
snmC-seq
snmC-seq2
snmC-seq2
snmC-seq3
snm3C-seq
c
Coverage Pattern - Genomic Bins
Fraction of 10kb bin covered
shuffled reads
MethylC-seq
snmC-seq2
snm3C-seq
(snmC-seq2)
snm3C-seq
(snmC-seq3)
100k
500k
1M
1.5M
Sequencing reads
d
No Contact
Intra
Inter
Percentage of Total Reads
65.6
20.9
13.5
66.9
23.1
10.0
snmC-seq3
snmC-seq2
snm3C-seq
e
Excitatory neurons
f
Inhibitory neurons
g
Non-Neurons
h
RG
Freqency (A.U.)
GW18-23
GW35-40
4mo-7mo
Adult
25 kb
59 kb
140 kb
332 kb
790 kb
1.87 Mb
4.47 Mb
10.6 Mb
25.3Mb
60 Mb
143 Mb
Brain Specimen
GW18-Ant-HPC
GW18-Post-HPC
GW23-PFC
GW18-PFC
GW20a-PFC
GW20b-PFC
GW23-HPC
4267-HPC
UA20-15-HPC
UMB4267-PFC
UA1822-PFC
UA19-22-HPC
UA19-27-HPC
UA1922-PFC
7mo-PFC
5551-HPC
6096-HPC
UMB1863-PFC
UMB5577-PFC
UMB5621-PFC
UMB6096-PFC
k
intra/inter ratio
Cell type N/A
RG
Exc-UL
Exc-DL
Inh-MGE
Inh-CGE
Glia
Non-Neuron
GW18
GW20a
GW20b
GW23
UMB4267
UA1822
UA1922
7mo
UMB5577
UMB6096
UMB1863
UMB5621
Brain Specimen
l
All Cell Type
p=0.002
p=0.008
intra/inter ratio
Brain specimen
m
Intrachromosomal Contacts
p=0.009
%intra
Brain specimen
n
Interchromosomal Contacts
p=0.06
p=0.04
%inter
Brain specimen
GW18-23
GW35-40
4mo-7mo
Adult
i
100 kb bins
1-Pearson's r
Spearman's r
j
5kb bins
1-Spearman's r
PFC RGs
HPC GW18
HPC GW20-23
PFC 3T-Exc
Adult Astro
3T & infant Astro
Adult ODC
PFC Adult Exc
HPC Adult DG

Supplementary Figure 1. Quantity assessment of snm3C-seq3 profiles.
(a-c) snm3C-seq3 methylomes were compared to other single-cell methylome methods with respect to mapping rate (a), enrichment of CpG island (b), and coverage uniformity (c). (d) snm3C-seq libraries generated using snmC-seq3 and snmC-seq2 show similar read types (i.e. no contact, intrachromosomal contact, or interchromosomal contact) composition. (e-h) Distribution of intra-chromosomal chromatin interaction distances for individual brain specimens in excitatory neurons (e), inhibitory neurons (f), non-neuronal cells (g), and radial glia (h). (i-j) Reproducibility of DNA methylome profiles across brain specimen donors and experimental batches computed across 100 kb bins (i) or 5 kb bins (j). (k-n) Correlation between intra/inter- chromosomal interactions and cell types and developmental stages. (k) Developmental and cell-type specific patterns of intra/inter chromosomal interaction ratios. (l) Distribution of intra/inter chromosomal interaction ratios for all cells in each brain specimen. (m) Distribution of %intrachromosomal interaction for excitatory neurons in each brain specimen. (n) Distribution of %interchromosomal interaction for excitatory neurons in each brain specimen.

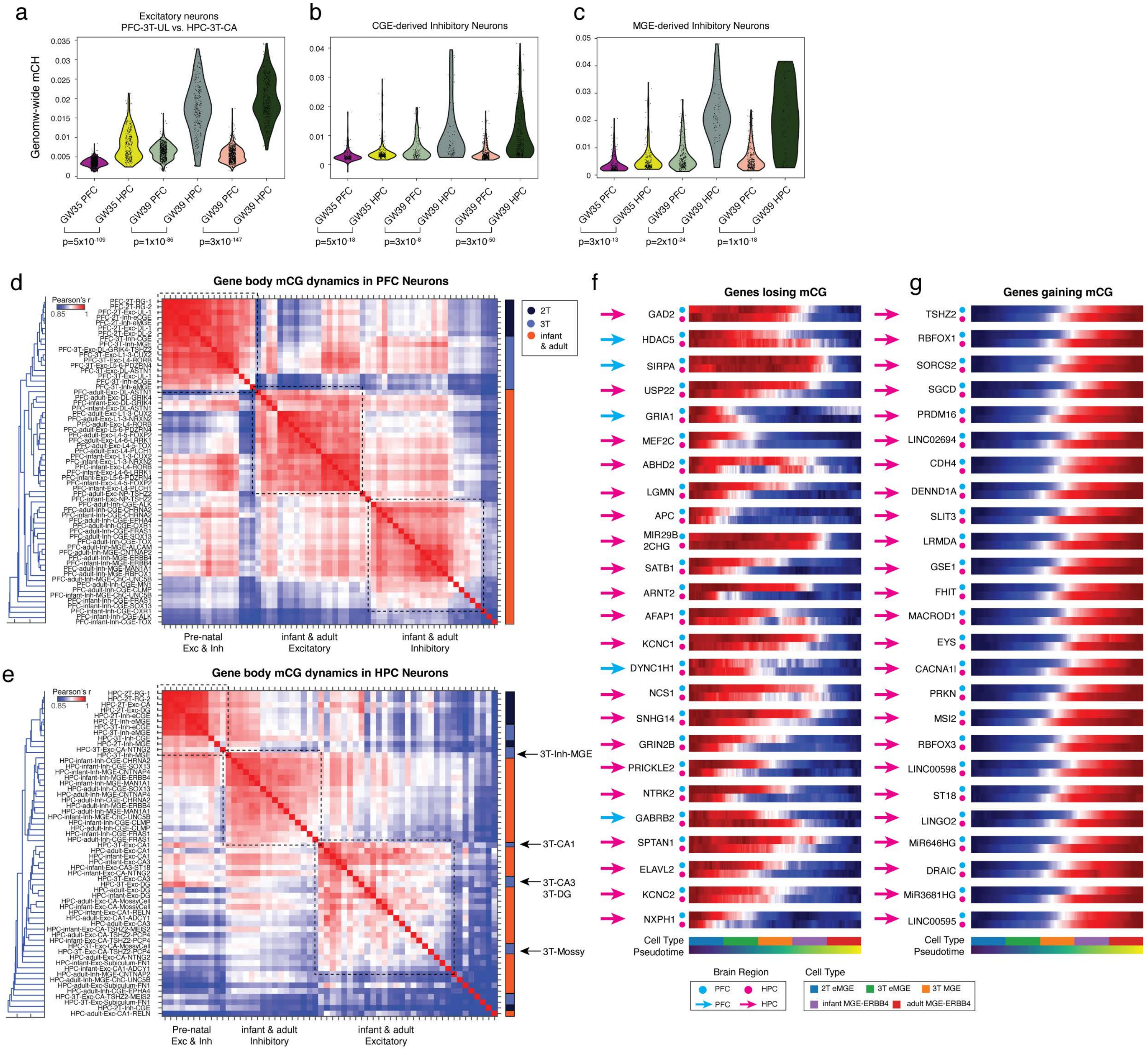

Supplementary Figure 2. Brain regional-specific DNA methylation patterns. (a-c) Comparison of genome-wide mCH levels in cortical and hippocampal excitatory neurons (a), CGE-derived inhibitory neurons (b), and MGE-derived inhibitory neurons (c) in the late-gestational samples. The statistical significance of mCH differences between PFC and HPC was tested using two-sided Wilcoxon Rank Sum tests. (d) Correlation matrix of PFC neuronal populations computed with gene body mCG. (e) Correlation matrix of HPC neuronal populations computed with gene body mCG. (f-g) Comparison of the timing of mCG remodeling in PFC and HPC for genes showing developmental loss of mCG (f) and gain of mCG (g). Arrows in (f) and (g) indicate the brain region showing earlier remodeling of mCG for each gene.

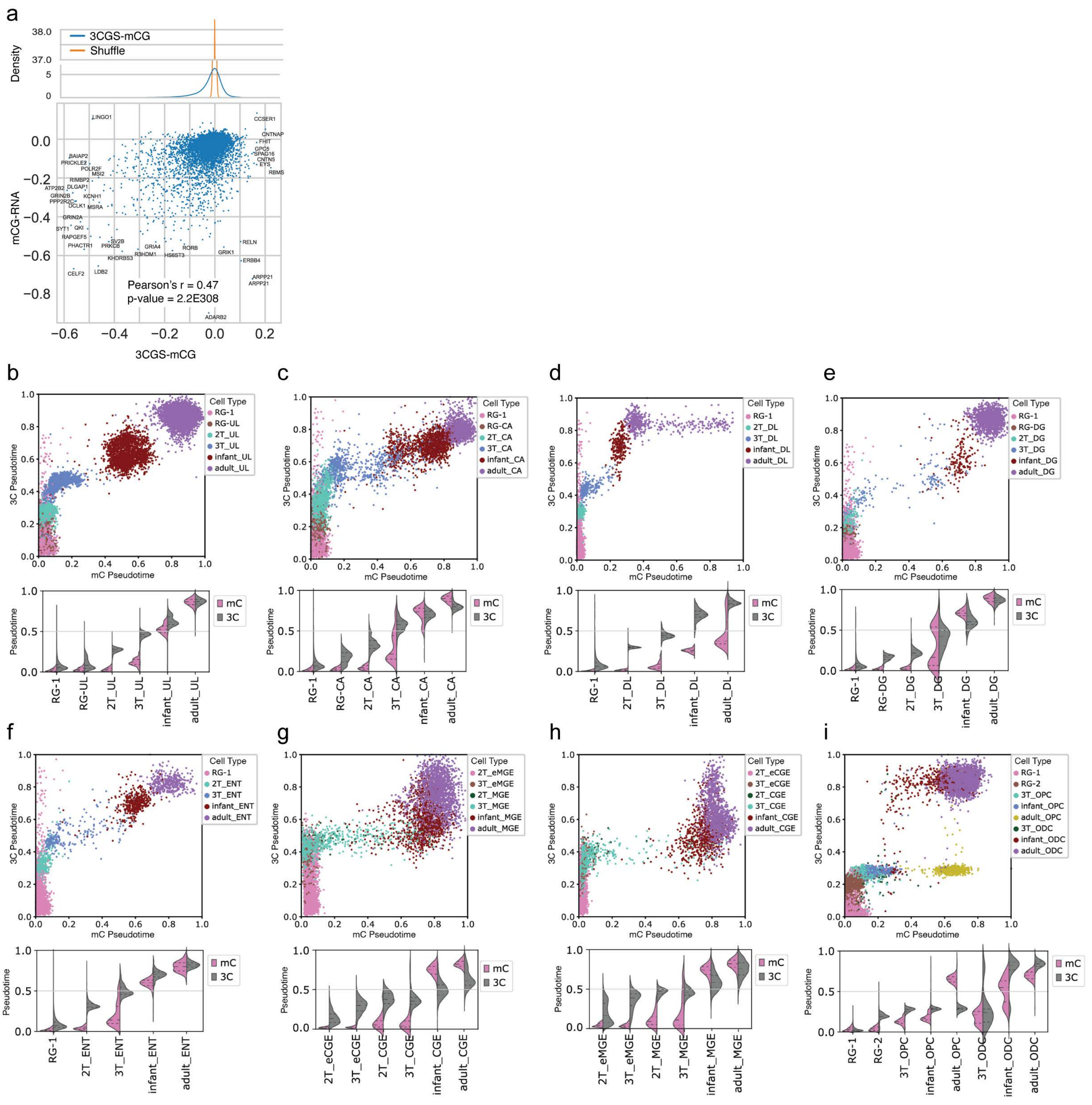

Supplementary Figure 3. Temporal orders of epigenomic remodelings across diverse developmental trajectories. (a) Scatter plot comparing Pearson's coefficients for mCG-RNA and 3CGS-mCG correlations. (b-i) Direct comparison of pseudotime scores computed from mCG or chromatin conformation in individual cells across the differentiation of cortical upper-layer excitatory neurons (b), hippocampal CA excitatory neurons (c), cortical deep-layer excitatory neurons (d), hippocampal DG excitatory neurons (e), entorhinal cortex excitatory neurons (f), MGE-derived inhibitory neurons (g), CGE-derived inhibitory neurons (h), oligodendrocytes and oligodendrocyte progenitors (i).

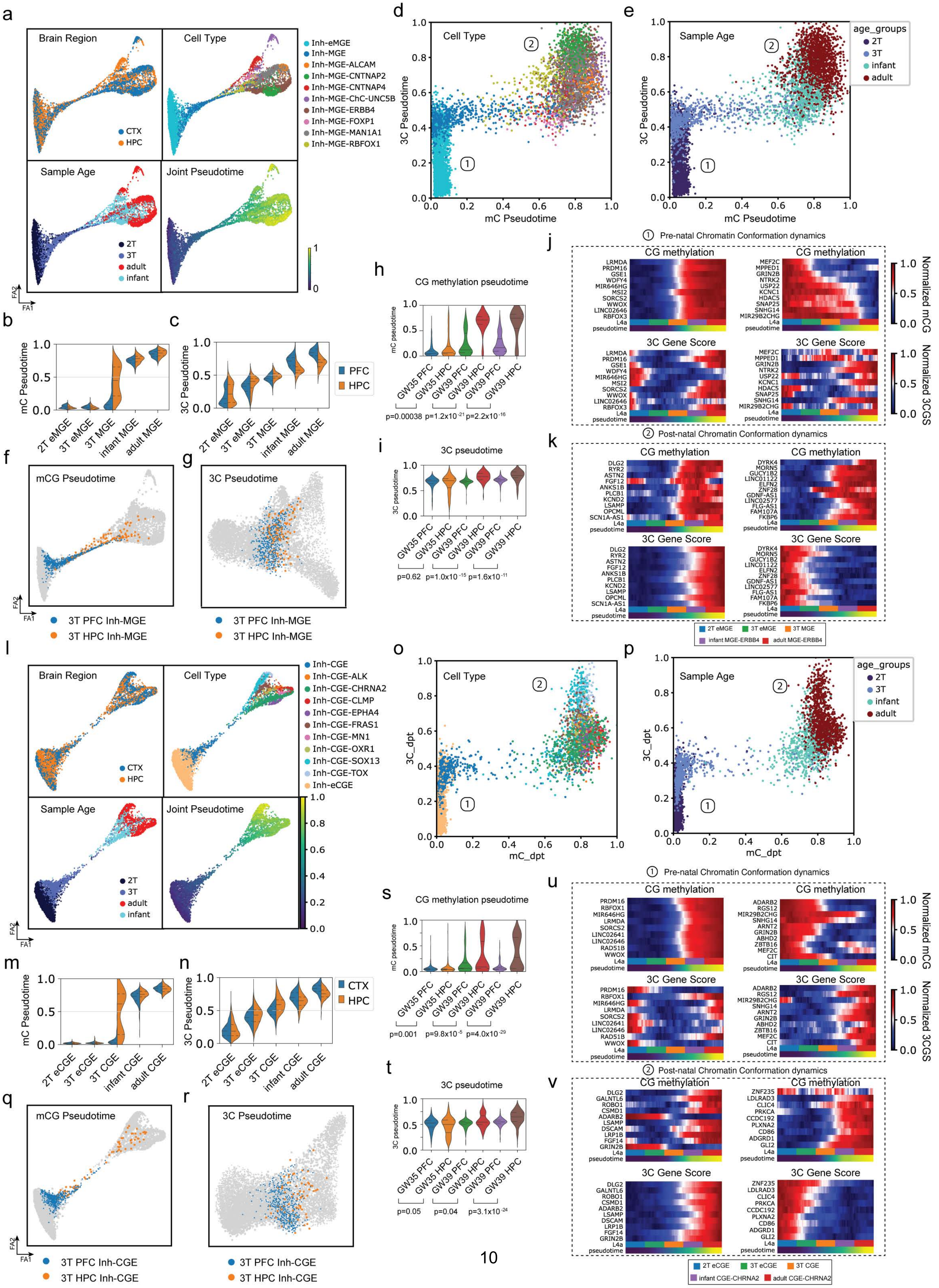

a
Brain Region
CTX
HPC
Cell Type
Inh-eMGE
Inh-MGE
Inh-MGE-ALCAM
Inh-MGE-CNTNAP2
Inh-MGE-CNTNAP4
Inh-MGE-ChC-UNC5B
Inh-MGE-ERBB4
Inh-MGE-FOXP1
Inh-MGE-MAN1A1
Inh-MGE-RBFOX1
Sample Age
2T
3T
adult
infant
Joint Pseudotime
FA1
FA2
d
Cell Type
3C Pseudotime
mC Pseudotime
e
Sample Age
age_groups
2T
3T
infant
adult
b
mC Pseudotime
2T eMGE
3T eMGE
3T MGE
infant MGE
adult MGE
c
3C Pseudotime
PFC
HPC
h
CG methylation pseudotime
mC pseudotime
GW35 PFC
GW35 HPC
GW39 PFC
GW39 HPC
GW39 PFC
GW39 HPC
p=0.00038 p=1.2x10^-21 p=2.2x10^-16
i
3C pseudotime
p=0.62 p=1.0x10^-15 p=1.6x10^-11
j
① Pre-natal Chromatin Conformation dynamics
CG methylation
3C Gene Score
Normalized mCG
Normalized 3CGS
LRMDA
PRDM16
GSE1
WDFY4
MIR646HG
MSI2
SORCS2
WWOX
LINC02646
RBFOX3
MEF2C
MPPED1
GRIN2B
NTRK2
USP22
KCNC1
HDAC5
SNAP25
SNHG14
MIR29B2CHG
L4a
pseudotime
k
② Post-natal Chromatin Conformation dynamics
DLG2
RYR2
ASTN2
FGF12
ANKS1B
PLCB1
KCND2
LSAMP
OPCML
SCN1A-AS1
DYRK4
MORN5
GUCY1B2
LINC01122
ELFN2
ZNF28
GDNF-AS1
LINC02577
FLG-AS1
FAM107A
FKBP6
2T eMGE
3T eMGE
3T MGE
infant MGE-ERBB4
adult MGE-ERBB4
f
mCG Pseudotime
3T PFC Inh-MGE
3T HPC Inh-MGE
g
3C Pseudotime
l
Inh-CGE
Inh-CGE-ALK
Inh-CGE-CHRNA2
Inh-CGE-CLMP
Inh-CGE-EPHA4
Inh-CGE-FRAS1
Inh-CGE-MN1
Inh-CGE-OXR1
Inh-CGE-SOX13
Inh-CGE-TOX
Inh-eCGE
o
3C_dpt
mC_dpt
p
m
2T eCGE
3T eCGE
3T CGE
infant CGE
adult CGE
n
CTX
HPC
s
p=0.001 p=9.8x10^-5 p=4.0x10^-29
t
p=0.05 p=0.04 p=3.1x10^-24
u
PRDM16
RBFOX1
MIR646HG
LRMDA
SORCS2
LINC02641
LINC02646
RAD51B
WWOX
ADARB2
RGS12
MIR29B2CHG
SNHG14
ARNT2
GRIN2B
ABHD2
ZBTB16
MEF2C
CIT
v
DLG2
GALNTL6
ROBO1
CSMD1
ADARB2
LSAMP
DSCAM
LRP1B
FGF14
GRIN2B
ZNF235
LDLRAD3
CLIC4
PRKCA
CCDC192
PLXNA2
CD86
ADGRD1
GLI2
2T eCGE
3T eCGE
3T CGE
infant CGE-CHRNA2
adult CGE-CHRNA2
q
3T PFC Inh-CGE
3T HPC Inh-CGE
r

Supplementary Figure 4. Temporal ordering of DNA methylation and chromatin conformation reconfiguration during the maturation of MGE- and CGE- derived inhibitory neurons. (a) UMAP dimensionality reduction of MGE-derived inhibitory neurons. The UMAP was labeled by brain region, cell type, sample age, and joint pseudotime scores computed using the fusion of DNA methylation and chromatin confirmation information. (b-c) Distribution of pseudotime scores computed from gene body mCG (b) or interaction frequency of genomic bin pairs (c) in MGE-derived inhibitory neurons. Blue and orange colors each indicate PFC and HPC, respectively. The statistical significance was tested using two-sided Wilcoxon Rank Sum tests. (d-e) Direct comparison of pseudotime scores computed from mCG or chromatin conformation in individual cells, labeled by cell types (d) or developmental age groups (e). (f-g) Comparison of mCG (f) and chromatin conformation (g) pseudotime scores between PFC and HPC in late-gestational MGE-derived inhibitory cells. (h-i) Quantitative comparison of mCG (h) and chromatin conformation (i) pseudotime scores between PFC and HPC in late-gestational MGE-derived inhibitory cells. The statistical significance was tested using two-sided Wilcoxon Rank Sum tests. (j) mCG and 3C gene scores change at loci associated with pre-natal chromatin conformation dynamics. (k) mCG and 3C gene scores change at loci associated with post-natal chromatin conformation dynamics. (l) UMAP dimensionality reduction of CGE-derived inhibitory neurons. The UMAP was labeled by brain region, cell type, sample age, and joint pseudotime scores computed using the fusion of DNA methylation and chromatin confirmation information. (m-n) Distribution of pseudotime scores computed from gene body mCG (m) or interaction frequency of genomic bin pairs (n) in MGE-derived inhibitory neurons. Blue and orange colors each indicate PFC and HPC, respectively. The statistical significance was tested using two-sided Wilcoxon Rank Sum tests. (o-p) Direct comparison of pseudotime scores computed from mCG or chromatin conformation in individual cells, labeled by cell types (o) or developmental age groups (p). (q-r) Comparison of mCG (q) and chromatin conformation (r) pseudotime scores between PFC and HPC in late-gestational MGE-derived inhibitory cells. (s-t) Quantitative comparison of mCG (s) and chromatin conformation (t) pseudotime scores between PFC and HPC in late-gestational MGE-derived inhibitory cells. The statistical significance was tested using two-sided Wilcoxon Rank Sum tests. (u) mCG and 3C gene scores change at loci associated with pre-natal chromatin conformation dynamics. (v) mCG and 3C gene scores change at loci associated with post-natal chromatin conformation dynamics.

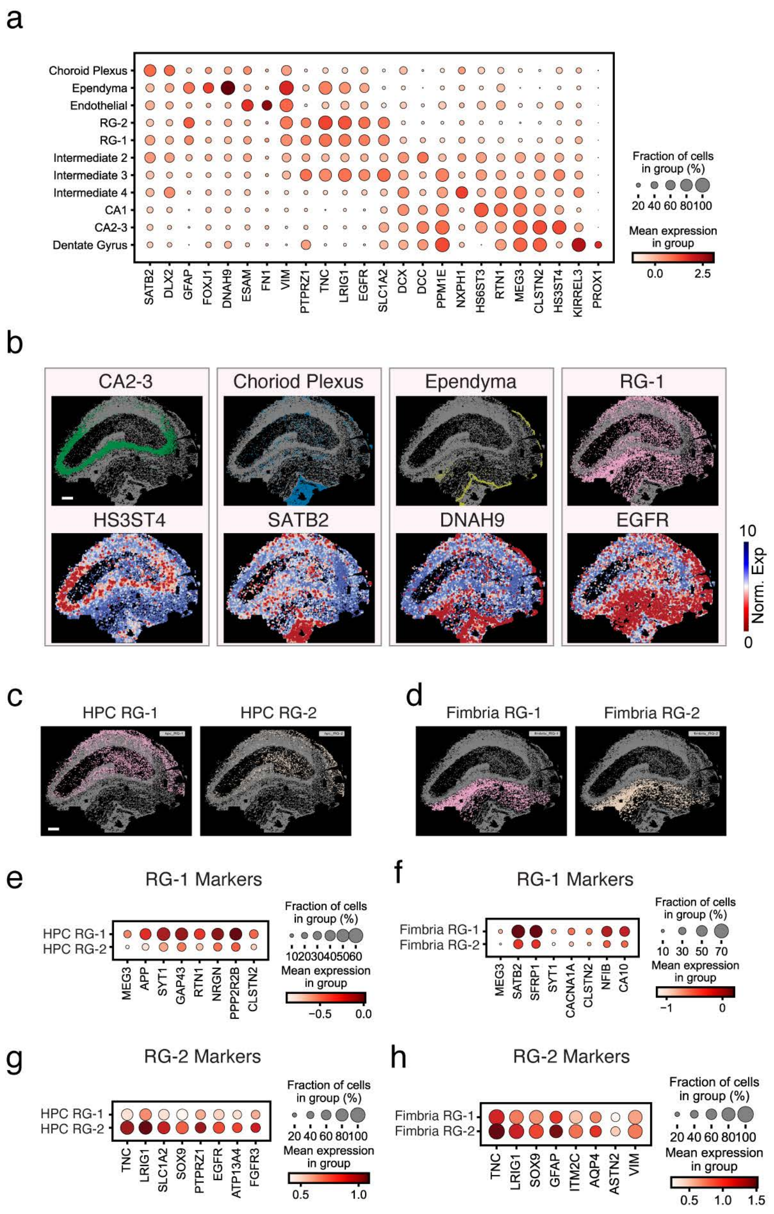

Supplementary Figure 5. Identification of brain cell types using RNA-MERFISH profiles. (a) Marker gene expression quantified by RNA-MERFISH for brain cell types identified in the hippocampus, fimbria, and choroid plexus structures. (b) Spatial localization of annotated cell types and marker gene expressions. The scale bar indicates 250 μm. (c-d) The spatial location of RG-1 and RG-2 populations in HPC (c) and fimbria (d). (e-f) Specificity of RG-1 marker expressions in HPC in RG-1 cells located in HPC (e) or fimbria (f). (g-h) Specificity of RG-2 marker expressions in HPC in RG-2 cells located in HPC (g) or fimbria (h).

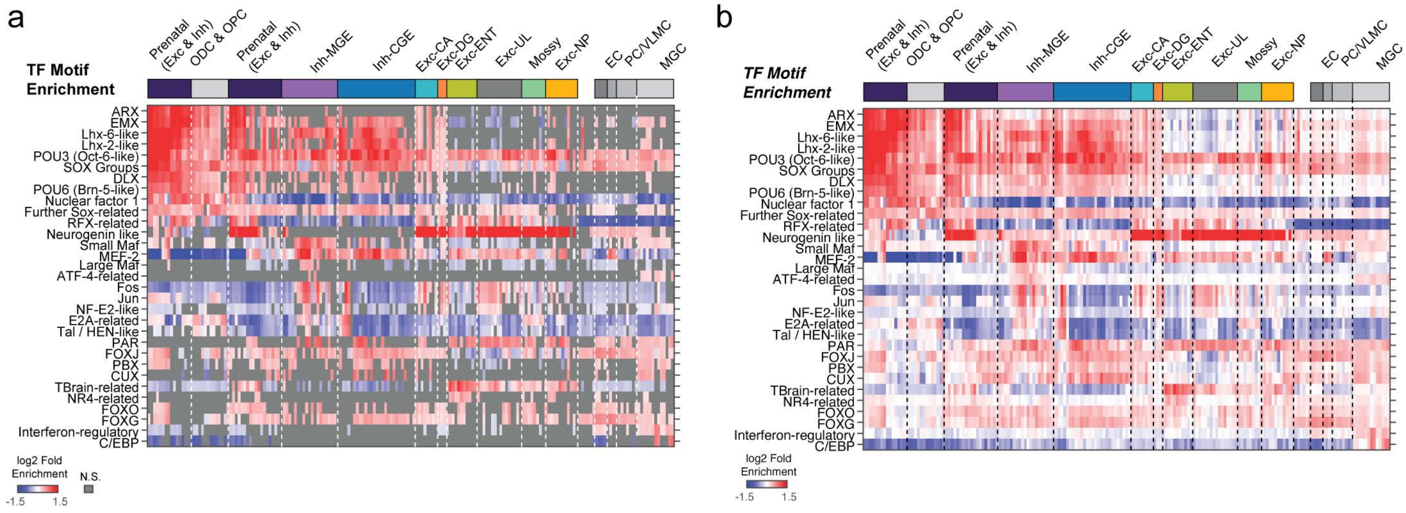

Supplementary Figure 6. TF binding motif analysis of DMRs. (a) Transcription binding motif enrichment analysis of DMRs with the headmap showing log2(fold enrichment/depletion). Enriched and depleted values were shown in red and blue, respectively. Statistically insignificant values (FDR > 1x10-5) were shown as gray. (b) Transcription binding motif enrichment analysis of DMRs, including statistically insignificant values (FDR > 1x10-5).

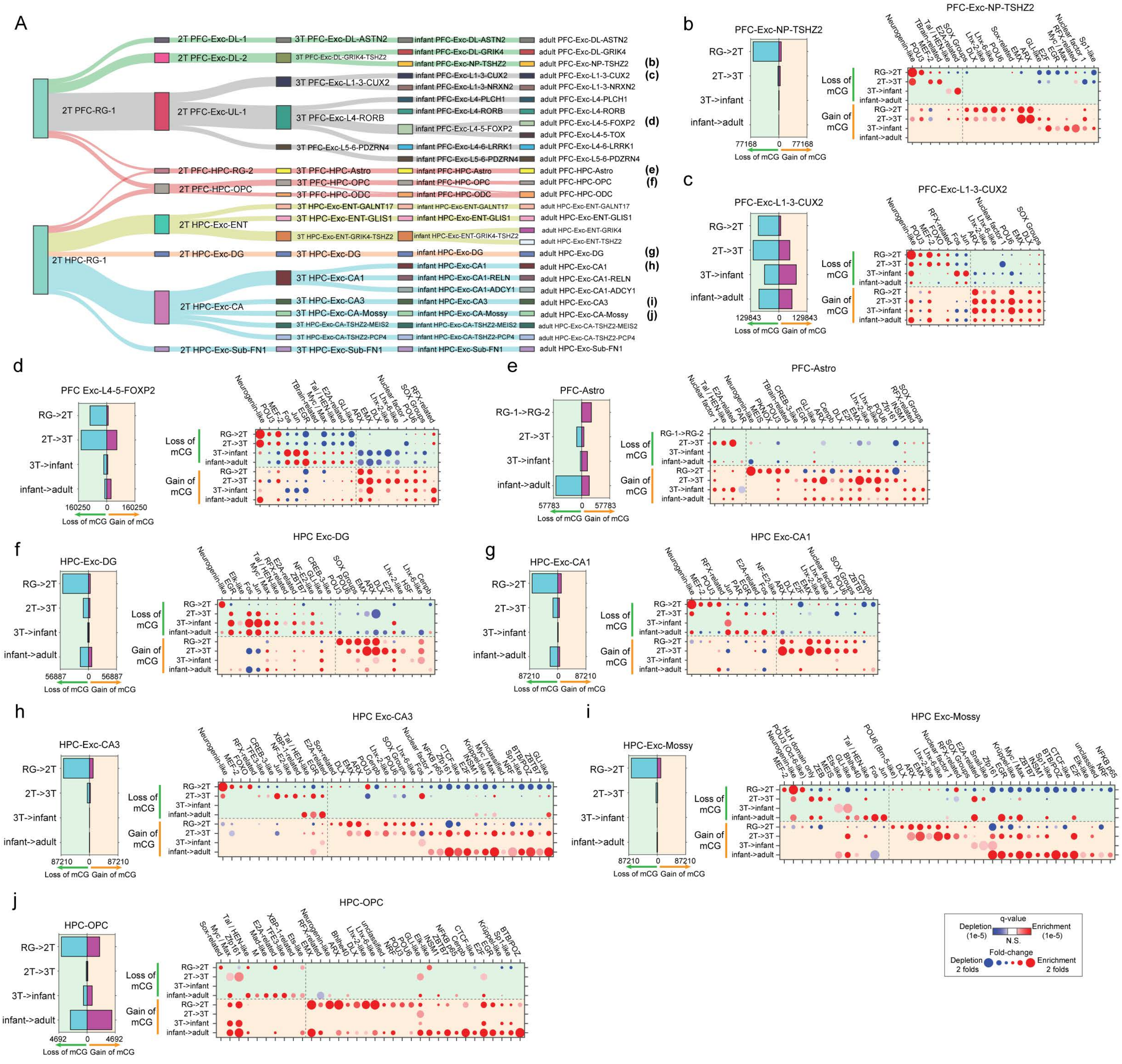

Supplementary Figure 7. Identification of trajectory-DMRs across excitatory neuron and glial cell differentiation trajectories. (a) Reconstructed developmental hierarchy of excitatory neurons and glial cells. (b-j) The number of trajectory-DMRs identified between developmental stages of several cell-type trajectories (left). Transcription factor binding motif enrichments between developmental stages of each trajectory. Displayed trajectories are PFC-Exc-NP-TSHZ2 (b), PFC-Exc-L1-2-CUX2 (c), PFC-Exc-L4-5-FOXP2 (d), PFC Astro (e), HPC-OPC (f), HPC-Exc-DG (g), HPC-Exc-CA1 (h), HPC-Exc-CA3 (i), and HPC-Exc-Mossy (j).

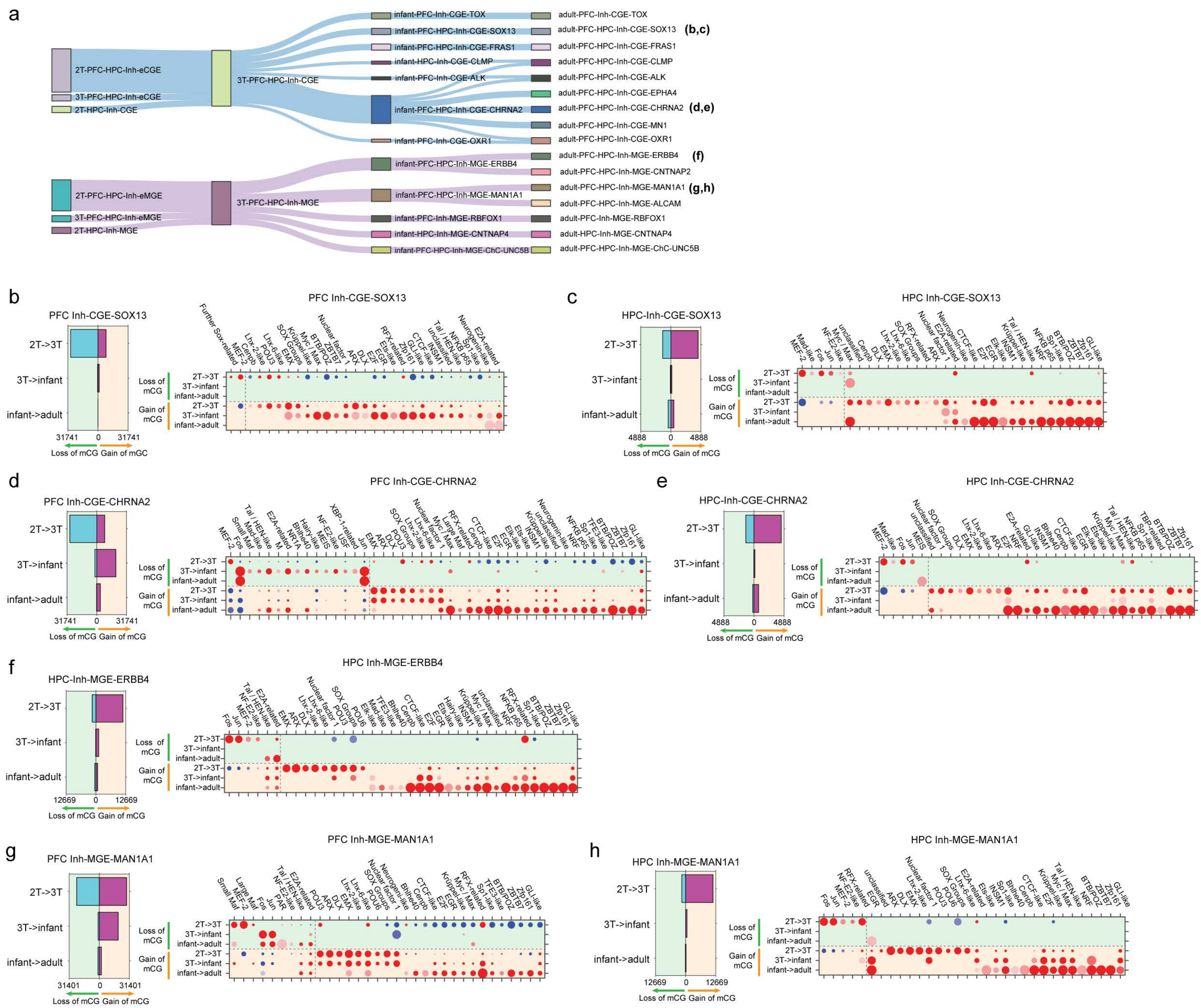

Supplementary Figure 8. Identification of trajectory-DMRs across inhibitory neuron differentiation trajectories. (a) Reconstructed developmental hierarchy of CGE- and MGE-derived inhibitory neurons. (b-h) Trajectory-DMRs identified between developmental stages of cell-type trajectories (left). Transcription factor binding motif enrichments between developmental stages of the trajectory. Displayed trajectories are PFC-Inh-CGE-SOX13 (b), HPC-Inh-CGE-SOX13 (c), PFC-Inh-CGE-CHRNA2 (d), HPC-Inh-CGE-CHRNA2 (e), HPC-Inh-MGE-ERBB4 (f), PFC-Inh-MGE-MAN1A1 (g), HPC-Inh-MGE-MAN1A1 (h).

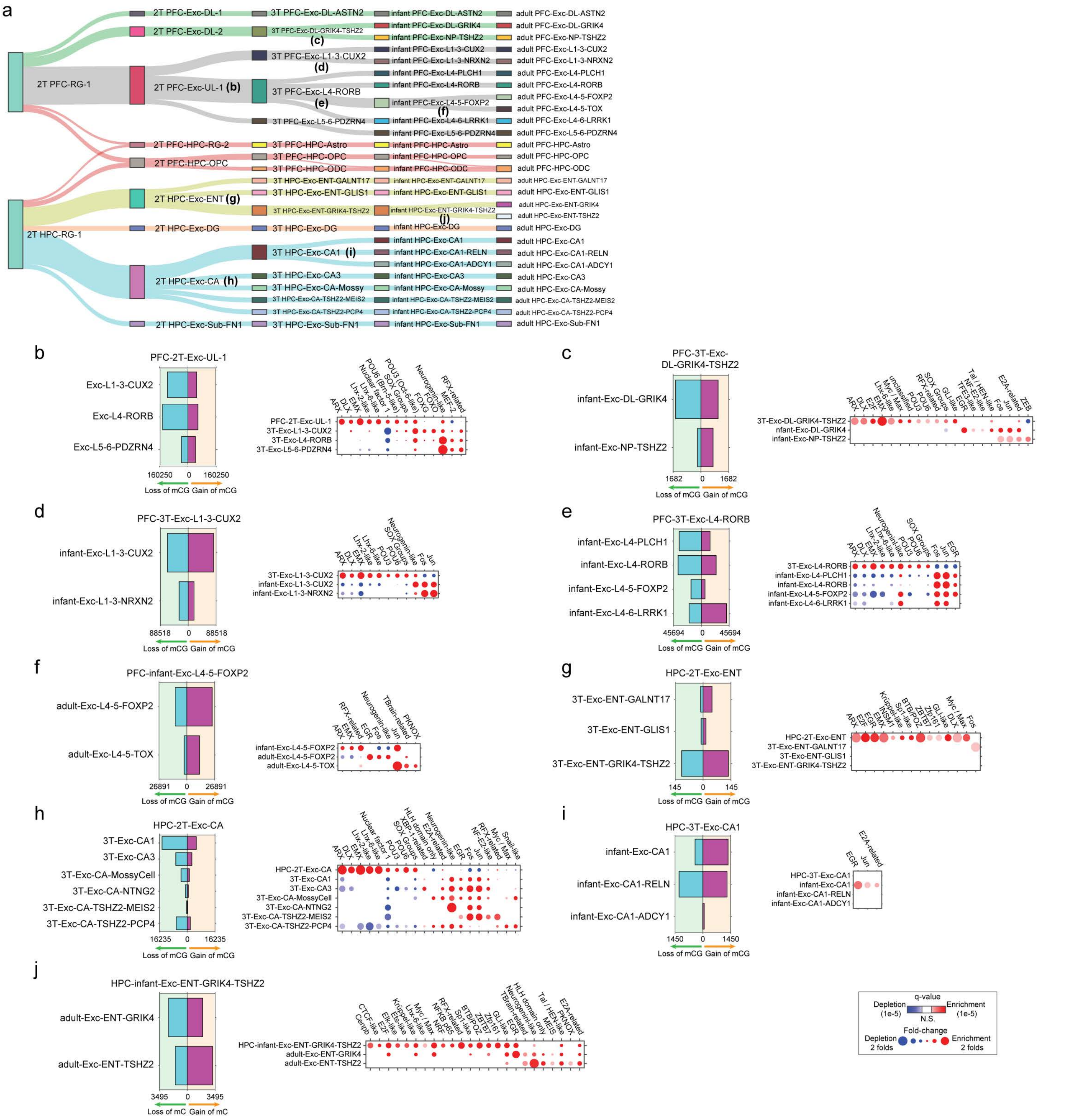

Supplementary Figure 9. Identification of branch-DMRs across excitatory neuron differentiation trajectories. (a) Reconstructed developmental hierarchy of excitatory neurons and glial cells labeled for the branching of a mother cell type in an earlier developmental stage to daughter cell types in a later development stage. (b-j) The number of hypo-methylated branch-DMRs identified at cell-type branches and transcription factor binding motif enrichments in DMRs. Displayed cell-type branches are associated with mother cell populations PFC-2T-Exc-UL-1 (b), PFC-3T-Exc-DL-GRIK4-TSHZ2 (c), PFC-3T-Exc-L1-3-CUX2 (d), PFC-3T-Exc-L4-RORB (e), PFC-infant-Exc-L4-5-FOXP2 (f), HPC-2T-Exc-ENT (g), HPC-2T-Exc-CA (h), HPC-3T-Exc-CA1 (i) and HPC-infant-Exc-ENT-GRIK4-TSHZ2 (j).

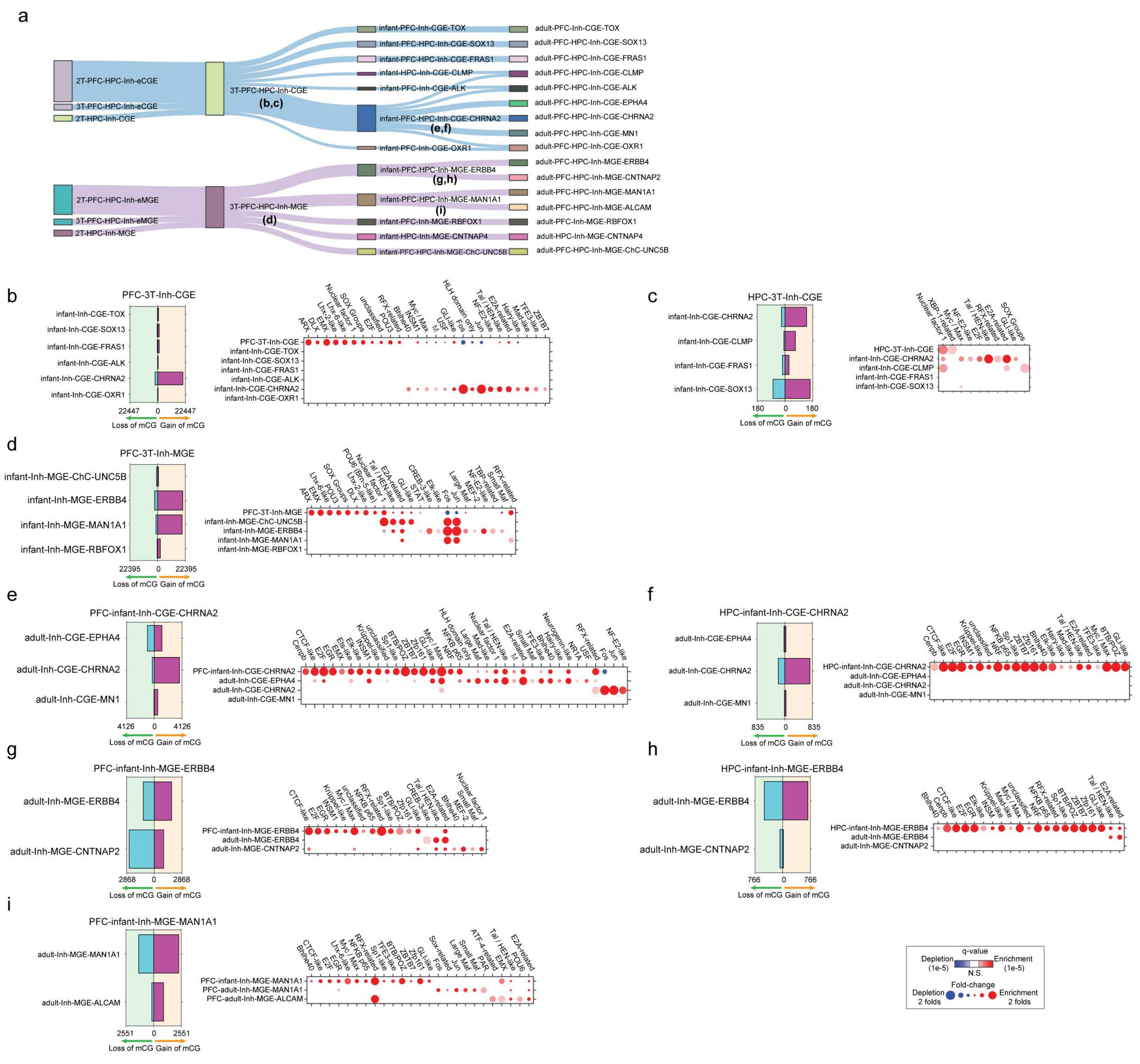

Supplementary Figure 10. Identification of branch-DMRs across inhibitory neuron differentiation trajectories. (a) Reconstructed developmental hierarchy of inhibitory neurons labeled for the branching of a mother cell type in an earlier developmental stage to daughter cell types in a later development stage. (b-i) The number of hypo-methylated branch-DMRs identified at cell-type branches and transcription factor binding motif enrichments in DMRs. Displayed cell-type branches are associated with mother cell populations PFC-3T-Inh-CGE (b), HPC-3T-Inh-CGE (c), PFC-3T-Inh-MGE (d), PFC-infant-Inh-CGE-CHRNA2 (e), HPC-infant-Inh-CGE-CHRNA2 (f), PFC-infant-Inh-MGE-ERBB4 (g), HPC-infant-Inh-MGE-ERBB4 (h), PFC-infant-Inh-MGE-MAN1A1 (i).

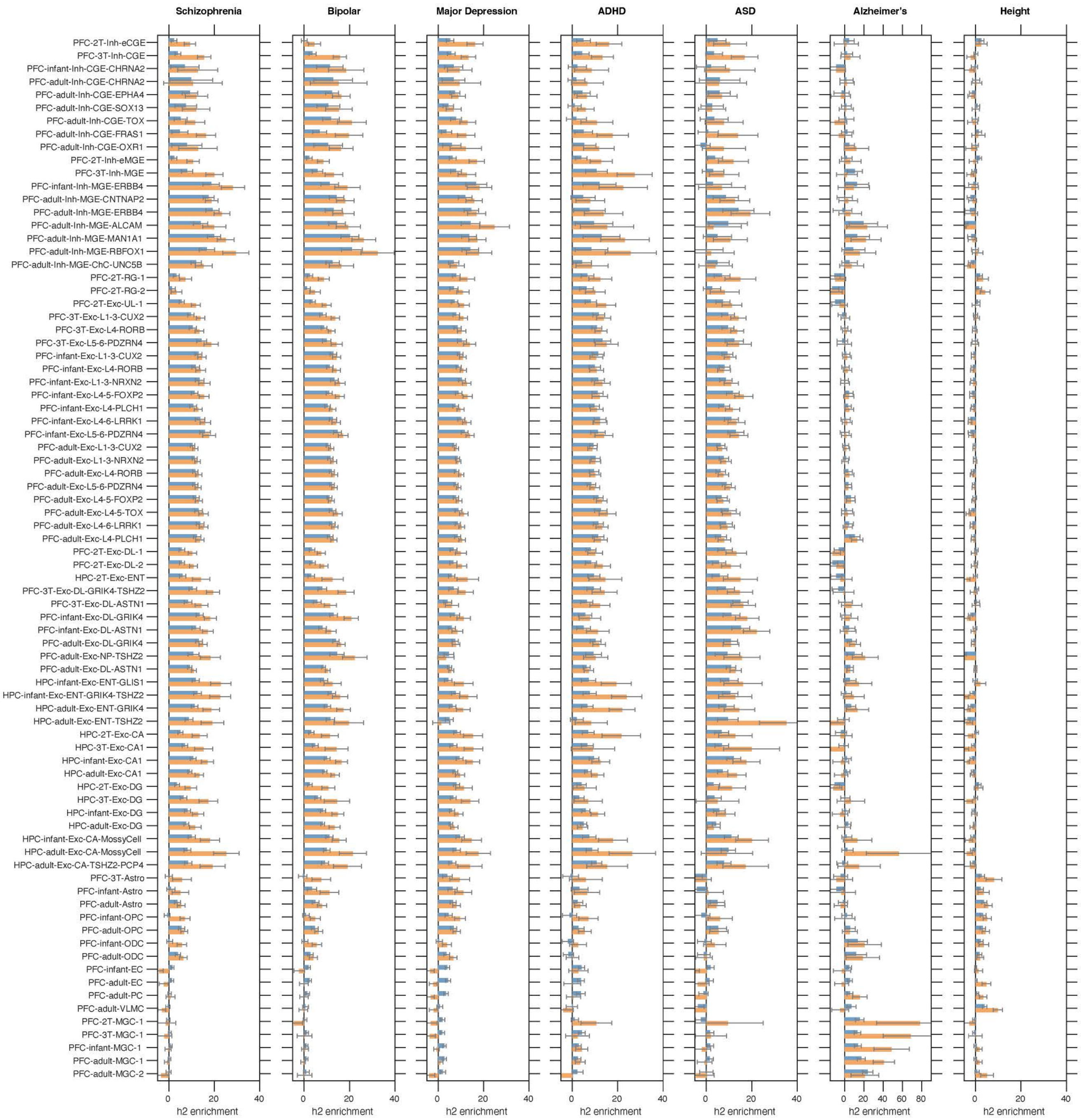

Supplementary Figure 11. Enrichment of polygenic heritability for seven traits in DMRs and loop-connected DMRs. Blue and orange bars indicate DMRs and loop-connected DMRs, respectively.

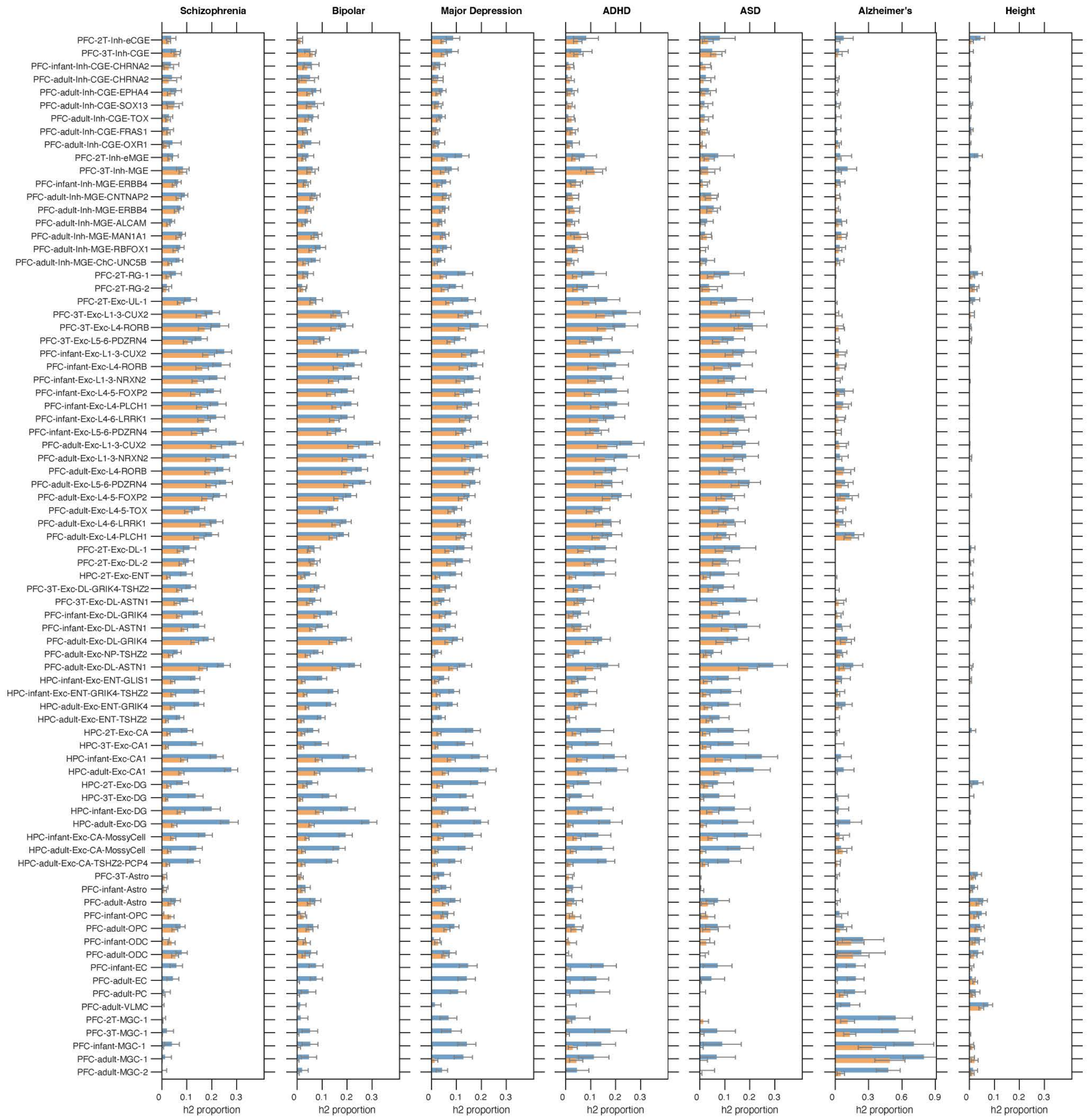

Supplementary Figure 12. Proportions of polygenic heritability for seven traits in DMRs and loop-connected DMRs across cell types. Blue and orange bars indicate DMRs and loop-connected DMRs, respectively.
