# Supplementary material for: Temporally distinct 3D multi-omic dynamics in the developing human brain
Source: Nature. 2024 Oct 9;635(8038):481–9. doi: 10.1038/s41586-024-08030-7 (PMC11560841; doi:10.1038/s41586-024-08030-7)
Supplement: Supplementary file 2 — Reporting Summary [file 41586_2024_8030_MOESM2_ESM.pdf]

nature portfolio

Corresponding author(s): Chongyuan Luo, Mercedes Paredes

Last updated by author(s): Jul 30, 2024

Reporting Summary

Nature Portfolio wishes to improve the reproducibility of the work that we publish. This form provides structure for consistency and transparency in reporting. For further information on Nature Portfolio policies, see our Editorial Policies and the Editorial Policy Checklist.

Statistics

For all statistical analyses, confirm that the following items are present in the figure legend, table legend, main text, or Methods section.

n/a	Confirmed	
☐	☒	The exact sample size (*n*) for each experimental group/condition, given as a discrete number and unit of measurement
☐	☒	A statement on whether measurements were taken from distinct samples or whether the same sample was measured repeatedly
☐	☒	The statistical test(s) used AND whether they are one- or two-sided<br>*Only common tests should be described solely by name; describe more complex techniques in the Methods section.*
☐	☒	A description of all covariates tested
☐	☒	A description of any assumptions or corrections, such as tests of normality and adjustment for multiple comparisons
☐	☒	A full description of the statistical parameters including central tendency (e.g. means) or other basic estimates (e.g. regression coefficient) AND variation (e.g. standard deviation) or associated estimates of uncertainty (e.g. confidence intervals)
☐	☒	For null hypothesis testing, the test statistic (e.g. *F*, *t*, *r*) with confidence intervals, effect sizes, degrees of freedom and *P* value noted<br>*Give P values as exact values whenever suitable.*
☒	☐	For Bayesian analysis, information on the choice of priors and Markov chain Monte Carlo settings
☒	☐	For hierarchical and complex designs, identification of the appropriate level for tests and full reporting of outcomes
☐	☒	Estimates of effect sizes (e.g. Cohen's *d*, Pearson's *r*), indicating how they were calculated

Our web collection on statistics for biologists contains articles on many of the points above.

Software and code

Policy information about availability of computer code

Data collection	The demultiplexing of sequencing runs was performed using the Illumina DRAGEN pipeline implemented on BaseSpace
Data analysis	Codes for the demultiplexing of sn-m3C-seq fastq files are available at https://github.com/luogenomics/demultiplexing. Modified TAURUS-MH (version 0.1) for mapping of sn-m3C-seq data is available at https://github.com/luogenomics/Taurus-MH. Codes for the generation and imputation of methylation features is available at https://github.com/luogenomics/snm3Cseq_feature_processing. Custom code used for analyzing chromatin tracing and MERFISH datasets in this study are available here: https://github.com/cfg00/MERFISH_Chromatin_Tracing_2024.

For manuscripts utilizing custom algorithms or software that are central to the research but not yet described in published literature, software must be made available to editors and reviewers. We strongly encourage code deposition in a community repository (e.g. GitHub). See the Nature Portfolio guidelines for submitting code & software for further information.

Data

Policy information about availability of data

All manuscripts must include a data availability statement. This statement should provide the following information, where applicable:

- Accession codes, unique identifiers, or web links for publicly available datasets
- A description of any restrictions on data availability
- For clinical datasets or third party data, please ensure that the statement adheres to our policy

Datasets generated by this study can be accessed interactively through https://brain-epigenome.cells.ucsc.edu/ and https://genome.ucsc.edu/s/luogenomics/hs-brain-epigenome. Processed chromatin conformation data for all samples, processed single-cell DNA methylation data for unrestricted access samples, and processed multi-modal imaging data can be downloaded at NCBI GEO accession GSE213950. Raw sequencing reads for all samples and processed DNA methylation data for controlled access samples can be downloaded from the NeMO Archive (https://assets.nemoarchive.org/dat-obec38w). Raw data for prenatal specimens analyzed in this study requires controlled access as specified in the consent for tissue donation. Requests for controlled data hosted by NeMO Archive can be made through the National Institute of Mental Health (NIMH) Data Archive (NDA) (https://nda.nih.gov/). Controlled data associated with this study is permitted for General Research Use (GRU). Instructions for requesting access to controlled data hosted by NeMO are provided at https://nemoarchive.org/resources/accessing-controlled-access-data. Single-cell RNA-seq data for prenatal human cortical specimens were published in ref. 22. Single-nucleus ATAC-seq data for prenatal human cortical specimens were published in ref. 14. Bulk Hi-C data for multiple human tissues was published in ref. 29. Bulk Hi-C data for neuronal and non-neuronal nuclei isolated adult human brains was published in ref. 30. BRAINSPAN developmental transcriptome dataset was published in ref. 11. GWAS summary statistics included Schizophrenia 41, Bipolar disorder 63, Major Depressive Disorder 64, ADHD 65, ASD 66, Alzheimer's disease 67, Height GWAS in UK Biobank 68 (downloaded from https://alkesgroup.broadinstitute.org/sumstats_formatted/).

Human research participants

Policy information about studies involving human research participants and Sex and Gender in Research.

Reporting on sex and gender	Our study includes no human subject. De-identified Prenatal and post-mortem human tissues were used in the study and were not considered as human subjects as determined by IRBs at UCLA ad UCSF.
Population characteristics	The prefrontal cortex dataset included four mid-gestational donors (GW18-23), two late-gestational donors (GW35-29), two infant donors (4-7 months) and five adult donors (21-37 yrs old). The hippocampus dataset included 3 mid-gestational donors (GW18-23), two late-gestational donors (GW35-39), two infant donors (4-7 months) and two adult donors (29-55 yrs old).
Recruitment	No Recruitment involved.
Ethics oversight	Cases obtained from UCSF were collected from autopsy sources via the UCSF Pediatric Neuropathology Research Laboratory (PNRL) and gynecology clinic. Patients were asked about their interest in donating the aborted tissue to research (Table S1). Patients that agreed, signed a written consent after receiving information, both written and oral, given by a physician or midwife. Age (post-conception) of the embryos and fetuses was estimated using clinical information (last menstrual period, ultrasound), true crown-rump-length and anatomical landmarks. The use of abortion material was reviewed and approved by the University of California, San Francisco (UCSF) Committee on Human Research. Protocols were approved by the Human Gamete, Embryo and Stem Cell Research Committee (Institutional Review Board GESCR# 10-02693) at UCSF. Samples collected through autopsy were de-identified before acquisition and thus exempt from IRB review.<br>Adult human brain samples and a post-mortem GW35 sample (based on adjusted age) were banked by NIH NeuroBioBank at the University of Maryland Brain and Tissue Bank (Table S1). The tissue collection and repository is overseen by The University of Maryland Institutional Review Board (IRB) with IRB Protocol Number HM-HP-00042077, as well as The Maryland Department of Health and Mental Hygiene IRB with IRB Protocol Number 5-58. When an individual of any age dies, the Medical Examiner or Coroner contacts the next of kin and asks if they would be willing to talk to a staff member at the University of Maryland Bank and Tissue Bank about an NIH-funded tissue procurement project. If the family agrees, the Medical Examiner or Coroner contacts the Bank and a staff member will obtain a recorded telephone consent from the next of kin for donation. A written verification of the consent is then faxed to the referring Medical Examiner's or Coroner's office. Alternatively, the recording can also be played over the telephone for confirmation. University of California Los Angeles (UCLA) IRB has determined our study using post-mortem human tissue obtained from NIH NeuroBioBank involves no human subject and requires no IRB review.

Note that full information on the approval of the study protocol must also be provided in the manuscript.

Field-specific reporting

Please select the one below that is the best fit for your research. If you are not sure, read the appropriate sections before making your selection.

☒ Life sciences ☐ Behavioural & social sciences ☐ Ecological, evolutionary & environmental sciences

For a reference copy of the document with all sections, see nature.com/documents/nr-reporting-summary-flat.pdf

Life sciences study design

All studies must disclose on these points even when the disclosure is negative.

Sample size	The sample size (n>=3) for each age group was determined based on our previous studies (e.g. Luo et al., 2022 Cell Genomics) showing brain cell types identified using single-cell methylation signatures are highly consistent between donors of similar age. Therefore n>=3 for each age group is sufficient for robust classifications of cell types. We analyzed n=14 frontal cortex samples and n=11 hippocampus samples. In spite of the challenge associated with acquiring pre-natal and post-mortem developmental brain samples, we aimed to include at least 3 cases for each developmental stage (i.e. mid-gestation, late-gestation, infancy and adulthood) including both male and female samples
Data exclusions	As described in the method section, cells were filtered on the basis of several metadata metrics: (1) mCCC level <0.03; (2) global mCG level >0.5; (3) global mCH level < 0.2; and (4) Total chromatin interactions >100,000.
Replication	Multiple cases in each developmental stage were always analyzed independently and serve as biological replicates.
Randomization	Randomization was not used in this study. This study does not involve any case/control design but instead compared molecular differences between samples of different ages. Therefore randomization was not applicable.
Blinding	Investigators were not blinked in this study as quantitative measures were used to measure our results. The cell types were determined using unbiased and unsupervised clustering approaches and thus any potential human bias has little impact on the reported results.

Reporting for specific materials, systems and methods

We require information from authors about some types of materials, experimental systems and methods used in many studies. Here, indicate whether each material, system or method listed is relevant to your study. If you are not sure if a list item applies to your research, read the appropriate section before selecting a response.

Materials & experimental systems

n/a	Involved in the study	
☐	☒	Antibodies
☒	☐	Eukaryotic cell lines
☒	☐	Palaeontology and archaeology
☒	☐	Animals and other organisms
☒	☐	Clinical data
☒	☐	Dual use research of concern

Methods

n/a	Involved in the study	
☒	☐	ChIP-seq
☒	☐	Flow cytometry
☒	☐	MRI-based neuroimaging

Antibodies

Antibodies used	anti-NeuN antibody (PE-conjugated, clone A60, Millipore-Sigma #FCMAB317PE)<br>Mouse Histone H3 trimethylated at lysine 9 (H3K9me3) (Diagenode, Cat# C15200146, RRID:AB_2927650)<br>Rabbit Anti-RNA polymerase II CTD repeat YSPTSPS (phospho S2) antibody [EPR18855] (Abcam, Cat# ab193468, RRID:AB_2905557)<br>Mouse monoclonal [SC-35] to SC35 - Nuclear Speckle Marker (Abcam, Cat# ab11826, RRID:AB_298608)<br>Rabbit Histone H3K27ac antibody (pAb) (Active Motif, Cat# 39133, RRID:AB_2561016)<br>Mouse Lamin A/C (E-1) (Santa Cruz Biotechnology, Cat# sc-376248, RRID:AB_10991536)<br>NUP98 (C39A3) Rabbit mAb (Cell Signaling Technology, Cat# 2598, RRID:AB_2267700)<br>Alexa Fluor® 790 AffiniPure Donkey Anti-Mouse IgG (H+L) (Jackson ImmunoResearch Labs, Cat# 715-655-150)<br>Alexa Fluor® 647 AffiniPure Donkey Anti-Rabbit IgG (H+L) (Jackson ImmunoResearch Labs, Cat# 111-605-144)
Validation	Each antibody was experimentally validated by the Bintu/Zhu lab before being used for multi-modal imaging data experiments For each antibody, immunofluorescence images were generated from cultured IMR90 cells and compared to the published dataset (Su et al., 2020 Cell) generated using the same antibody product and using the same cell type (IMR90). The anti-NeuN antibody used in study is a monoclonal antibody (clone A60) used in many of our previous studies. The staining of adult human cortical tissue using the anti-NeuN antibody labeled the expected 1:3 ratio of NeuN+(neuron) and NeuN- (non-neuron) populations. For antibodies used for the imaging experiment, the pattern of the immunofluorescent staining was compared to previous publications to ensure the consistency of antibody specificity.
