# Supplementary material for: Temporally distinct 3D multi-omic dynamics in the developing human brain
Source: Nature. 2024 Oct 9;635(8038):481–9. doi: 10.1038/s41586-024-08030-7 (PMC11560841; doi:10.1038/s41586-024-08030-7)
Supplement: Supplementary file 12 — Antibodies used in multimodal DNA, RNA and protein imaging. [file 41586_2024_8030_MOESM12_ESM.pdf]

Supplementary Table 10. Antibodies used in Multi-modal DNA/RNA/protein imaging.

Antibody	Company	Catalog number	Dilution	Reference
Mouse Histone H3 trimethylated at lysine 9 (H3K9me3)	Diagenode	Cat# C15200146, RRID:AB_2927650	1:300	[1]
Rabbit Anti-RNA polymerase II CTD repeat YSPTSPS (phospho S2) antibody [EPR18855]	Abcam	Cat# ab193468, RRID:AB_2905557	1:400	[1]
Mouse monoclonal [SC-35] to SC35 - Nuclear Speckle Marker	Abcam	Cat# ab11826, RRID:AB_298608	1:400	[2]
Rabbit Histone H3K27ac antibody (pAb)	Active Motif	Cat# 39133, RRID:AB_2561016	1:400	[1]
Mouse Lamin A/C (E-1)	Santa Cruz Biotechnology	Cat# sc-376248, RRID:AB_10991536	1:200	[1]
NUP98 (C39A3) Rabbit mAb	Cell Signaling Technology	Cat# 2598, RRID:AB_2267700	1:200	[3]
Alexa Fluor® 790 AffiniPure Donkey Anti-Mouse IgG (H+L)	Jackson ImmunoResearch Labs	Cat# 715-655-150	1:1000	[2]
Alexa Fluor® 647 AffiniPure Donkey Anti-Rabbit IgG (H+L)	Jackson ImmunoResearch Labs	Cat# 111-605-144	1:1000	[2]

Reference

1. Takei, Y. *et al.* Integrated spatial genomics reveals global architecture of single nuclei. *Nature* **590**, 344–350 (2021).
2. Su, J.-H., Zheng, P., Kinrot, S. S., Bintu, B. & Zhuang, X. Genome-Scale Imaging of the 3D Organization and Transcriptional Activity of Chromatin. *Cell* **182**, 1641–1659.e26 (2020).
3. Lincoln, R. *et al.* A general design of caging-group-free photoactivatable fluorophores for live-cell nanoscopy. *Nat. Chem.* **14**, 1013–1020 (2022).
